# Chirality matters: stereo-defined phosphorothioate linkages at the termini of small interfering RNAs improve pharmacology *in vivo*

**DOI:** 10.1093/nar/gkab544

**Published:** 2021-07-15

**Authors:** Hartmut Jahns, Nate Taneja, Jennifer L S Willoughby, Masaaki Akabane-Nakata, Christopher R Brown, Tuyen Nguyen, Anna Bisbe, Shigeo Matsuda, Matt Hettinger, Rajar M Manoharan, Kallanthottathil G Rajeev, Martin A Maier, Ivan Zlatev, Klaus Charisse, Martin Egli, Muthiah Manoharan

**Affiliations:** Alnylam Pharmaceuticals, 675 W. Kendall St, Cambridge, MA 02142, USA; Alnylam Pharmaceuticals, 675 W. Kendall St, Cambridge, MA 02142, USA; Alnylam Pharmaceuticals, 675 W. Kendall St, Cambridge, MA 02142, USA; Alnylam Pharmaceuticals, 675 W. Kendall St, Cambridge, MA 02142, USA; Alnylam Pharmaceuticals, 675 W. Kendall St, Cambridge, MA 02142, USA; Alnylam Pharmaceuticals, 675 W. Kendall St, Cambridge, MA 02142, USA; Alnylam Pharmaceuticals, 675 W. Kendall St, Cambridge, MA 02142, USA; Alnylam Pharmaceuticals, 675 W. Kendall St, Cambridge, MA 02142, USA; Alnylam Pharmaceuticals, 675 W. Kendall St, Cambridge, MA 02142, USA; Alnylam Pharmaceuticals, 675 W. Kendall St, Cambridge, MA 02142, USA; Alnylam Pharmaceuticals, 675 W. Kendall St, Cambridge, MA 02142, USA; Alnylam Pharmaceuticals, 675 W. Kendall St, Cambridge, MA 02142, USA; Alnylam Pharmaceuticals, 675 W. Kendall St, Cambridge, MA 02142, USA; Alnylam Pharmaceuticals, 675 W. Kendall St, Cambridge, MA 02142, USA; Department of Biochemistry, School of Medicine, Vanderbilt University, Nashville, TN 37232, USA; Alnylam Pharmaceuticals, 675 W. Kendall St, Cambridge, MA 02142, USA

## Abstract

A critical challenge for the successful development of RNA interference-based therapeutics therapeutics has been the enhancement of their *in vivo* metabolic stability. In therapeutically relevant, fully chemically modified small interfering RNAs (siRNAs), modification of the two terminal phosphodiester linkages in each strand of the siRNA duplex with phosphorothioate (PS) is generally sufficient to protect against exonuclease degradation *in vivo*. Since PS linkages are chiral, we systematically studied the properties of siRNAs containing single chiral PS linkages at each strand terminus. We report an efficient and simple method to introduce chiral PS linkages and demonstrate that *R*p diastereomers at the 5′ end and *S*p diastereomers at the 3′ end of the antisense siRNA strand improved pharmacokinetic and pharmacodynamic properties in a mouse model. *In silico* modeling studies provide mechanistic insights into how the *R*p isomer at the 5′ end and *S*p isomer at the 3′ end of the antisense siRNA enhance Argonaute 2 (Ago2) loading and metabolic stability of siRNAs in a concerted manner.

## INTRODUCTION

Small interfering RNAs (siRNAs) can inhibit the expression of disease-causing genes through post-transcriptional gene silencing mediated by the endogenous RNA interference (RNAi) pathway (1–3). Careful optimization of the chemical composition and specific positioning of chemical modifications of siRNAs have led to substantial improvements in intrinsic potency, *in vivo* metabolic stability, and mitigation of innate immune stimulation and off-target effects (4–6). Tissue- and cell-specific delivery of RNAi-based therapeutics has been achieved by conjugation with specific ligands such as the trivalent *N*-acetylgalactosamine (GalNAc) (4–10), which results in hepatocyte-specific delivery, or by delivery using encapsulation with a lipid nanoparticle formulation (11).

**Figure 1.**
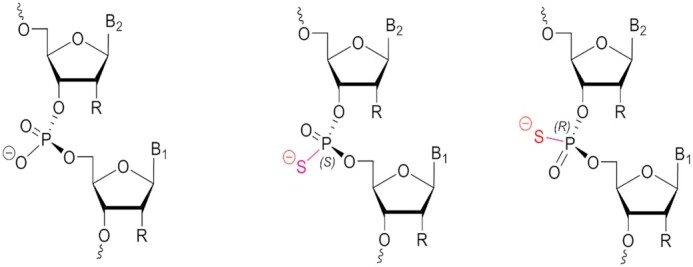
Natural prochiral phosphodiester (PO, left) and phosphorothioate (PS) stereoisomers, *Sp* (middle) and *R*p (right), in a dimeric fragment of an oligonucleotide.

One of the most widely used chemical modifications, which has been critical in the field of oligonucleotide therapeutics, is the phosphorothioate (PS) modification, first introduced by Eckstein in 1966 (12) and further developed for oligonucleotide synthesis by Stec and Zon in 1984 (13). Among the approved 15 oligonucleotide drugs, eight have PS modifications in the backbone (14). Remarkably, the simple replacement of a non-bridging oxygen atom with sulfur in the inter-nucleotide phosphodiester linkage results in considerable nuclease resistance, improves biodistribution, and results in pharmacokinetic advantages (15–20). The PS linkage is chiral with right-handed (*R*p) and left-handed (*S*p) isomers (Figure 1). These two PS diastereomers are expected to differentially impact the physicochemical and biological properties of oligonucleotides, such as nuclease sensitivity, thermodynamic stability of double-stranded structures, and interaction with various enzymes involved in the antisense and RNAi pathways (21). For example, PS oligonucleotides with the *R*p configuration form more stable duplex structures (22) and are more efficient in recruiting RNase H when placed at certain positions in the gap of antisense oligonucleotides than those with the *S*p configuration; oligonucleotides with PS linkages with the *S*p configuration are generally more resistant to nucleases (23). Comparison of stereo-defined to stereo-random PS oligonucleotides with CpG motifs designed for immune activation revealed that the presence of *R*p linkages enhanced the immunostimulatory effects (24,25). One can rationalize that carefully designed oligonucleotides containing optimally positioned stereo-defined PS diastereomers may offer several pharmacological benefits; however, this has been a highly debated topic in the field of antisense oligonucleotides. The magnitude of the benefit may depend on the particular circumstances, and it is unclear how these effects translate *in vivo* and across species (26–28).

Recently, there has been progress toward efficient synthesis and purification of stereo-defined PS oligonucleotides (29,30). A variety of protocols that yield stereo-defined PS linkages in oligonucleotides have been described such as the *H*-phosphonate method (31), use of chiral PS dinucleotide building blocks (32), use of chiral phosphoramidites with the oxazaphospholidine approach (26,29,30,33–35), and use of a tricyclic P(III) chiral auxiliary (36). More recently P(V) chemistry has been elegantly used to synthesize chiral phosphorothioates using limonene precursors (37).

Jahns *et al.* showed that siRNAs with stereoselective bias can be prepared and evaluated (38); however, difficulties in the synthesis have limited the biological evaluation of siRNAs with chiral PS linkages. PS modifications are not widely used in siRNAs as multiple PS linkages inhibit loading into the RNA-induced silencing complex (RISC) (39,40), and multiple PS modifications may lead to undesirable protein binding (41,42). All clinically approved GalNAc-conjugated siRNAs contain six PS linkages. There are two PS linkages at each terminus, with exception of the 3′ end of the sense strand that carries the GalNAc ligand; this configuration results in significant stabilization without compromising *in vivo* activity (7,10).

**Figure 2.**
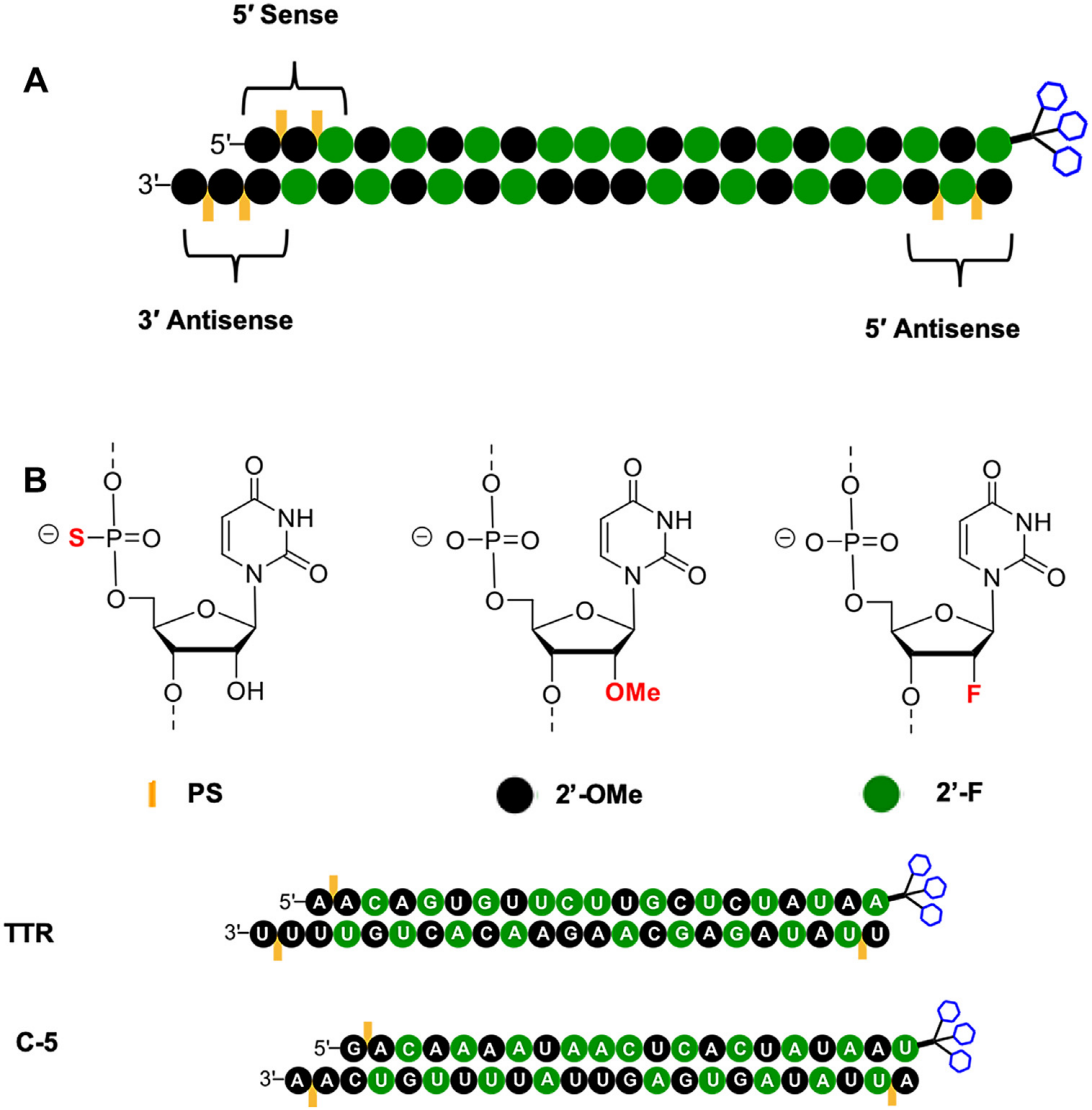
(**A**) The general design of approved GalNAc conjugates with six PS linkages indicating the chemical modifications employed. Chemical modifications other than the terminal PS linkages and GalNAc are for illustration purposes only. (**B**) The design and sequence of GalNAc conjugates described in this work that are modified with three PS linkages. Green nucleotide background indicates 2′-F; black background indicates 2′-OMe. Orange lines indicate location of PS linkages. Blue hexagons represent triantennary GalNAc.

Racemic PS linkages, which are mixtures of *R*p/*S*p diastereoisomers, result in 2*^n^* distinct diastereomeric compounds where *n* refers to the number of linkages in the backbone. Three trivalent GalNAc-conjugated RNAi therapeutics have been approved so far: GIVLAARI^®^ (givosiran, 2019) for treating acute hepatic porphyria (43,44), OXLUMO^®^ (lumasiran, 2020) for the treatment of primary hyperoxaluria type 1 (45), and LEQVIO^®^ (inclisiran, 2020) for treatment of hypercholesterolemia (46–48). Each of the three approved RNAi therapeutics is a mixture of 64 diastereomers resulting from the six stereo-random PS linkages present in the molecule as shown in the schematic in Figure 2A.

In this work, we report an efficient method to introduce stereo-defined PS linkages into siRNAs. To make this systematic study more manageable, we reduced the total number of PS linkages to a single PS on each of the non-GalNAc-conjugated termini and evaluated the properties of the resulting eight chiral siRNAs against two rodent mRNA targets, *transthyretin* (*Ttr*) and *complement factor 5* (*C5*). Both genes are hepatically expressed and are translated into secreted proteins, which can be measured in serum. *Ttr* encodes a homotetrameric serum transport protein whose primary role is to transport vitamin A (retinol) in concert with retinol binding protein (49), whereas *C5* encodes the fifth component of complement, which plays an important role in inflammatory and cell killing processes (50). Given that GalNAc conjugation to siRNAs enables hepatocyte-specific delivery to the liver, both *Ttr* and *C5* are ideal targets, not only because of the robust hepatic gene expression, but because each target is translated into a protein detectable in serum, the quantification of which enables target reduction to be monitored over time.

In line with previous reports (36,51), including our patent disclosure (https://patents.google.com/patent/WO2019126651A1/en), this account provides further demonstration of how chiral PS linkages at the termini of an siRNA can be exploited to optimize RISC loading and metabolic stability. In particular, the findings of the study suggest that careful selection of PS stereochemistry at defined positions of the siRNA antisense strand results in benefits stemming from the different properties of *R*p and *S*p PS isomers. Further, for the first time, the molecular basis for this selectivity is explained based on the analysis of known structures of oligonucleotides, proteins, and enzymes involved.

## MATERIALS AND METHODS

### Synthesis and characterization of stereo-defined dinucleotides

#### General conditions

Thin layer chromatography (TLC) was performed on Merck TLC Silica gel 60 F254. Compounds were visualized under UV light (254 nm) or after spraying with the *p*-anisaldehyde staining solution followed by heating. Flash column chromatography was performed using a Teledyne ISCO Combi Flash system with pre-packed RediSep Teledyne ISCO silica gel cartridges. In the case of acid-sensitive compounds, the cartridge was washed with loading solvent containing 5% triethylamine followed by neat solvent to neutralize the silica prior to loading the crude samples for purification. All moisture-sensitive reactions were carried out under anhydrous conditions using dry glassware, anhydrous solvents, and under argon atmosphere. All commercially available reagents and solvents were purchased from Sigma-Aldrich unless otherwise stated and were used as received. ESI-MS spectra were recorded on a Waters Qtof Premier instrument using the direct flow injection mode. ^1^H, ^13^C, ^19^F, and ^31^P NMR spectra were recorded at 400 or 500, 101 or 126, 376 or 470, and 162 or 202 MHz, respectively (Supplementary Information Section II). Chemical shifts are given in ppm referenced to the solvent residual peak (DMSO-*d*_6_–^1^H: δ at 2.49 ppm and ^13^C δ at 39.5 ppm; acetone-*d*_6_–^1^H: δ at 2.05 ppm and ^13^C δ at 29.8 and 206.3 ppm; CD_3_CN–^1^H: δ at 1.94 ppm and ^13^C δ at 1.32 and 118.3 ppm). The representative example of synthesis and characterization of an asymmetric 2′-OMe/2′-F stereo-defined dinucleotide pair (**11a** and **11b**) is shown in Scheme 2. See Supplementary Information Section II for synthetic details of all other stereo-defined dinucleotides.

To a mixture of 2′-*O*-Me-U-CE phosphoramidite **1** (5 g, 6.57 mmol) and 2′-deoxy-3′-*O*-TBS-2′-fluorouridine **8** (2.84 g, 7.89 mmol) in CH_2_Cl_2_ (10 mL) was added 5-Ethylthio-1H-Tetrazole (ETT) (0.25 M in CH_3_CN; 52.6 mL, 13.1 mmol). The reaction mixture was stirred at room temperature overnight. Phenylacetyl disulfide (PADS) (2.98 g, 9.86 mmol) and 2.6-lutidine (1.15 mL, 9.86 mmol) were added, and the reaction mixture was stirred at room temperature for 4 h. The reaction mixture was concentrated under vacuum. The residue was dissolved in Na_2_S_2_O_3_ (aq.) and CH_2_Cl_2_. The aqueous layer was washed with CH_2_Cl_2_ and ethyl acetate. The combined organic layer was washed with brine and dried with Na_2_SO_4_ and concentrated under vacuum. The crude residue was purified by column chromatography on silica gel (45–55% ethyl acetate (5% MeOH) in hexanes). Compound **9a** (faster migrating isomer on TLC) was isolated as a white foam (3.16 g, 46%), and compound **9b** (slower migrating isomer on TLC) was isolated as a white foam (2.83 g, 41%).

Compound **9a**: ^1^H NMR (400 MHz, DMSO-*d*_6_): δ 11.45 (d, *J* = 2.1 Hz, 1H), 11.41 (d, *J* = 2.1 Hz 1H), 7.65 (d, *J* = 8.2 Hz, 1H), 7.59 (d, *J* = 8.2 Hz, 1H), 7.38–7.22 (m, 9H), 6.91–6.88 (m, 4H), 5.88–5.82 (m, 2H), 5.61 (dd, *J* = 8.0 and 2.1 Hz, 1H), 5.41 (dd, *J* = 8.0 and 2.1 Hz, 1H), 5.28–5.09 (m, 2H), 4.41–4.04 (m, 8H), 3.73 (s, 6H), 3.37 (s, 3H), 3.35–3.27 (m, 2H), 2.85–2.81 (m, 2 H), 0.86 (s, 9H), 0.10 (s, 3H), 0.09 (s, 3H). ^13^C NMR (101 MHz, DMSO-*d*_6_): δ 163.09, 162.77, 158.18, 150.30, 150.12, 144.25, 141.22, 140.16, 135.05, 134.87, 129.75, 127.89, 127.73, 126.87, 117.85, 113.25, 102.17, 101.89, 92.79, 90.93, 89.42, 89.07, 86.43, 86.24, 81.24, 81.20, 80.26, 80.18, 79.87, 79.83, 75.07, 75.02, 69.17, 69.01, 66.63, 66.59, 63.09, 63.05, 62.30, 58.02, 55.03, 25.47, 18.81, 18.72, 17.64, –4.98, –5.29. ^19^F NMR (470 MHz, DMSO-*d*_6_): δ –200.59, –200.63, –200.68, –200.70, –200.75, –200.79. ^31^P NMR (162 MHz, DMSO-*d*_6_): δ 66.96. HRMS calc. for C_49_H_59_FN_5_NaO_14_PSSi [M + Na]^+^ 1074.3162, found 1074.3184.

Compound **9b**: ^1^H NMR (400 MHz, DMSO-*d*_6_): δ 11.46 (d, *J* = 2.1 Hz, 1H), 11.43 (d, *J* = 2.1 Hz, 1H), 7.71 (d, *J* = 8.1 Hz, 1H), 7.56 (d, *J* = 8.1 Hz, 1H), 7.39–7.21 (m, 9H), 6.90–6.87 (m, 4H), 5.86–5.80 (m, 2H), 5.60 (dd, *J* = 8.1 and 2.1 Hz, 1H), 5.36 (dd, *J* = 8.1 and 2.1 Hz, 1H), 5.16–5.12 (m, 2H), 4.41–4.33 (m, 1H), 4.25–4.18 (m, 6H), 3.99–3.97 (m, 1H), 3.73 (s, 6H), 3.41 (s, 3H), 3.37–3.29 (m, 2H), 2.94 (t, *J* = 5.8 Hz, 1H), 0.85 (s, 9H), 0.09 (s, 3H), 0.09 (s, 3H). ^13^C NMR (126 MHz, DMSO-*d*_6_): δ 163.09, 162.82, 158.17, 150.25, 150.10, 144.29, 141.12, 140.21, 135.04, 134.82, 129.79, 129.77, 127.87, 127.72, 126.83, 118.05, 113.22, 102.03, 101.84, 92.69, 91.20, 89.39, 89.10, 86.69, 86.23, 81.03, 80.98, 80.15, 80.07, 80.05, 80.02, 74.70, 74.67, 68.94, 68.81, 66.09, 66.06, 63.42, 63.38, 61.88, 58.04, 55.01, 55.00, 25.48, 18.86, 18.79, 17.62, –4.95, –5.23. ^19^F NMR (376 MHz, DMSO-*d*_6_): δ –199.59, –199.65, –199.70, –199.73, –199.79, –199.84. ^31^P NMR (162 MHz, DMSO-*d*_6_): δ 67.91. HRMS calc. for C_49_H_59_FN_5_NaO_14_PSSi [M + Na]^+^ 1074.3162, found 1074.3197.

To a solution of compound **9a** (700 mg, 0.665 mmol) in THF (6.7 mL) was added dropwise Et_3_N·3HF (0.325 mL, 2.00 mmol), and the mixture was stirred at room temperature for 15 h. The reaction mixture was concentrated under vacuum. The crude residue was purified by column chromatography on silica gel (75–100% ethyl acetate (5% MeOH) in hexanes) to obtain compound **10a** as a white foam (477 mg, 76%). ^1^H NMR (400 MHz, DMSO-*d*_6_): δ 11.45 (d, *J* = 1.8 Hz, 1H), 11.41 (d, *J* = 1.8 Hz, 1H), 7.67 (d, *J* = 8.1 Hz, 1H), 7.57 (d, *J* = 8.1 Hz, 1H), 7.38–7.23 (m, 9H), 6.91–6.88 (m, 4H), 5.88–5.80 (m, 3H), 5.59 (dd, *J* = 8.1 and 2.0 Hz, 1H), 5.39 (dd, *J* = 8.1 and 2.0 Hz, 1H), 5.19–5.04 (m, 2H), 4.42–4.38 (m, 1H), 4.24–4.03 (m, 7H), 3.73 (s, 6H), 3.38–3.28 (m, 5H), 2.90–2.78 (m, 2 H). ^13^C NMR (101 MHz, DMSO-*d*_6_): δ 163.05, 162.80, 158.19, 150.30, 150.10, 144.26, 140.89, 140.22, 135.07, 134.86, 129.77, 127.91, 127.75, 126.88, 117.93, 113.27, 102.13, 101.89, 93.68, 91.84, 89.00, 88.65, 86.56, 86.26, 81.18, 81.12, 80.21, 80.12, 79.99, 79.95, 74.92, 74.88, 68.14, 67.97, 67.38, 67.33, 63.05, 63.00, 62.18, 58.02, 55.04, 18.80, 18.71. ^19^F NMR (470 MHz, DMSO-*d*_6_): δ –200.74, –200.78, –200.83, –200.85, –200.89, –200.94. ^31^P NMR (202 MHz, DMSO-*d*_6_): δ 67.93. HRMS calc. for C_43_H_45_FN_5_NaO_14_PS [M + Na]^+^ 960.2298, found 960.2305.

To a solution of compound **9b** (500 mg, 0.475 mmol) in THF (5 ml) was added dropwise Et_3_N·3HF (0.232 ml, 1.43 mmol), and the mixture was stirred at room temperature for 40 h. The reaction mixture was concentrated under vacuum. The crude residue was purified by column chromatography on silica gel (75–100% ethyl acetate (5% MeOH) in hexanes) to obtain compound **10b** as a white foam (405 mg, 91%). ^1^H NMR (500 MHz, DMSO-*d*_6_): δ 11.44 (d, *J* = 2.0 Hz, 1H), 11.40 (d, *J* = 2.0 Hz, 1H), 7.69 (d, *J* = 8.2 Hz, 1H), 7.54 (d, *J* = 8.2 Hz, 1H), 7.38–7.22 (m, 9H), 6.90–6.88 (m, 4H), 5.86–5.80 (m, 3H), 5.57 (dd, *J* = 8.2 and 2.0 Hz, 1H), 5.36 (dd, *J* = 8.2 and 2.0 Hz, 1H), 5.16–5.04 (m, 2H), 4.26–4.12 (m, 7H), 4.00–3.97 (m, 1H), 3.73 (s, 6H), 3.41 (s, 3H), 3.31–3.31 (m, 2H), 2.95–2.92 (m, 2 H). ^13^C NMR (126 MHz, DMSO-*d*_6_): δ 163.07, 162.86, 158.18, 158.18, 150.27, 150.10, 144.31, 140.83, 140.25, 135.07, 134.87, 129.79, 127.91, 127.75, 126.86, 118.14, 113.26, 102.05, 101.86, 93.58, 92.11, 89.01, 88.73, 86.71, 86.27, 81.06, 81.01, 80.10, 80.07, 80.04, 74.65, 74.62, 67.97, 67.84, 66.95, 66.91, 63.39, 63.35, 61.92, 58.05, 55.03, 55.03, 18.84, 18.77. ^19^F NMR (470 MHz, DMSO-*d*_6_): δ -200.46, –200.50, –200.55, –200.57, –200.62, –200.66. ^31^P NMR (202 MHz, DMSO-*d*_6_): δ 68.20. HRMS calc. for C_43_H_45_FN_5_NaO_14_PS [M + Na]^+^ 960.2298, found 960.2322.

To a solution of compound **10a** (1.37 g, 1.46 mmol) and ETT (0.25 M in CH_3_CN; 8.76 ml, 2.19 mmol) in CH_2_Cl_2_ (5 ml) was added dropwise 2-cyanoethyl *N*,*N*,*N′*,*N′*-tetraisopropylphosphorodiamidite (0.557 ml, 1.75 mmol) at 0°C. The mixture was stirred at room temperature for 3 h. The reaction mixture was quenched with saturated NaHCO_3_ (aq.) and washed with saturated NaHCO_3_ (aq.), water, brine, dried (Na_2_SO_4_) and concentrated under vacuum. The crude residue was purified by column chromatography on silica gel (60–100% ethyl acetate in hexanes) to obtain compound **11a** as a white foam (1.22 g, 74%). ^1^H NMR (500 MHz, CD_3_CN): δ 9.67 (brs, 2H), 7.70–7.67 (m, 1H), 7.52–7.49 (m, 1H), 7.45–7.44 (m, 2H), 7.35–7.24 (m, 7H), 6.90–6.88 (m, 4H), 5.93–5.84 (m, 2H), 5.65 (d, *J* = 8.1 Hz, 1H), 5.58 (dd, *J* = 8.1 and 5.0 Hz, 1H), 5.26–5.12 (m, 2H), 4.59–4.28 (m, 5H), 4.22–4.07 (m, 3H), 3.89–3.77 (m, 8H), 3.70–3.61 (m, 2H), 3.49–3.44 (m, 5H), 2.73–2.65 (m, 4H), 1.21–1.18 (m, 12H). ^13^C NMR (101 MHz, CD_3_CN): δ 164.31, 164.29, 164.12, 159.78, 151.45, 151.43, 151.19, 151.16, 145.42, 142.06, 141.89, 140.96, 136.25, 136.10, 131.13, 131.12, 129.09, 128.97, 128.08, 119.54, 114.19, 103.16, 103.14, 103.08, 103.01, 94.43, 93.90, 93.90, 92.56, 92.01, 92.00, 91.30, 91.2, 90.94, 90.85, 87.93, 87.84, 82.62, 82.56, 82.50, 82.27, 82.25, 81.20, 81.20, 81.10, 81.10, 80.80, 80.80, 80.70, 80.70, 75.83, 75.79, 75.75, 70.89, 70.71, 70.59, 70.43, 67.80, 67.72, 67.46, 67.41, 64.22, 64.17, 62.80, 62.76, 59.90, 59.71, 59.52, 59.19, 55.96, 44.30, 44.26, 44.17, 44.14, 25.12, 25.05, 24.97, 24.93, 24.90, 24.86, 21.06, 20.99, 20.92, 20.06, 19.97. ^19^F NMR (470 MHz, CD_3_CN): δ –199.33, –199.35, –199.37, –199.39, –199.41, –199.43, –199.44, –199.46, –199.48, –199.50, –199.52, –199.54, –199.93, –199.95, –199.97, –199.99, –200.02, –200.03, –200.04, –200.06, –200.09, –200.10, –200.13, –200.14. ^31^P NMR (202 MHz, CD_3_CN): δ 152.11, 152.08, 152.04, 152.00, 68.52, 68.51. HRMS calc. for C_52_H_63_FN_7_O_15_P_2_S [M + H]^+^ 1138.3557, found 1138.3546.

To a solution of compound **10b** (1.16 g, 1.24 mmol) and ETT (0.25 M in CH_3_CN; 7.42 ml, 1.86 mmol) in CH_2_Cl_2_ (5 ml) was added dropwise 2-cyanoethyl *N*,*N*,*N′*,*N′*-tetraisopropylphosphorodiamidite (0.471 ml, 1.48 mmol) at 0°C. The mixture was stirred at room temperature overnight. The reaction mixture was quenched with saturated NaHCO_3_ (aq.) and washed with saturated NaHCO_3_ (aq.), water, and brine, dried (Na_2_SO_4_), and concentrated under vacuum. The crude residue was purified by column chromatography on silica gel (60–100% ethyl acetate in hexanes) to obtain compound **11b** as a white foam (1.07 g, 76%). ^1^H NMR (500 MHz, CD_3_CN): δ 9.42 (brs, 2H), 7.65 (d, *J* = 8.2 Hz, 1H), 7.45–7.43 (m, 2H), 7.40–7.24 (m, 8H), 6.89–6.87 (m, 4H), 5.92–5.90 (m, 1H), 5.79–5.72 (m, 1H), 5.58 (dd, *J* = 8.2 and 2.6 Hz, 1H), 5.34–5.32 (m, 1H), 5.25–5.11 (m, 2H), 4.58–4.14 (m, 7H), 4.10–4.08 (m, 1H), 3.87–3.74 (m, 8H), 3.68–3.60 (m, 2H), 3.49–3.49 (m, 3H), 3.44–3.37 (m, 2H), 2.84–2.80 (m, 2H), 2.66–2.64 (m, 2 H), 1.19–1.17 (m, 12H). ^13^C NMR (101 MHz, CD_3_CN): δ 164.11, 164.10, 163.93, 159.82, 159.80, 151.49, 151.47, 151.09, 151.07, 145.51, 145.49, 142.32, 142.12, 140.88, 136.25, 136.11, 131.17, 131.15, 129.08, 128.99, 128.08, 119.55, 119.51, 118.57, 118.52, 114.22, 103.17, 103.06, 102.99, 94.50, 94.00, 93.90, 92.70, 92.10, 92.10, 91.76, 91.39, 87.96, 87.74, 87.71, 82.68, 82.65, 82.62, 82.58, 82.41, 82.38, 81.03, 81.02, 80.92, 80.92, 80.61, 80.61, 80.50, 80.50, 75.95, 75.90, 70.53, 70.51, 70.40, 70.30, 70.20, 70.10, 67.30, 67.2, 67.0, 66.92, 64.46, 64.41, 62.79, 59.94, 59.75, 59.69, 59.50, 59.20, 55.97, 44.30, 44.27, 44.17, 44.14, 25.13, 25.05, 24.97, 24.94, 24.91, 24.90, 24.86, 21.06, 21.01, 20.99, 20.94, 20.14, 20.06. ^19^F NMR (470 MHz, CD_3_CN): δ –198.40, –198.42, –198.45, –198.47, –198.49, –198.51, –198.53, –198.56, –198.58, –198.60, –198.62, –198.83, –198.85, –198.88, –198.89, –198.92, –198.93, –198.95, –198.96, –198.99, –199.00, –199.03, –199.05. ^31^P NMR (202 MHz, CD_3_CN): δ 151.99, 151.99, 151.95, 151.94, 68.72, 68.66. HRMS calc. for C_52_H_63_FN_7_O_15_P_2_S [M + H]^+^ 1138.3557, found 1138.3550.

### General conditions for oligonucleotide synthesis

Oligonucleotides were synthesized on a MerMade-12 DNA/RNA synthesizer at 10-μmol scale using a modified synthesis cycle provided from the instrument vendor. Sterling solvents and reagents from Glen Research, 500-Å controlled pore glass (CPG) solid supports from Prime Synthesis, 2′-deoxy 3′-phosphoramidites from Thermo, and 2′-*O*-methyl (2′-*O*-Me), 2′-deoxy-2′-fluoro (2′-F) ribonucleoside 3′-phosphoramidites from Hongene Biotech were all used as received. *N*4-Acetylcytosine, *N*6-benzoyladenine, and *N*2-isobutyrylguanine were used as the exocyclic amine-protected nucleobases in all phosphoramidites used. These more stable exocyclic amine protections were chosen to ensure maintenance of the exocyclic amine protection during the solution-phase dimer synthesis. Stereo-defined dinucleotide 3′ phosphoramidites were manually coupled to support or to the elongating oligonucleotide using standard conditions. A single 15-min coupling time was sufficient. GalNAc CPG support was prepared and used as previously described (7). Low-water content acetonitrile was purchased from EMD Millipore. DNA and RNA oligonucleotides were synthesized using modified synthesis cycles, based on those provided with the instrument. For detritylation, 3% dichloroacetic acid in dichloromethane (DCM) was used. A solution of 0.6 M 5-(S-ethylthio)-1H-tetrazole (ETT) in acetonitrile was used for the activation of phosphoramidites. The phosphoramidite solutions were 0.15 M in anhydrous acetonitrile with 15% DMF as a co-solvent for 2′-OMe uridine and cytidine. Oxidizing and sulfurizing reagents were 0.02 M I_2_ in THF/pyridine/water and 0.09 M *N,N*-dimethyl-*N′*-(3-thioxo-3*H*-1,2,4-dithiazol-5-yl)methanimidamide in pyridine, respectively.

After completion of the solid-phase syntheses, the CPG support was washed with 5% (v/v) piperidine in anhydrous acetonitrile three times with 5-min holds after each addition. The support was then washed with anhydrous acetonitrile and dried with argon. The oligonucleotides were then incubated with 28–30% (w/v) NH_4_OH, at 35°C for 20 h. The solvent was collected by filtration, and the support was rinsed with water prior to analysis. Oligonucleotide solutions of approximately 1 OD_260_ units/ml were used for analysis of the crudes, and 30–50 μl of solution were injected. LC/ESI-MS was performed on an Agilent 6130 single-quadrupole LC/MS system using an XBridge C8 column (2.1 × 50 mm, 2.5 μm) at 75°C. Buffer A consisted of 200 mM 1,1,1,3,3,3-hexafluoro-2-propanol and 16.3 mM triethylamine in water, and buffer B was 100% methanol. Oligonucleotides were eluted with 0 to 40% gradient of buffer B over 10 min. All oligonucleotides were purified and desalted, and further annealed to form GalNAc-siRNAs as previously described (7).

### *In vitro* gene silencing experiments

#### Cell isolation and siRNA treatment

*In vitro* gene silencing assays were performed using primary mouse hepatocytes freshly isolated as previously reported (7). Free uptake and transfection assays were performed using protocols previously described (52,53). Each siRNA concentration was analyzed in duplicate. For transfection assays, 5 μl siRNA at indicated concentrations were mixed with 4.9 μl of Opti-MEM (LifeTech, cat #31985–062) and 0.1 μl of Lipofectamine RNAiMax (Invitrogen, cat #13778150) per well of a 384-well plate. After incubation at room temperature for 15 min, 40 μl of William's E Medium (Life Tech, cat #A12176-01) containing ∼5×10^3^ primary mouse hepatocytes were added to the wells. Cells were incubated for 24 h at 37°C in 5% CO_2_ prior to RNA purification. For free uptake experiments, 5 μl siRNA at indicated concentrations were mixed with 5 μl Opti-MEM (LifeTech, cat #31985-062) per well of a 384-well plate. After 15 min, 40 μl of William's E Medium (Life Tech, cat #A12176-01) or EMEM medium (ATCC) containing ∼5 × 10^3^ cells were added to the siRNA mixture. Cells were incubated for 24 h at 37°C in 5% CO_2_ prior to RNA purification.

#### RNA isolation

RNA was isolated using an automated protocol on a BioTek-EL406 platform using DYNABEADs (Invitrogen, cat #61012) as per the manufacturer's protocol and using the manufacturer's reagents. Briefly, 50 μl of Lysis/Binding Buffer and 25 μl of lysis buffer containing 3 μl of magnetic beads were added to each well. Plates were incubated on an electromagnetic shaker for 10 min at room temperature, and then magnetic beads were captured and the supernatant was removed. Bead-bound RNA was then washed twice with 150 μl/well of wash Buffer A and once with 150 μl/well wash Buffer B. Beads were then washed with 150 μl Elution Buffer, re-captured, and supernatant was removed.

#### Target mRNA analysis using real-time RT-PCR

cDNA synthesis was performed using an ABI kit (cat #4374967). To wells of a 384-well plate containing RNA isolated using DYNABEADS, 10 μl of a master mix containing 1 μl 10× Buffer, 0.4 μl 25× dNTPs, 1 μl 10× random primers, 0.5 μl reverse transcriptase, 0.5 μl RNase inhibitor, and 6.6 μl of H_2_O were added. Plates were sealed, mixed, and incubated on an electromagnetic shaker for 10 min at room temperature, followed by 2 h at 37°C.

To each well of a 384-well plate (Roche, cat #04887301001), 2 μl of cDNA was added to a master mix containing 0.5 μl of mouse GAPDH TaqMan Probe (cat #4352339E), 0.5 μl C5 mouse probe (cat #Mm00439275_m1), 5 μl LightCycler 480 probe master mix, and 2 μl of H_2_O. Real-time PCR was done in a LightCycler 480 system (Roche). To calculate relative fold change, data were analyzed using the ΔΔCt method and normalized to assays performed with cells transfected with a control siRNA.

### *In vivo* gene silencing experiments

#### Animals

All procedures using mice were conducted by certified laboratory personnel using protocols consistent with local, state, and federal regulations. Experimental protocols were approved by the Institutional Animal Care and Use Committee, the Association for Assessment and Accreditation of Laboratory Animal Care International (accreditation number: 001345), and the office of Laboratory Animal Welfare (accreditation number: A4517-01). Sample numbers were determined to allow for confidence in the resulting data set utilizing the least number of animals, as required in accordance with IACUC guidelines. All animals were acclimated in-house for 48 h prior to study start. Female C57BL/6 mice ∼8 weeks of age were obtained from Charles River Laboratories and randomly assigned to each group. Animals were dosed subcutaneously with siRNA duplex diluted in PBS (Gibco) or with vehicle control (PBS). Solutions were stored at 4°C until time of injection. Animals were sacrificed at either 5 or 7 days post dose. Livers were harvested and snap frozen for analysis.

#### Target mRNA analysis

RNA isolation using the QIAzol reagent (Qiagen) was performed by adding the reagent either to cells immediately following a PBS wash or to frozen cell pellets collected after a PBS wash and snap frozen at –80°C. Alternatively, RNA was isolated from samples using the Qiagen RNAeasy kit following the manufacturer's instructions. RNA was quantified using real-time RT-PCR as described above.

#### Serum collection

Blood was collected utilizing the retro-orbital eye bleed procedure prior to the first dose (pre-dose) and then at various time points post dose. For this procedure, the mice were anesthetized using isoflurane. Heparin-coated capillary tubes (Fisher Scientific) were inserted into the posterior corner of the mouse eye; the tube was inserted at a 45° angle to ∼1 cm and rotated until the blood from the retro-orbital sinus was released. Approximately 200 μl was collected from the left eye of each mouse according to the IACUC protocol for blood collection. The blood was collected in Becton Dickinson (BD) serum separator tubes. Serum samples were kept at room temperature for 1 h and then spun in a microcentrifuge at 22 × g at room temperature for 10 min. Serum was transferred to 1.5-ml microcentrifuge tubes. Serum collected for the analysis of transthyretin was stored at –80°C until sample processing. Serum collected for the analysis of circulating C5 was kept at room temperature for 15 min and then immediately transferred to 4°C prior to spinning in a microcentrifuge at 22 × g at room temperature for 10 min.

#### Quantification of serum transthyretin levels

Serum samples were diluted 1:4000 and assayed using a commercially available kit from ALPCO (cat #41-PALMS-E01) as per the manufacturer's instructions. Protein concentrations (μg/ml) were determined by use of a purified TTR standard prepared in-house.

#### Quantification of serum C5 levels

A C5 ELISA mouse-reactive assay was developed in house. The primary antibody was mouse cross-reactive goat-anti-human C5 (Complement Technologies, cat #A220) and the secondary antibody was bovine anti-goat IgG-HRP (H&L) (Jackson ImmunoResearch, cat #805–035-180). The assay was developed using a TMB substrate kit and the reaction was stopped using sulfuric acid. The serum samples were diluted 1:5000 for analysis.

### Enzymatic stability assays

#### Snake venom phosphodiesterase stability assay

Modified oligonucleotide was added to a final concentration of 0.1 mg/ml to a solution of 50 mM Tris–HCl (pH 7.2) and 10 mM MgCl_2_. Snake venom phosphodiesterase (SVPD) (Worthington, cat #LS003926) was added to the mixture at 750 mU/ml. Aliquots were analyzed every hour for 24 h by injection onto a Dionex DNAPac PA200 column (4 mm × 250 mm) maintained at 30°C run at a flow rate of 1 ml/min with a gradient of 40–55% Buffer B over 7.5 min. Buffer A was 20 mM sodium phosphate, 15% acetonitrile, pH 11; Buffer B was Buffer A containing 1 M sodium bromide (pH 11). The area under the peak corresponding to full-length oligonucleotide was normalized to the area from the 0 h time point. First-order decay kinetics were assumed in calculation of half-lives. A control oligo-2′-deoxynucleotide, dT_20_ with a single 3′-terminal PS linkage, was analyzed each day. Enzyme was prepared as a stock of 1000 mU/ml, aliquoted into 1 ml tubes, and stored at –20°C. A new aliquot was used each week.

#### Phosphodiesterase II stability assay

Modified oligonucleotide was added at 0.1 mg/ml to a solution of 50 mM sodium acetate (pH 6.5) and 10 mM MgCl_2_. Phosphodiesterase II (PDII) from bovine spleen (Worthington, cat #LS003 602) was added to the mixture at 500 mU/ml. Aliquots were analyzed every hour for 24 h by injection onto a Dionex DNAPac PA200 column (4 mm × 250 mm) at 30°C run at a flow rate of 1 ml/min with a gradient of 37–52% Buffer B over 7.5 min. Buffer A was 20 mM sodium phosphate, 15% acetonitrile, pH 11; Buffer B was Buffer A containing 1 M sodium bromide (pH 11). The area under the peak corresponding to full-length oligonucleotide was normalized to the area from the 0 h time point. First-order decay kinetics were assumed in calculation of half-lives. A control oligo-2′-deoxynucleotide, dT_20_ with a single 5′-terminal PS linkage, was analyzed each day. Enzyme was prepared as a stock of 2000 mU/ml, aliquoted into 1 ml tubes, and stored at –20°C. A new aliquot was used each week.

### RISC loading experiment

#### Quantification of siRNAs in whole liver and associated with Argonaute 2 (Ago2)

C57BL/6 female mice (*n* = 3 for each treatment) were sacrificed on either day 4 (following a single subcutaneous dose of 2.5 mg/kg of *C5*-targeted siRNA) or day 7 (following a single subcutaneous dose of 2 mg/kg of *Ttr*-targeted siRNA). Livers were snap frozen in liquid nitrogen and ground into powder for further analysis. Total siRNA liver levels were measured by reconstituting liver powder at 10 mg/ml in PBS containing 0.25% Triton-X 100. The tissue suspension was further ground with 5-mm steel grinding balls at 50 cycles/s for 5 min in a tissue homogenizer (Qiagen TissueLyser LT) at 4°C. Homogenized samples were then heated at 95°C for 5 min, briefly vortexed, and allowed to rest on ice for 5 min. Samples were then centrifuged at 21 000 × g for 5 min at 4°C. The siRNA-containing supernatants were transferred to new tubes. siRNA sense and antisense strand levels were quantified by stem-loop reverse transcription followed by Taqman PCR (SL-RT QPCR) based on a previously published method (54,55).

Ago2-bound siRNA from mouse liver was quantified by preparing liver powder lysates at 100 mg/ml in lysis buffer (50 mM Tris–HCl, pH 7.5, 150 mM NaCl, 2 mM EDTA, 0.5% Triton-X 100) supplemented with freshly added protease inhibitors (Sigma-Aldrich, cat #P8340) at 1:100 dilution and 1 mM PMSF (Life Technologies). Total liver lysate (10 mg) was used for each Ago2 immunoprecipitation and control immunoprecipitation. Anti-Ago2 antibody was purchased from Wako Chemicals (Clone No. 2D4). Control mouse IgG was from Santa Cruz Biotechnology (cat #sc-2025). Protein G Dynabeads (Life Technologies) were used to precipitate antibodies. Ago2-associated siRNAs were eluted by heating (50 μl PBS, 0.25% Triton; 95°C, 5 min) and quantified by SL-RT QPCR as described (54,55).

#### In vitro quantification of human Ago2-associated siRNAs

Ago2-bound siRNA was quantified in HEK293 cell lysates overexpressing FLAG-HA-tagged human Ago2 (56). HEK293 lysates were prepared by incubating cells at room temperature for 30 min in lysis buffer (100 mM KCl, 20 mM HEPES pH 7.5, 0.5 mM TCEP, 0.05% NP-40) with gentle rocking. Lysates were cleared by centrifugation (25 000 ×g, 20 min, 4°C), and supernatant aliquots were frozen on dry ice before storage at –80°C. Each 70 μl *in vitro* loading reaction contained 30 mM HEPES (pH 7.5), 100 mM NaCl, 5 mM MgCl_2_, 1 mM DTT, 0.03 mg/ml creatine kinase, 25 mM creatine phosphate, 1 U/μl Superase-In nuclease inhibitor, 1 mM ATP, 0.2 mM GTP, 35 μl HEK293 cell lysate and 7 μl of 200 nM siRNA duplex (1.4 pmol total). Loading reactions were incubated at 37°C for 4 h. After incubation with 430 μl of 20 mg/ml QAE 50 anion exchange resin (GE Healthcare) for 15 min at 4°C, samples were passed through a cellulose acetate filter column to remove the QAE resin prior to addition of 60 μl of Protein G Dynabeads (Life Technologies), 3 μl of anti-HA antibody (Cell Signaling, cat #3724S), and 600 μl of lysis buffer supplemented with freshly added protease inhibitors (Sigma-Aldrich, cat #P8340) at 1:100 dilution and 1 mM PMSF (Life Technologies). After overnight incubation at 4°C, Ago2-associated siRNAs were eluted by heating (50 μl PBS, 0.25% Triton; 95°C, 5 min) and quantified by SL-RT QPCR as described (54,55).

## RESULTS

### Synthesis of stereo-defined PS-containing GalNAc–siRNA conjugates

We investigated two orthogonal methods for the synthesis of stereo-defined PS-containing GalNAc–siRNA conjugates (Table 1). The siRNAs evaluated contained three PS linkages, one at the 5′ end of the sense strand and one at each end (3′ and 5′) of the antisense strand. To simplify the presentation of our data, we refer to PS-modified siRNAs in a three-letter format, S/AS: 5′X/5′X-3′X. The first letter indicates the absolute configuration of the PS linkage at the 5′ end of the sense strand, and the second and third letters represent the configurations of the PS linkages at the 5′ and 3′ ends of the antisense strand, respectively. Use of the letter X indicates a stereo-random PS linkage, whereas S and R identify *S*p or *R*p PS diastereomers, respectively.

**Table 1.** Characteristics of siRNAs synthesized for this study

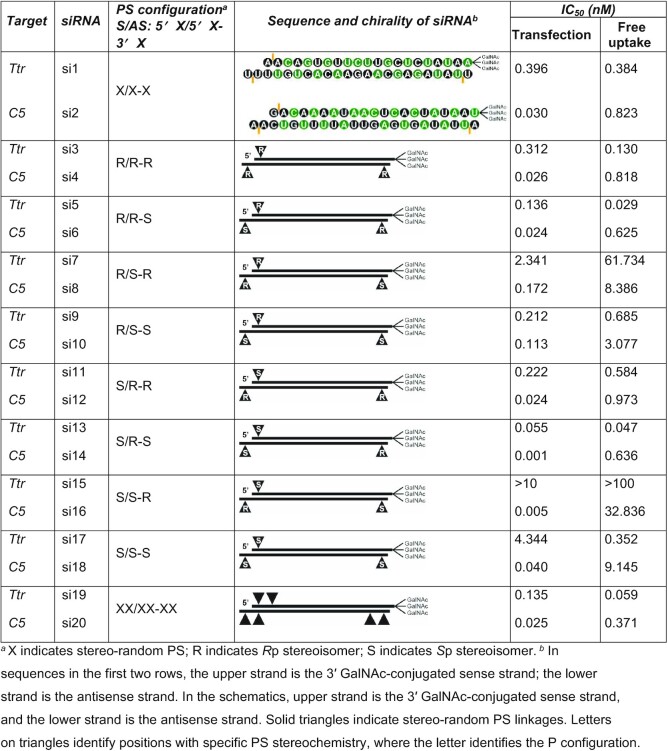

First, using the ‘purification approach’, we separated the single-stranded oligonucleotides containing the desired PS stereoisomers by ion exchange chromatography after stereo-random synthesis but before annealing into double-stranded siRNAs. We hoped that this approach, due to the limited number of PS linkages (one in the sense strand or two in the antisense strand), would result in the purification of all possible stereo-defined oligonucleotide isomers. Although we were able to easily separate the isomers of the sense strands containing a single 5′ PS (Supplementary Figures S1–S4), the isomers of the antisense strands, containing two PS linkages, were more difficult to resolve by HPLC (Supplementary Figures S5–S8). Final stereo-purity for the oligonucleotides was approximately 90% by HPLC, with the exception of oligonucleotides with 5′*R*p–3′*S*p (antisense strand of si6 and si14) and 5′*S*p–3′*S*p (antisense strand of si10 and si18) configurations, which had purities of 77% and 80%, respectively (Supplementary Figure S8). Methods of assignment of configurations for these compounds can be found in Supplementary Information (Supplementary Tables S1–S3 and Figures S9–S20).

**Scheme 1.**
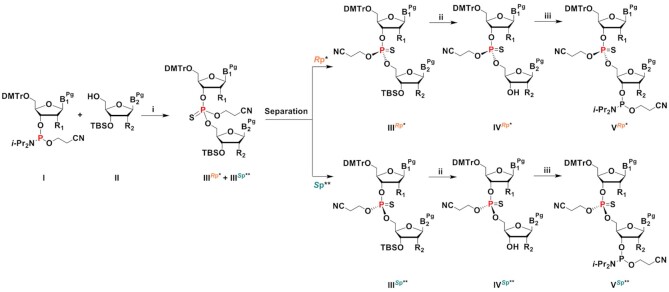
Synthesis and separation of chiral PS dinucleotides as building blocks for oligonucleotide synthesis. B = nucleobase, uracil (U), adenine (A), guanine (G); Pg = protecting group, benzoyl (Bz) for A, *N*-isobutyryl (*i*-Bu) for G; R_1_, R_2_ = F and/or OMe; DMTr = dimethoxytrityl; TBS = *tert*-butyldimethylsilyl; CE = β-cyanoethyl; * except dimer gsa (*S*p); ** except dimer gsa (*R*p) (i) (a) 5-ethylthio-1*H*-tetrazole (ETT), solvent, rt; (b) phenylacetyl disulfide (PADS), 2,6-lutidine, rt; (ii) Et_3_N·3HF, THF, rt to 50°C; (iii) 2-cyanoethyl *N*,*N*,*N*',*N*'-tetraisopropylphosphorodiamidite, 5-ethylthio-1*H*-tetrazole (ETT), solvent, 0°C to rt. Synthesis of each unique stereo-defined dinucleotide according to this scheme is described in detail in Materials and Methods and in Supplementary Information: III^Rp*^ refers to a, whereas the III^Sp**^ refers to b of compounds 3, 9, 15, 21, 27, 33 and 39; IV^Rp*^ refers to a, whereas the IV^Sp**^ refers to b of compounds 4, 10, 16, 22, 28, 34, and 40; V^Rp*^ refers to a, whereas the V^Sp**^ refers to b compounds 5, 11, 17, 23, 29, 35 and 41.

**Figure 3.**
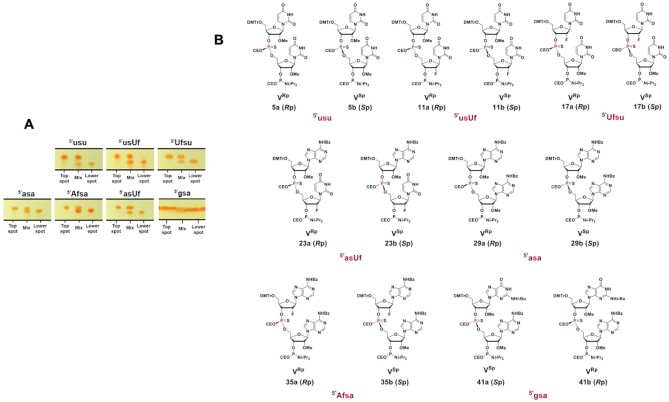
(**A**) Separation of PS dinucleotide diastereomers (III^*R*p*^ and III^*S*p**^ of scheme 1) by thin layer chromatography (TLC). The *R*_P_ and *S*_P_ diastereomers of the dimers 5′-asa, 5′-Afsa, 5′-usu 5′-asUf, 5′-usUf, and 5′-Ufsu except 5′-gsa were well separated on TLC and were isolated by flash column chromatography. The *R*_P_ and *S*_P_ isomers of 5′-gsa were separated by HPLC. Lowercase u, a, and g indicate 2′-OMe nucleoside, lowercase s indicates PS linkage, and Uf and Af indicate 2′-F U and A nucleosides, respectively. (**B**) The fully protected PS chirally pure *R*p and *S*p diastereomer phosphoramidites synthesized and isolated for incorporation into oligonucleotide under solid phase synthesis conditions to obtain chirally pure PS backbone containing siRNAs. From left to right and top to bottom, the compounds are 5′-usu (5a ^Rp^, 5b ^Sp^), 5′-usUf (11a ^Rp^, 11b ^Sp^), 5′-Ufsu (17a ^Rp^, 17b ^Sp^), 5′-asUf (23a ^Rp^, 23b ^Sp^), 5′-asa (29a ^Rp^, 29b ^Sp^), 5′-Afsa (35a ^Rp^, 35b ^Sp^) and 5′-gsa (41a ^Sp^, 41 ^Rp^).

We also tested coupling of the desired chiral PS dinucleotides as building blocks in oligonucleotide synthesis. The chiral PS dinucleotide phosphoramidite building blocks were successfully synthesized in three steps (Scheme 1). Phosphoramidite **I** and 5′-hydroxyl-3′-*O*-TBS-2′-modified (2′-*O*-Me or 2′-F) nucleoside **II** were reacted to generate PS dinucleotide diastereomeric mixture **III*^R^*^p^** and **III*^S^*^p^** in one pot. Compounds **I** and **II** were dissolved in DCM, activated, and coupled using ETT in acetonitrile under dry conditions. PADS in 2,6-lutidine was used for the subsequent sulfurization step. The separation of compound **III*^R^*^p^** from **III*^S^*^p^** was achieved by silica gel column chromatography (except for compound **39**, where the isomers could not be separated at this stage). Isomer separations by TLC are shown in Figure 3A. To remove the 3′-*O*-*tert*-butyldimethylsilyl (3′-*O*-TBS) protecting group, compounds **III*^R^*^p^** and **III*^S^*^p^** were treated with Et_3_N·3HF in THF at elevated temperature. Considering the sensitivity of the 2-cyanoethyl protecting group on the phosphorous to mild basic conditions, slightly acidic Et_3_N·3HF in THF was chosen to remove the TBS protection from the 3′-OH. The 4,4′-dimethoxytrityl protection at the 5′ end remained intact during the treatment with Et_3_N·3HF. The reaction mixture was concentrated and purified by silica gel column chromatography to yield pure **IV*^R^*^p^** and **IV*^S^*^p^** (in the case of compound **40**, the two isomers **40a** and **40b** were obtained after separation by RP-HPLC at this stage). In a final step, compounds **IV*^R^*^p^** and **IV*^S^*^p^** were reacted with the ETT-activated 2-cyanoethyl *N,N,N′,N′*-tetraisopropylphosphorodiamidite at 0°C to room temperature in DCM. Phosphitylation of **40a** using 2-cyanoethyl *N,N,N′,N′*-tetraisopropylphosphorodiamidite in the presence of slightly acidic ETT helped preserving the 2-cyanoethyl protecting group on the dimer. Subsequent quench, wash, concentration, and purification by column chromatography yielded the stereo-defined PS dinucleotide phosphoramidite building blocks **V*^R^*^p^** and **V*^S^*^p^** (Figure 3B). Neutralized silica gel columns were used for all purification as described in Materials and Methods. NMR spectra of these compounds can be found in Supplementary Information (Section I). The stereochemistry was assigned using two independent analytical methods: chemical shift in ^31^P-NMR (Ecksteins's rule) (57) and degradation studies on the deprotected dinucleotides by SVPD (57) and PDII. Results are shown in Table 2, and experimental details can be found in Supplementary Information Section II and III. Using this ‘dimer coupling approach’, we achieved >99% diastereomeric purity and good overall yield for all siRNAs (Supplementary Tables S4–S6).

**Scheme 2.**
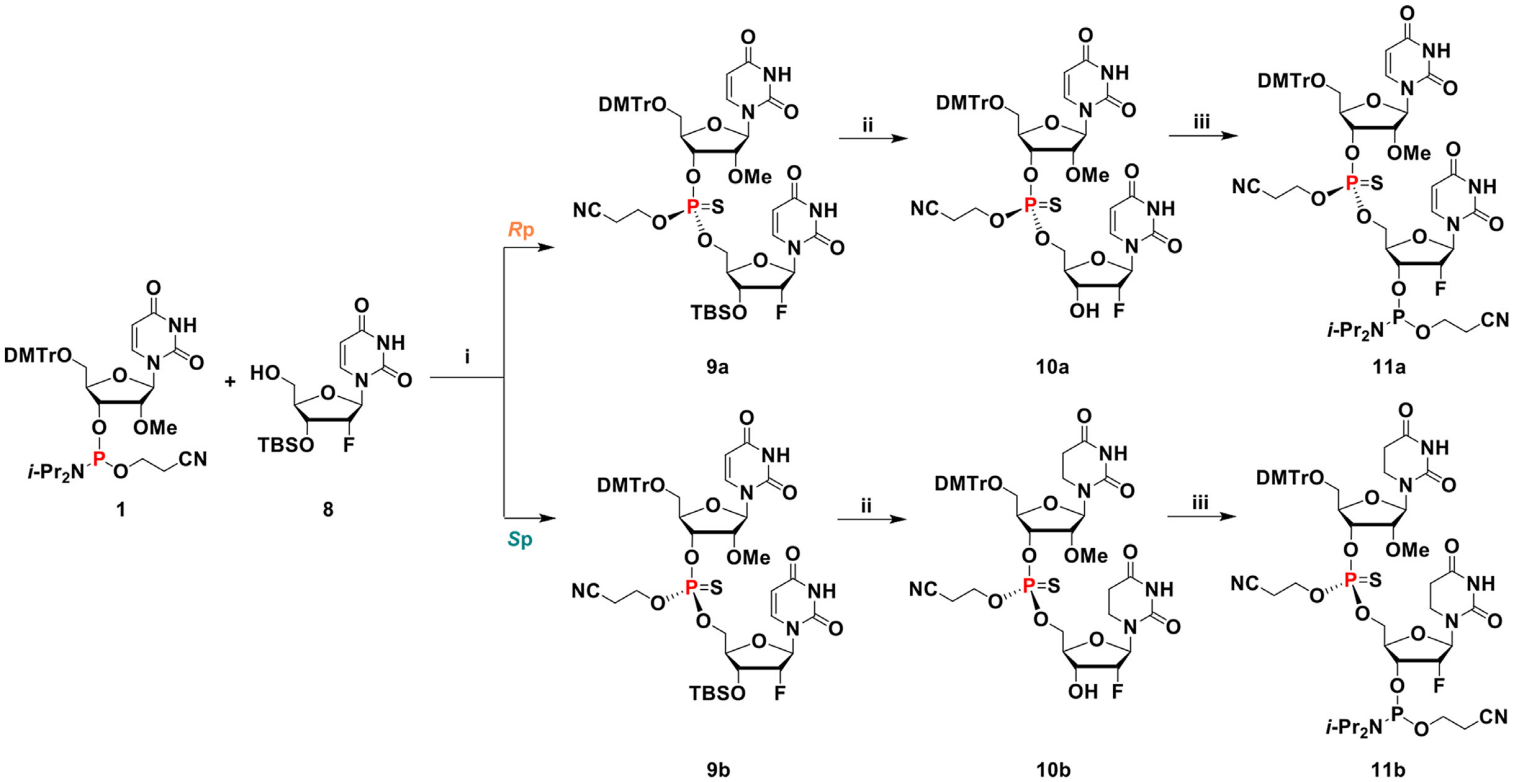
Synthesis procedure and characterization of stereo-defined dinucleotides 11a and 11b. (i) (a) ETT, CH_2_Cl_2_, room temperature, overnight; (b) PADS, 2,6-lutidine, room temperature, 4 h; (c) isomer separation, 46% (9a), 41% (9b); (ii) Et_3_N·3HF, THF, room temperature, 15 or 40 h, 76% (10a), 91% (10b); (iii) 2-cyanoethyl *N*,*N*,*N*′,*N*′-tetraisopropylphosphorodiamidite, ETT, CH_2_Cl_2_, 0°C to room temperature, 3 h or overnight, 74% (11a), 76% (11b).

**Table 2.** Properties of fully deprotected PS dinucleotide diastereomers

		*Rp* ^a^				*Sp* ^b^			
Dimer	HRMS calc.	HRMS found	^31^P NMR^e^	SVPD^f^	PDII^g^	HRMS found	^31^P NMR^e^	SVPD^f^	PDII^g^
*usu*	617.09^c^	617.09^c^	56.74	deg.	-	617.09^c^	55.55	no deg.	-
*usUf*	605.07^c^	605.07^c^	56.78	deg.	-	605.07^c^	55.68	no deg.	-
*Ufsu*	605.07^c^	605.07^c^	56.82	deg.	no deg.	605.07^c^	55.97	no deg.	deg.
*asa*	641.17^d^	641.17^d^	57.51	deg.	no deg.	641.17^d^	56.27	no deg.	deg.
*Afsa*	629.15^d^	629.15^d^	57.21	deg.	no deg.	629.15^d^	55.40	no deg.	deg.
*asUf*	606.12^d^	606.12^d^	57.83	deg.	no deg.	606.12^d^	56.63	no deg.	deg.
*gsa*	657.16^d^	657.16^d^	56.07	no deg.	deg.	657.16^d^	57.53	deg.	no deg.

^a^The *R*p diastereomer was the top spot on TLC for all dinucleotides except for gsa.

^b^The *S*p diastereomer was the lower spot on TLC for all dinucleotides except gsa.

^c^HRMS, high-resolution mass spectrometry: (M+Na).

^d^HRMS: (M+H).

^e^Chemical shift (ppm) in ^31^P NMR spectrum of fully deprotected dinucleotide in D_2_O.

^f^SVPD degradation at 48 h time point.

^g^PDII degradation at 48 h time point. deg. indicates that there was degradation; no deg. indicates no degradation.

Lowercase u, a, and g indicate 2′-OMe nucleosides, lowercase s indicates PS linkage, and Uf and Af indicate 2′-F U and A nucleosides, respectively.

Both methods proved effective in preparing chiral PS oligonucleotides for physico-chemical and biological studies. The two approaches resulted in similar synthetic yields of stereo-defined PS oligonucleotides; however, the coupling approach yielded higher diastereomeric purity on a larger scale than the purification approach. Since the dimer approach provided well-defined 100% stereo-purity, the oligonucleotides synthesized using the dimer approach were used for both *in vitro* and *in vivo* studies discussed below (Supplementary Tables S4 and S7 and Figure S21).

### Defined PS stereochemistry can enhance *in vitro* activity of siRNAs

**Figure 4.**
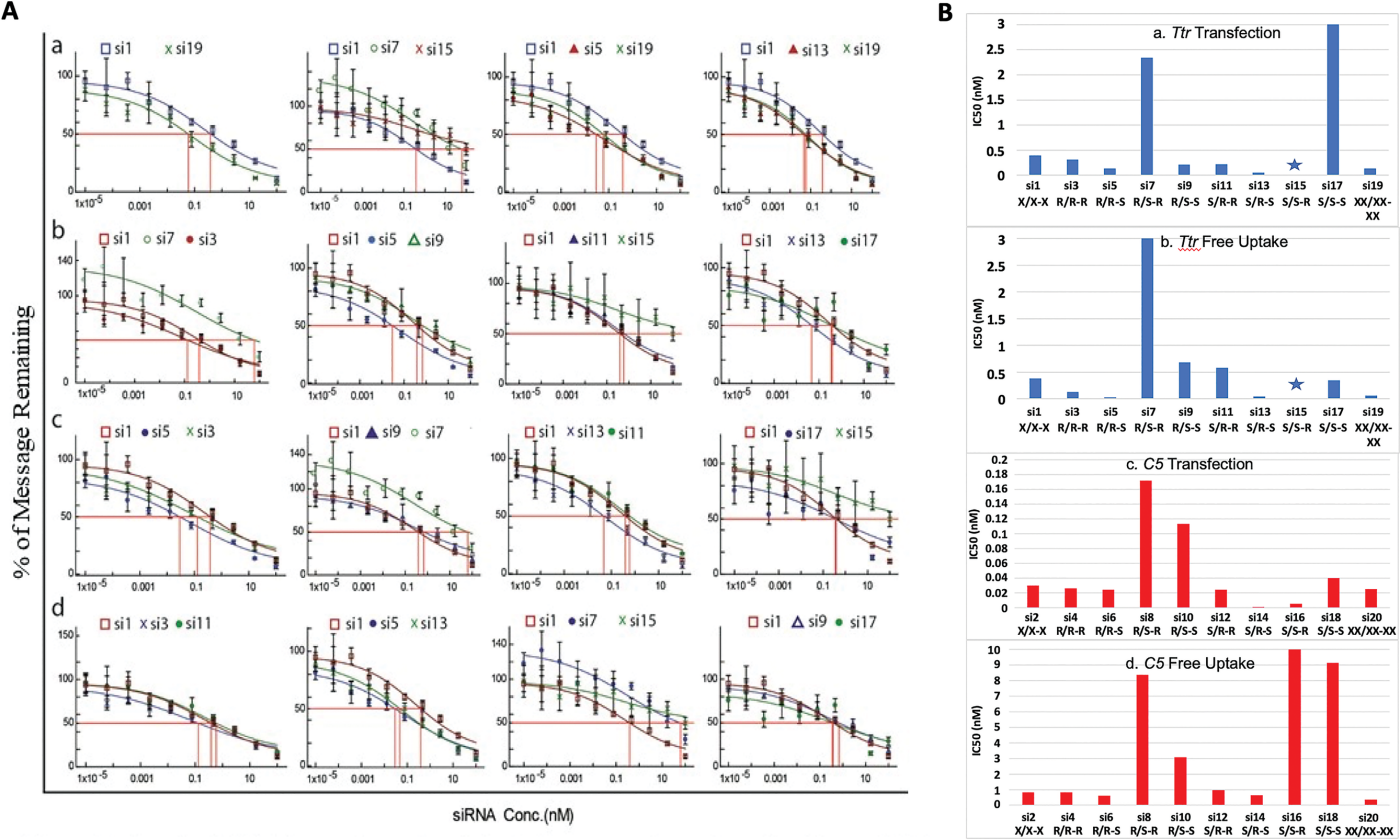
(**A**) *In vitro* evaluation of gene silencing in primary mouse hepatocytes incubated for 24 h with *Ttr*-targeted siRNAs under free-uptake conditions. a) Percent *Ttr* mRNA remaining quantified by RT-qPCR as a function of siRNA concentration for least (R/S-R, si7; S/S-R, si15) and most active (R/R-S, si5; S/R-S, si13) siRNAs relative to stereo-random parents (X/X-X, si1; XX/XX-XX, si19). b) Percent mRNA remaining as a function of siRNA concentration for siRNAs with a single isomer change at the 5′ end of the antisense strand (R/S-R, si7, R/R-R, si3; R/R-S, si5; R/S-S, si9; S/R-R, si11; S/S-R, si15; S/R-S, si13; S/S-S, si17) relative to the stereo-random parent (X/X-X, si1). c) Comparison of duplexes with a single isomer change at the 3′ end of the antisense strand (R/R-S, si5; R/R-R, si3; R/S-S, si9; R/S-R, si7; S/R-S, si13; S/R-R, si11; S/S-S, si17; S/S-R, si15) relative to the stereo-random parent (X/X-X, si1). d) Percent mRNA remaining as a function of siRNA concentration for siRNAs with a single isomer change at the 5′ end of the sense strand (R/R-R, si3; S/R-R, si11; R/R-S, si5; S/R-S, si13; R/S-R, si7; S/S-R, si15; R/S-S, si9; S/S-S, si17) relative to stereo-random parent (X/X-X, si1). (**B**) IC_50_ values derived from above experiments for a) *Ttr* mRNA under transfection conditions, b) *Ttr* mRNA under free uptake conditions, c) *C5* mRNA under transfection conditions and d) *C5* mRNA under free uptake conditions. The star indicates values that could not be ascertained.

To assess the biological impact of different PS diastereomers at the terminal positions of siRNAs, we measured silencing of *Ttr* and *C5* expression in primary mouse hepatocytes treated with stereo-random and stereo-defined PS siRNAs. Cells were treated with siRNAs using a transfection reagent or *via* free uptake; the latter occurs through the asialoglycoprotein receptor (ASGPR) due to the GalNAc ligand conjugated to the siRNA. mRNA levels were quantified by qPCR. The dose-response relationships of stereo-defined PS siRNAs on *Ttr* mRNA levels are shown in Figure 4A. Data are summarized in Table 1 and Figure 4B for both *Ttr* and *C5*. As expected, the siRNAs with three stereo-random PS linkages (S/AS: 5′X/5′X-3′X, si1 and si2) were slightly less active than the siRNAs with six stereo-random PS linkages (S/AS: 5′XX/5′XX-3′XX, si19 and si20). Surprisingly, the siRNAs with the R/S-R (si7 and si8) and S/S-R (si15 and si16) stereo configurations were significantly less active than the siRNAs with three stereo-random PS linkages in cells treated by free uptake. In contrast, the siRNAs with R/R-S (si5 and si6) and S/R-S (si13 and si14) chiral PS configurations reduced *Ttr* and *C5* mRNA considerably more than the stereo random X/X-X (si1 and si2) siRNAs and to a similar degree as the siRNAs with six stereo-random PS linkages (si19 and si20) (Figure 4Aa).

When we compared siRNAs with only a single isomer difference, those with an *R*p isomer at the 5′ end of the antisense strand demonstrated greater *in vitro* activity in all cases (Figure 4Ab). For example, the R/**R**-R siRNAs (si3 and si4) were more potent than the R/**S**-R (si7 and si8) counterparts. The same was true for the other pairs (R/**R**-S, si5/si6 > R/**S**-S, si9/si10; S/**R**-R, si11/si12 > S/**S**-R, si15/si16; and S/**R**-S, si13/si14 > S/**S**-S, si17/si18). An *S*p isomer at the 3′ end of the antisense strand also improved the siRNA activity irrespective of the configurations of the other two PS linkages (R/R-**S**, si5/si6 > R/R-**R**, si3/si4; R/S-**S**, si9/si10 > R/S-**R**, si7/si8; S/R-**S**, si13/si14 > S/R-**R**, si11/si12; and S/S-**S**, si17/si18 > S/S-**R**, si15, si16) (Figure 4Ac). In contrast, the PS absolute configuration on the sense strand did not show a clear trend with regards to its influence on the *in vitro* activity for the siRNAs evaluated in this study (Figure 4Ad).

### siRNAs with unique PS stereochemistries confer increased potency *in vivo*

**Figure 5.**
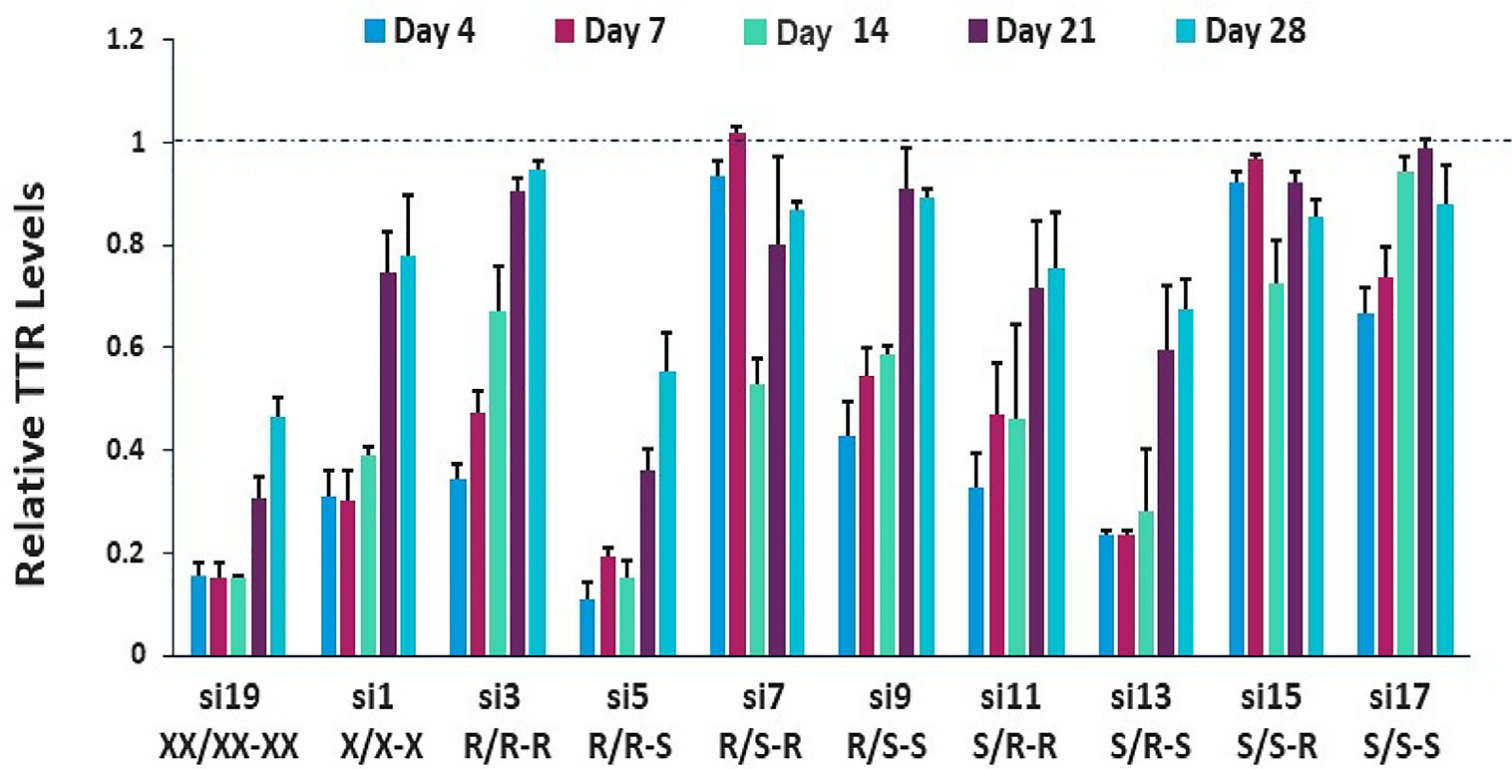
*In vivo* evaluation of *Ttr*-targeting siRNAs containing terminal stereo-defined PS linkages in comparison to the stereo-random controls. Female C57BL/6 mice (*n* = 3) received a single, 2 mg/kg subcutaneous dose of the indicated *Ttr*-targeted siRNA. Circulating TTR levels were measured by ELISA on days 4, 7, 14, 21 and 28 post dose with each sample normalized to the pre-dose levels for the individual animal.

**Figure 6.**
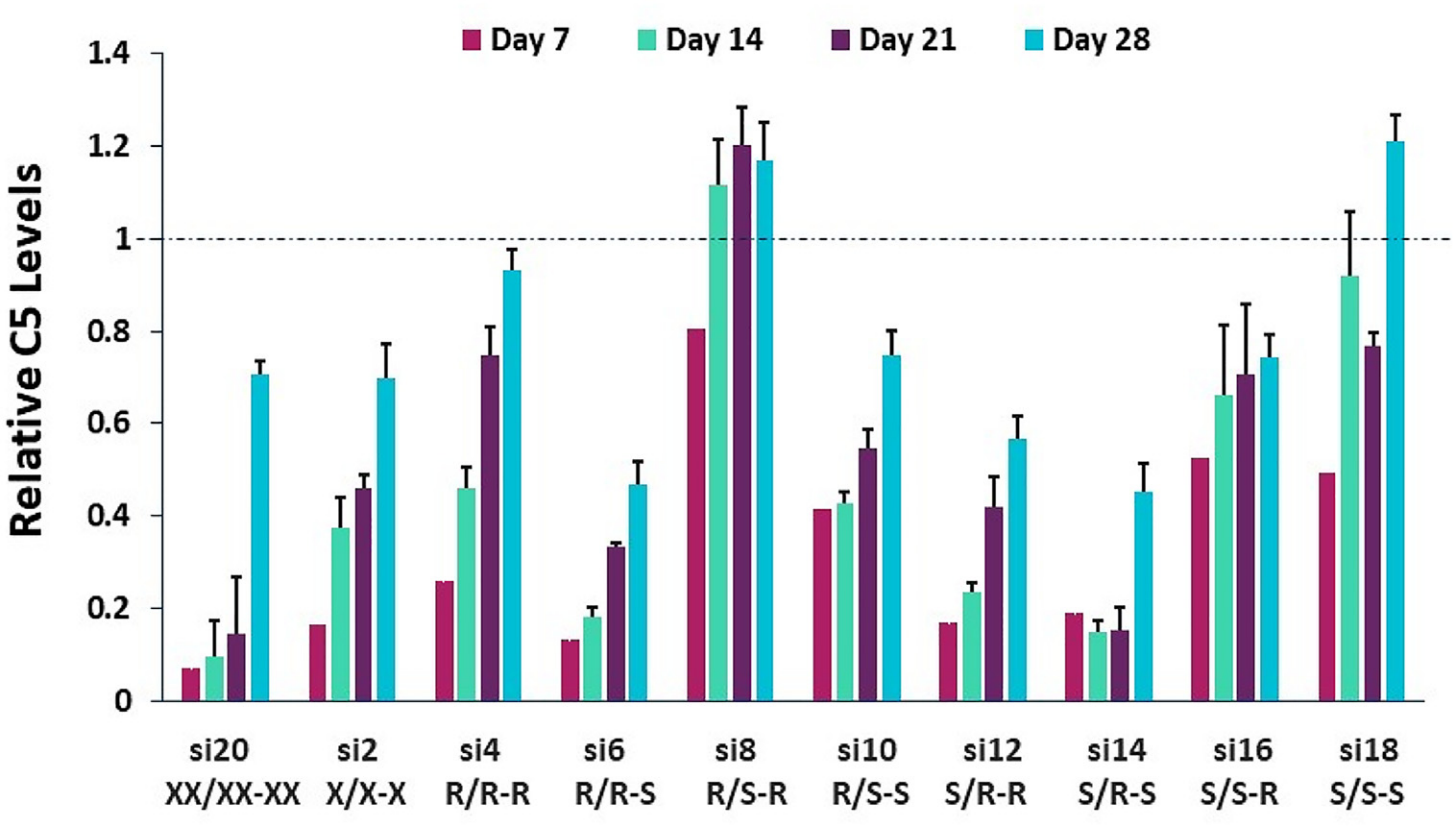
*In vivo* evaluation of *C5-*targeting siRNAs containing stereo-defined PS linkages at the termini compared to stereo-random controls. Female C57BL/6 mice (*n* = 3) received a single, 5 mg/kg subcutaneous dose of the indicated *C5*-targeted siRNA. Circulating C5 levels were measured by ELISA on days 7, 14, 21 and 28 post dose with each sample normalized to the pre-dose levels for the individual animal.

Based on the *in vitro* activity data, the most promising siRNA PS configuration was R/R-S followed by S/R-S, whereas both R/S-R and S/S-R were detrimental. To confirm these *in vitro* potency trends *in vivo*, we evaluated potencies of *Ttr*- and *C5*-targeted stereo-defined GalNAc-siRNAs in mice. In addition to the three stereo-random PS siRNAs (si1 and si2), the stereo-defined siRNAs were also compared to the siRNAs with six stereo-random PS linkages (si19 and si20). Single subcutaneous doses of 2 mg/kg of stereo-random si19 and si1 siRNAs reduced circulating TTR protein levels by approximately 85% and 70%, respectively (Figure 5). Similarly, a single subcutaneous dose of 5 mg/kg of si20 and si2 siRNA reduced circulating C5 protein by about 93% and 84% at nadir, respectively (Figure 6). The *in vivo* activity confirmed the trends observed in the primary mouse hepatocytes. Specifically, the siRNAs with an *S*p isomer at the 5′ end of the antisense strand (R/**S**-R, si7 and si8; R/**S**-S, si9 and si10; S/**S**-R, si15 and si16; and S/**S**-S, si17 and si18) were significantly less potent than the siRNAs with three stereo-random PS linkages (si1, si2), whereas the siRNAs with an *R*p isomer at this position (R/**R**-S, si5 and si6; and S/**R**-S, si13 and si14) had efficacies comparable to the siRNAs with six stereo-random PS linkages (si19, si20) and significantly higher than those with three stereo-random PS linkages (si1 and si2).

**Figure 7.**
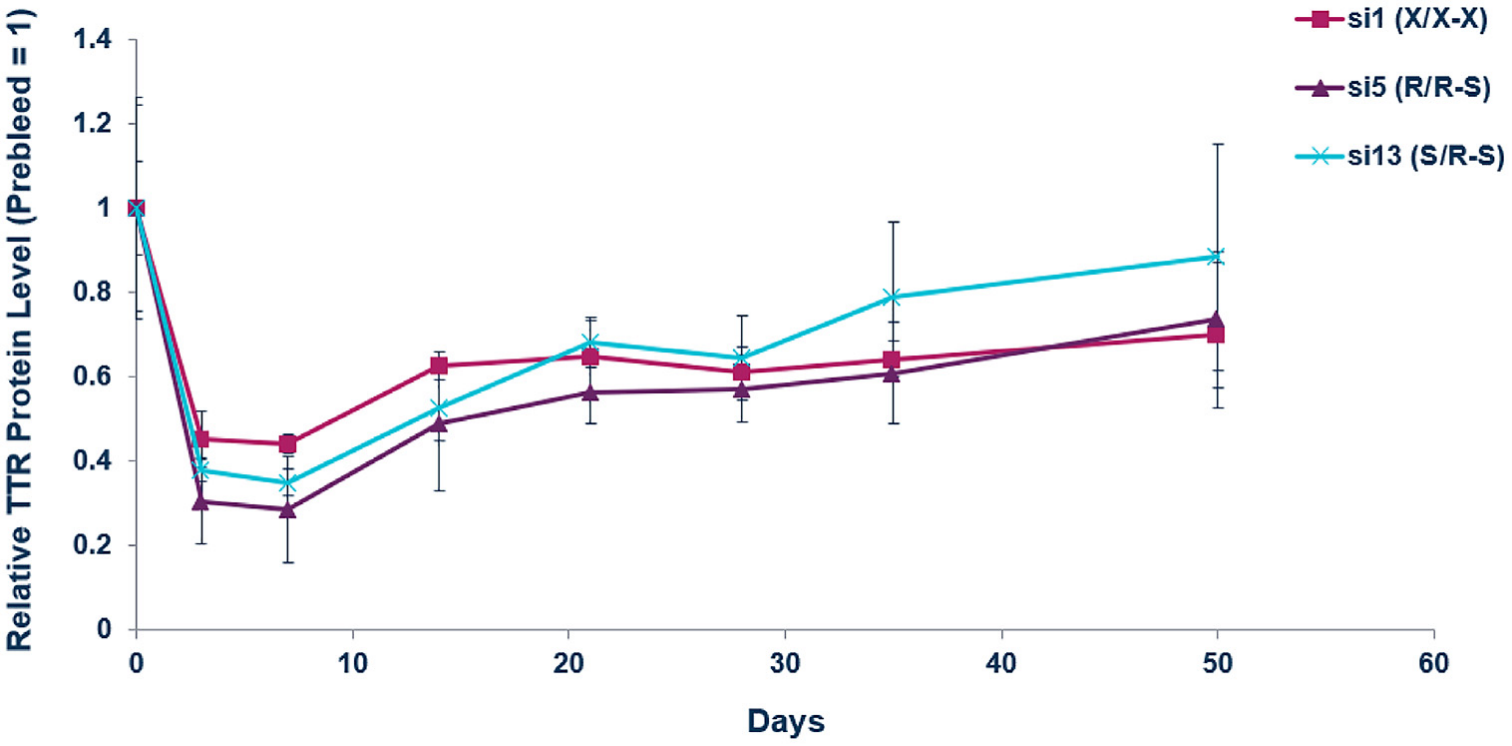
*In vivo* evaluation of gene silencing with siRNAs showing the importance of an *R*p isomer at the 5′ end of the antisense strand confirmed in rats. Female Sprague-Dawley rats (*n* = 3) were given a single, 2.5 mg/kg subcutaneous dose of indicated *Ttr*-targeted siRNA. Circulating TTR protein levels were measured by ELISA over a 50-day period post dose with each sample normalized to the pre-dose levels for the individual animal.

We next evaluated the two most potent *Ttr*-targeted siRNAs, both with an *R*p isomer at the 5′ end of the antisense strand (R/**R**-S, si5; and S/**R**-S, si13), in the rat. Oligonucleotides are usually metabolized more rapidly in rats than in mice; therefore, the impact of metabolic stability on siRNA potency is more pronounced in the rat. In this study, female Sprague-Dawley rats were administered a single, 2.5 mg/kg subcutaneous dose of siRNA, and the circulating TTR protein levels were measured over a 50-day period. Similar to the results in mice, the R/R-S (si5) and S/R-S (si13) *Ttr*-targeted siRNAs were more active than the stereo-random siRNA (si1). The maximum circulating TTR reduction for si1 was 56%, whereas that for si5 was 71%; si13 showed intermediate potency (Figure 7).

### Metabolic stability of stereo-defined siRNAs

**Figure 8.**
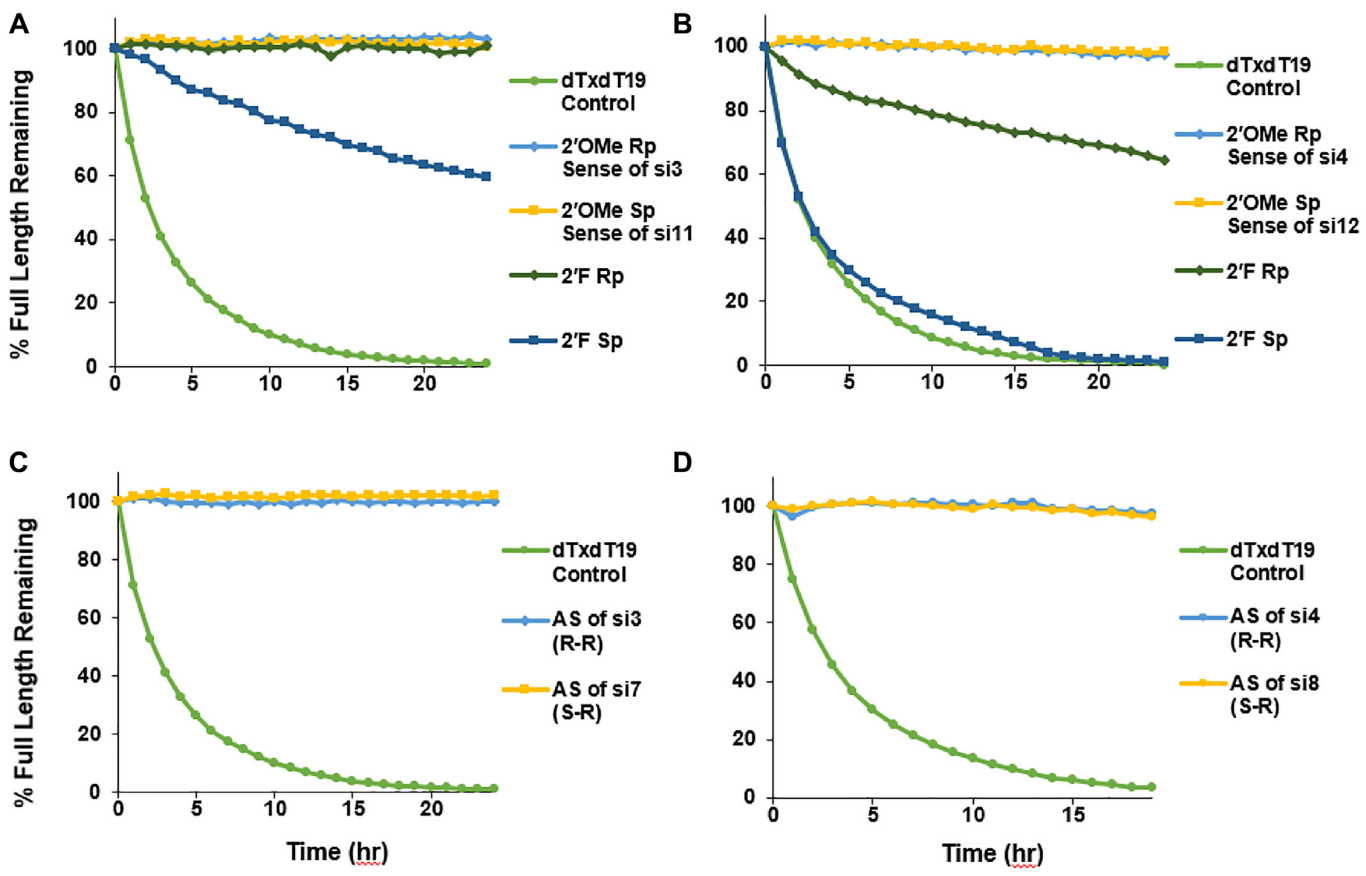
*R*p configuration confers greater stability than *S*p in the presence of the 5′ exonuclease PDII. Percent full-length strand for (**A**) *Ttr* sense, (**B**) *C5* siRNA sense, (**C**) *Ttr* antisense, and (**D**) *C5* antisense strands incubated with 5′ exonuclease PDII as a function of time. In each experiment, the control dT_20_ with a 5′ terminal stereo-random PS (dTxdT19) was tested.

The *in vitro* and *in vivo* activity data demonstrated that the stereochemistry of the 5′ and 3′ terminal PS linkages influence siRNA potency. To determine if this is due to effects on metabolic stability, we performed exonuclease assays on the single strands of *Ttr*- and *C5*-targeted siRNAs. The oligonucleotides were incubated with 5′ exonuclease PDII or 3′ exonuclease SVPD, and percent full-length product was monitored over time by UV-HPLC (58). When incubated with PDII under these conditions, no degradation of the sense strand of the *Ttr*-targeted siRNA was detected irrespective of the configuration of the 5′ end PS linkage (*R*p sense strand of si3/si5/si7/si9 or *S*p sense strand of si11/si13/si15/si17; Figure 8A). However, when the 5′ terminal nucleotide of the *Ttr*-targeted siRNA sense strand was changed from 2′-OMe adenosine to 2′-F adenosine, a substantial reduction in stability in the presence of PDII was observed, with the *R*p configuration of the PS demonstrating greater stability than *S*p. The same was true for the *C5*-targeted sense strands of si4/si6/si8/si10 or si12/si14/si16/si18 when the 5′ terminal nucleotide of the sense strand was changed from 2′-OMe guanosine to 2′-F guanosine (Figure 8B). When the *Ttr* and *C5* antisense strands had 5′ terminal PS linkages and 2′-OMe nucleotides, no degradation was observed in the presence of PDII irrespective of the PS configuration on the 5′ end (Figure 8C and D). Thus, 2′-F is needed at 5′ terminus to see the effect of chirality on the nuclease activity.

**Figure 9.**
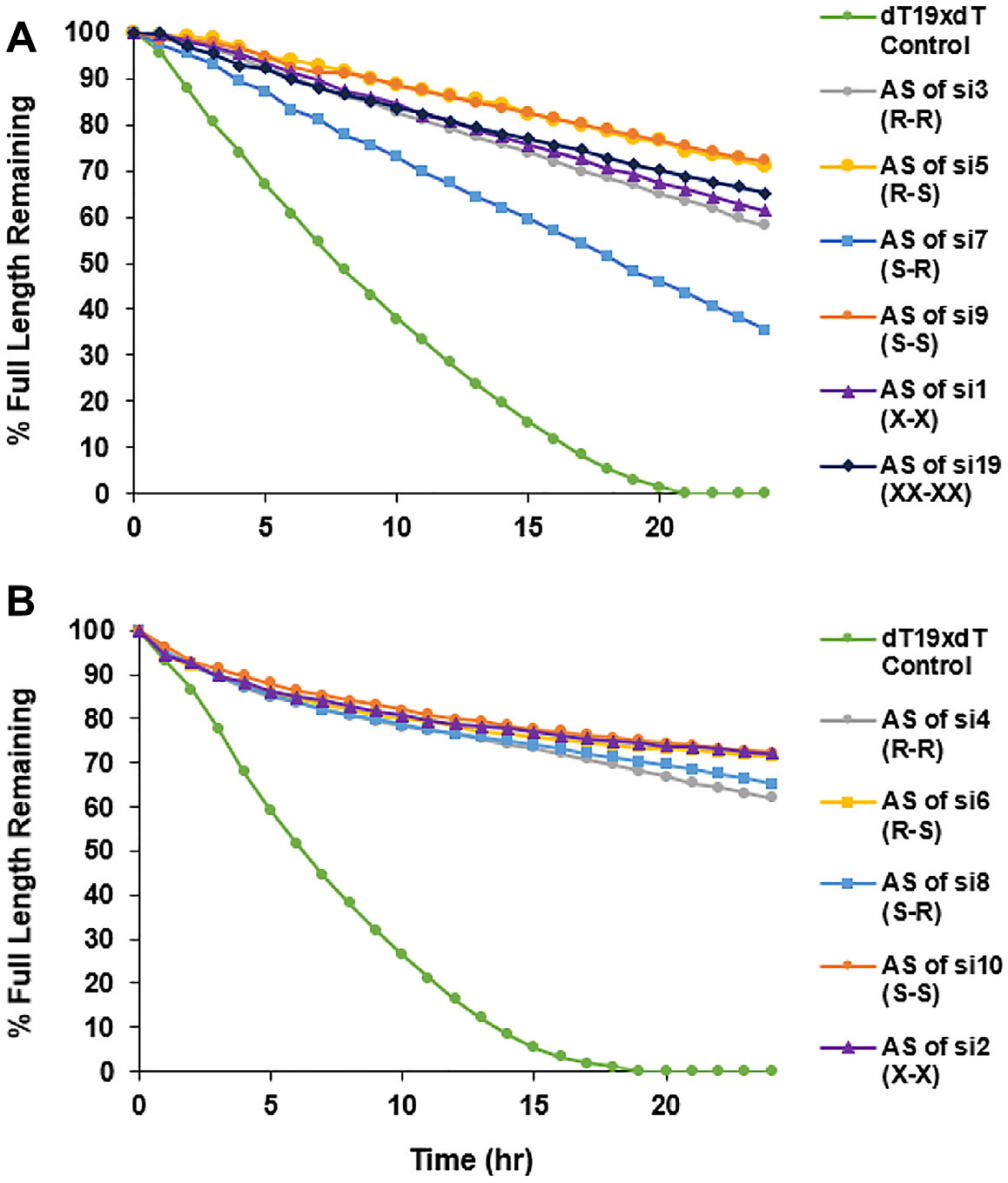
*S*p isomer enhances stability in the presence of 3′ exonuclease. Percent full-length (**A**) *Ttr* and (**B**) *C5* siRNA antisense strands when incubated with 3′ exonuclease SVPD over 24 h.

The antisense strands of the *Ttr*-targeted siRNAs were susceptible to degradation by the 3′ exonuclease SVPD in a manner that was dependent on the 3′ PS stereoisomer (Figure 9A). The half-lives of the antisense strands of si5/si13 (R–S configuration: 47 h) and of si9/si17 (S–S configuration: 50 h) were longer than that of si1 with single stereo-random PS linkages (33 h) and of si19, which has two stereo-random PS linkages on each terminus (39 h). The antisense strand of si7/si15, with the S–R configuration, on the other hand, had a significantly shorter half-life (16 h). Surprisingly, the half-life of the antisense strand with the R-R configuration in si3/si11 (30 h) was longer than that of the antisense strand with the S–R configuration in si7/si15, but shorter than that of antisense strand of si1 with stereo-random linkages on each end (Figure 9A). Although less pronounced, similar results were observed when the antisense strands of the *C5*-targeted siRNAs were analyzed (Figure 9B). In this case, the half-life of the antisense strand in si2 with the isomer mixture had a half-life (59 h) equivalent to those of the antisense strands of si6/si14 (R–S: configuration: 56 h) and si10/si18 (S–S configuration: 57 h), whereas the antisense strands of si4/si12 (R–R: configuration: 39 h) and those of si8/si16 (S–R configuration: 45 h) had slightly shorter half-lives. The increased stability of the *S*p isomer toward 3′ exonuclease is in agreement with previously published data (59).

**Figure 10.**
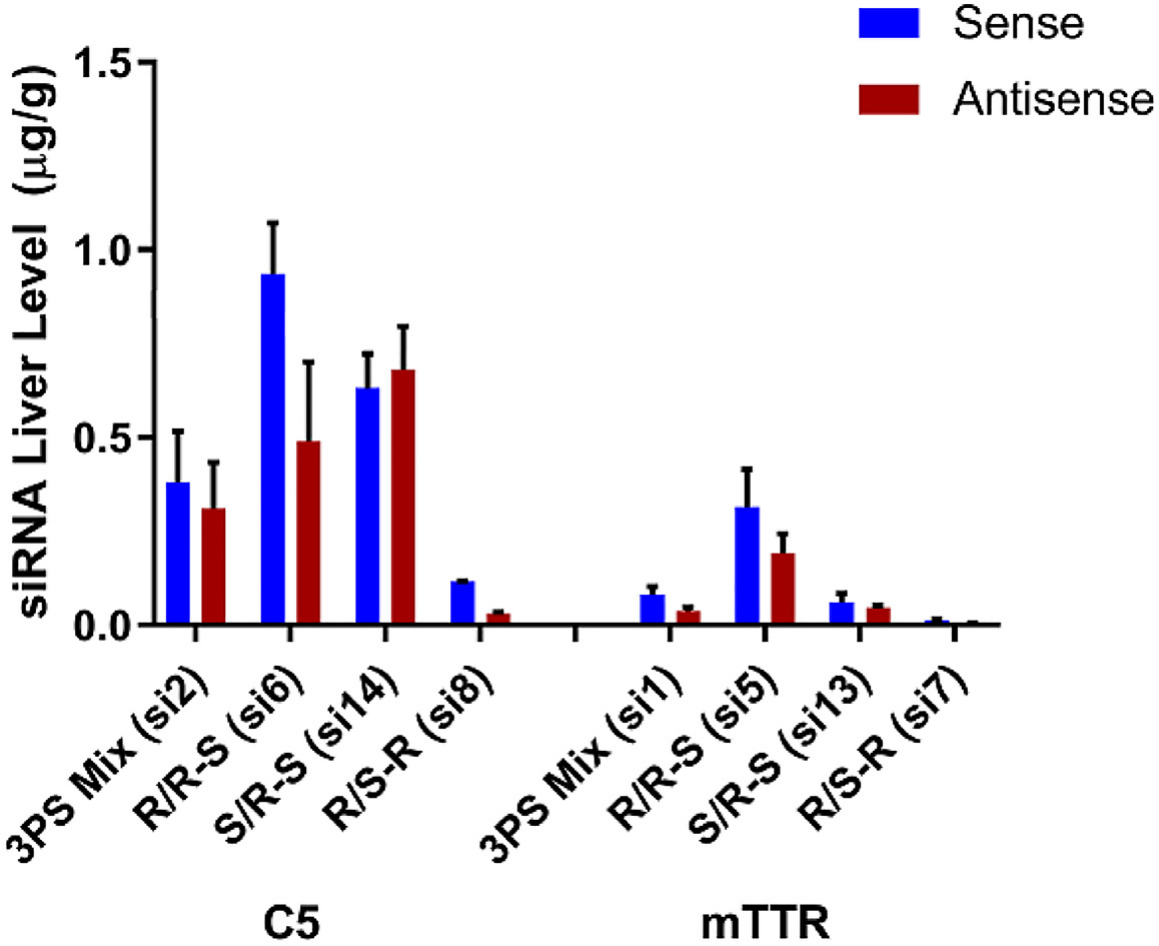
*In vivo* metabolic stability of siRNA is dependent on stereochemistry of terminal PS linkages. Mice (*n* = 3) were treated with single subcutaneous injections of 2.5 mg/kg *C5*-targeted and 2 mg/kg for *Ttr*-targeted siRNAs. After 5 and 7 days, respectively, liver levels of sense and antisense siRNA were quantified by stem-loop RT-qPCR.

To further confirm that the *S*p stereoisomer at the 3′ end of the antisense strand imparts nuclease stability *in vivo*, we examined the levels of sense and antisense strands in whole liver homogenates at day 7 for the *Ttr*-targeted siRNA-treated mice and at day 5 from the *C5*-targeted siRNA-treated animals. Compared to the siRNAs with three stereo-random PS linkages (si1 and si2), higher levels of the R/R–S (si5 and si6) siRNA sense and antisense strands were observed (Figure 10). In contrast, very little of the siRNAs with the opposite PS configurations of the antisense strand (R/S–R, si7 and si8) were detectable (Figure 10). Taken together, these data show that the *S*p stereochemistry at the 3′ end of the antisense strand enhances metabolic stability.

### Molecular modeling suggests that Ago2 interaction influences stereoisomer potencies

A plausible explanation for the differences in therapeutic potency of the stereo-defined siRNAs is efficacy of loading into RISC. In RISC, the siRNA interacts with Ago2. Crystal structures of human Ago2 in complex with siRNA antisense strands show extensive hydrogen-bonding contacts between Ago2 residues and the non-bridging oxygens of the phosphodiester linkages at the 3′ and 5′ ends of the antisense strand (60,61). It therefore stands to reason that the stereochemistry of PS linkages at these positions could influence loading efficiency. To test this, we looked at the binding features of the chiral phosphorothioates in the context of the established crystal structures of Ago2.

**Figure 11.**
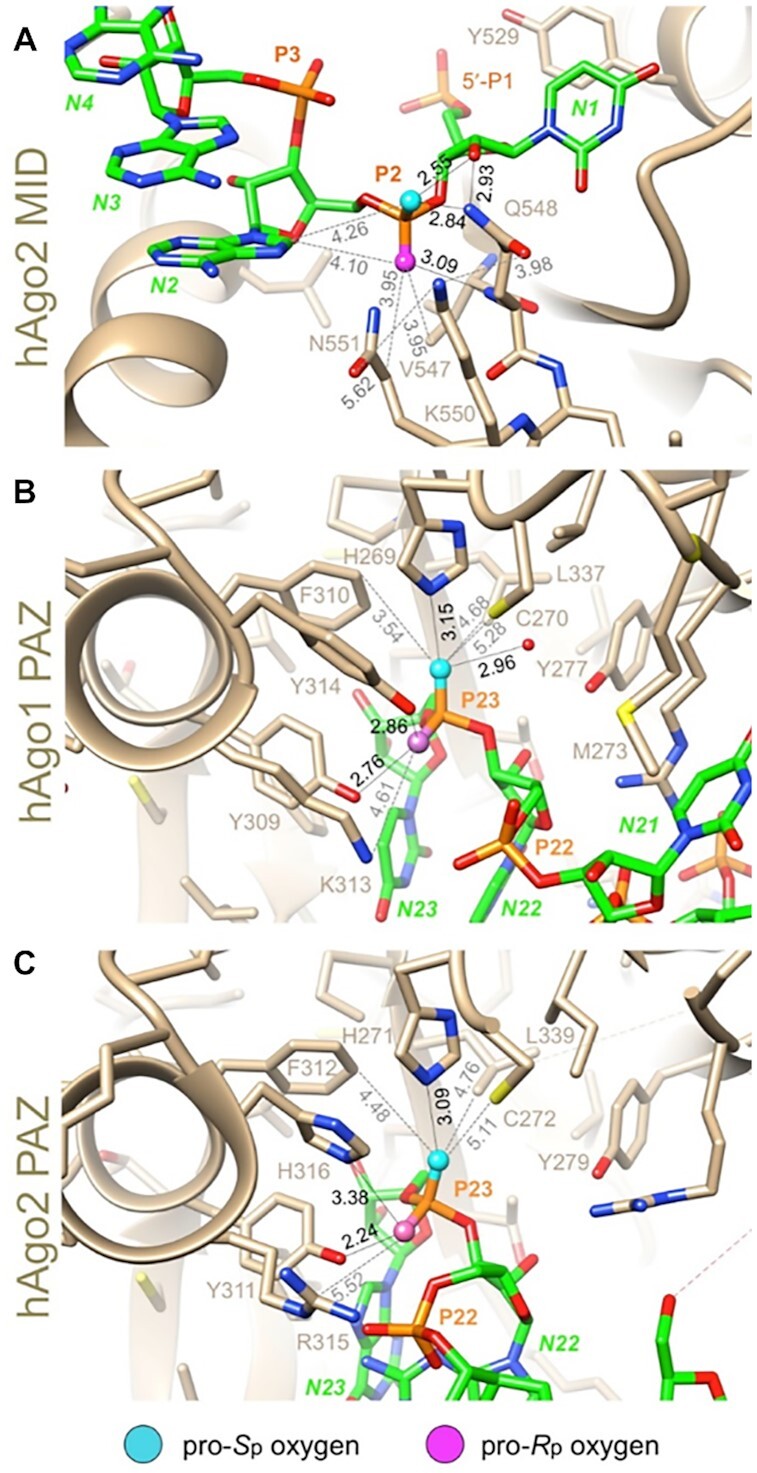
Environments of the 5′- and 3′-terminal phosphate groups of the antisense siRNA strand bound to Ago proteins. (**A**) The region of the 5′ terminus of the antisense strand bound to the MID domain of human Ago2 (PDB ID: 4F3T). P1 is the 5′ phosphate; P2 is the first bridging phosphate. (**B**) The region of the 3′-terminal end of the antisense strand bound to the PAZ domain of human Ago1 (PDB ID: 1SI3). P23 is the terminal linkage. (**C**) The region of the 3′-terminal end of the antisense strand bound to the PAZ domain of human Ago2 (PDB ID: 4F3T). P23 is the terminal linkage. Selected residues are labeled. Hydrogen bonds are drawn with thin solid lines, and further contacts are indicated with dashed lines with distances in Å. The illustrations were generated with the program UCSF Chimera (63).

In the crystal structure of the complex between Ago2 and miR20a (60) the 5′-terminal phosphate is tightly bound by MID domain residues, and the strand makes a sharp turn between the first and second nucleotides (Figure 11A). The pro-*S*p oxygen of the first bridging phosphate is an acceptor in two hydrogen bonds, one with the 2′-OH of N1 and the other with the amino group of the Q548 side chain (Figure 11A). The pro-*R*p oxygen makes a single hydrogen bond to the main chain amino group of Q548. It is otherwise unrestrained, and neighboring atoms (adenine C8-H of N2 and methylene and methyl groups from N551 and V547, respectively) all lie at around 4 Å from this oxygen (Figure 11A). Thus, steric effects combined with the hydrophobicity of adjacent residues in the MID domain can rationalize the higher activity of siRNAs with an *R*p than the *S*p isomer at the first bridging phosphate of the antisense strand.

The 3′ end of the antisense strand is anchored in the PAZ domain, a highly conserved largely hydrophobic RNA-binding domain found in Ago proteins. We examined the crystal structures of the human Ago1 PAZ bound to an RNA 9-mer (62) and of human Ago2 bound to miR20a (60). In both structures, the environments of the pro-*R*p and pro-*S*p phosphate oxygens of the 3′-terminal phosphate group differ considerably. In the complex with the Ago1 PAZ domain, the pro-*R*p oxygen forms hydrogen bonds with the side chains of two tyrosines (Y309 and Y314) and the Nζ of K313 lies nearby (Figure 11B). Thus, the 3′-most residue of the antisense strand is located in a relatively polar environment. By comparison, the pro-*S*p oxygen is engaged in hydrogen bonds to H269 and water, but additional neighboring residues (C270, F310, and L337) are all hydrophobic in nature, and they lie at distances that exceed the sum of the van der Waals radii (Figure 11B). In the complex with Ago2, H316 is hydrogen bonded to the pro-*R*p oxygen and R315 lies nearby. The environments of the pro-*S*p oxygen in the Ago1 and Ago2 complexes are virtually identical (Figure 11C). Thus, oxygen in the pro-*R*p orientation at the 3′ terminus of the antisense strand is probably preferred relative to sulfur as its more electronegative nature will result in stronger binding. Therefore, based on steric and stereo-electronic considerations, we conclude that an *S*p PS diastereomer at the 3′ end of the antisense strand will likely increase Ago2 loading relative to the *R*p isomer.

### *S*p PS configuration at the 3′ end of the antisense strand facilitates Ago2 loading *in vitro* and *in vivo*

**Figure 12.**
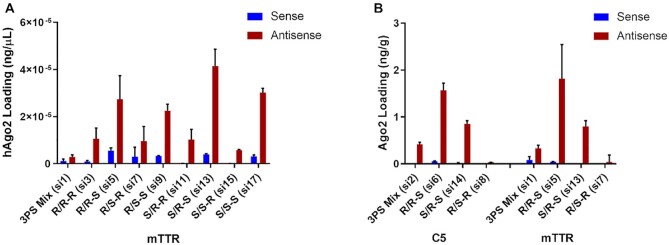
The *S*p stereoisomer at the 3′ end of the antisense strand is beneficial for Ago2 loading. (**A**) HEK293 cells overexpressing FLAG-HA-tagged human Ago2 were lysed, and lysates were incubated with 200 nM of the indicated siRNAs for 4 h before immunoprecipitation of the Ago2 using an anti-HA antibody. Ago2-associated siRNAs were eluted by heating and quantified by stem-loop RT-qPCR. (**B**) Whole liver homogenates were prepared from C57BL/6 female mice (*n* = 3) treated with single subcutaneous injections of the either 2.5 mg/kg *C5*-targeted siRNA (sacrificed on day 5 post-dose) or 2 mg/kg *Ttr*-targeted siRNA (sacrificed on day 7 post-dose) Ago2-associated siRNAs were eluted by heating and quantified by stem-loop RT-qPCR.

To experimentally interrogate the interaction between a stereo-defined antisense siRNA strand and the RISC protein complex, we quantified Ago2-associated siRNA after addition of siRNAs to lysates of HEK293 cells that overexpress human Ago2 (Figure 12A). The siRNAs with an *S*p isomer at the 3′ end of the antisense strands were more efficiently associated with Ago2 than those with an *R*p isomer (R/R–S si5, si6 > R/R–R si3, si4; R/S–S si9, si10 > R/S–R si7, si8; S/R–S si13, si14 > S/R–R si11, si12; and S/S–S si17, si18 > S/S–R si15, si16). On the other hand, the PS configuration at the 5′ end of the antisense strand played a minor role in affinity for Ago2 (R/R–R si3, si4 = R/S–R si7, si8; R/R–S si5, si6 ∼ R/S–S si9, si10; S/R–R si11, si12 > S/S–R si15, si16; and S/R–S si7, si8 > S/S–S si17, si18). The impact of stereochemistry at the 5′ end of the sense strand on Ago2 loading was negligible. Two siRNAs, those with the R/R–S (si5) and S/R–S (si13) configurations, showed greater than ten-fold higher antisense strand loading compared to stereo-random siRNA (si1; Figure 12A). Thus, as suggested from the molecular modeling studies, an *S*p stereoisomer at the 3′ end of the antisense strand can improve Ago2 loading.

To further confirm that the *S*p stereoisomer at the 3′ end of the antisense strand is beneficial for Ago2 loading, we examined the levels of sense and antisense strand associated with Ago2 in total liver homogenates from the mice treated with *C5*- or *Ttr*-targeted, stereo-defined siRNAs at day 5 and 7, respectively. Approximately 3-fold more of the antisense strands of the siRNAs with the stereochemistry R/R–S (si5, si6) were associated with Ago2 than those with the stereo-random PS linkages (si1, si2) siRNAs (Figure 12B). In addition to the differences in RISC loading, metabolic stability also impacts levels of RISC loading *in vivo* (Figure 12B).

## DISCUSSION

Except at the 3′ end of the sense strand, which carries the GalNAc ligand, all currently approved GalNAc-conjugated RNAi therapeutics have two PS linkages at the termini of each strand to provide protection against nuclease activity *in vivo* (7,43–47). Here we systematically studied the properties of a simplified design containing single stereo-defined terminal PS linkages. We tested two approaches for synthesis of these siRNAs. The first was a purification approach in which we separated the single-stranded oligonucleotides containing the desired PS isomers by ion exchange chromatography after stereo-random synthesis and before annealing into double-stranded siRNAs. The second approach involved coupling of the stereo-defined PS dinucleotide building blocks during oligonucleotide synthesis. The dinucleotide coupling approach yielded higher diastereomeric purity and improved scalability compared to the purification approach. Based on this finding, we used the latter method to synthesis siRNAs used to evaluate *in vitro* and *in vivo* differences between chirally defined siRNAs. Some of the recently reported methods for the stereo-controlled synthesis of PS oligonucleotides have limitations including cost of the novel chiral-auxiliary phosphoramidites, low coupling yields, and requirement for harsh conditions for the sulfurization of P(III) to P(V) and deprotection steps. The synthetic approaches described in this work use standard conditions and reagents for oligonucleotide synthesis and are compatible with the myriad of chemical modifications required for potential applications in oligonucleotide therapeutics. Using the chiral dinucleotides synthetic approach, we achieved very high coupling efficiency and 99% diastereomeric purity, allowing us to synthesize stereo-defined PS siRNAs in high yields. We have also demonstrated the scalability of the dimer approach for various dimers.

The stereo-defined siRNAs were evaluated *in vitro* and *in vivo* and compared to their stereo-random counterparts as controls. Our results indicate that the *S*p configuration at the 3′ end of the antisense strand improves efficacy of siRNA-induced gene silencing compared to the control with three stereo-random PS linkages due to a combination of enhanced metabolic stability and more efficient Ago2 loading. Many additional factors may affect the potency of siRNAs including strand selection (64,65), the presence of nucleotide motifs (66), chemical modification of the antisense and/or sense strands (67), and duplex thermal stability (64,65). Therefore, the effect of PS chirality on biological activity of the corresponding siRNAs appears to be somewhat sequence-dependent. In the data shown, the benefits of controlling the PS chirality were found to be more pronounced for the *Ttr-*targeted siRNA compared to the *C5-*targeted siRNA. This sequence specificity was further exacerbated *in vivo*, where metabolic stability can play a stronger role.

Our *in vitro* analyses of metabolic stability showed that the *S*p configuration at the 3′ end protects the antisense strand against the 3′ exonuclease SVPD. It is well established that SVPD preferentially cleaves the *R*p diastereoisomer, at least for single-stranded oligonucleotides. Our data are also congruent with results by Koziolkiewicz *et al.* (25) and Gilar *et al.* (68) who found that a 3′ exonuclease present in human plasma preferentially cleaves *R*p but not *S*p PS linkages in oligonucleotides. This exonuclease was later identified as ectonucleotide pyrophosphatase/phosphodiesterase 1 ENPP1 (69). This stability difference between *R*p and *S*p at the 3′ end could, at least in part, explain the potency differences observed *in vitro* by free uptake indicating a preference for the *S*p configuration. In this assay, the siRNAs are exposed to extra- and intracellular nucleases for an extended period of time.

The 5′ exonuclease PDII is known to preferentially cleave the *S*p stereoisomer in fully PS-modified single-stranded oligonucleotides (70). We did not observe any degradation of single-stranded sense strands containing either *R*p or *S*p PS linkages on the 5′ end when the siRNA had a 2′-OMe 5′-terminal nucleotide. However, when a 2′-F 5′-terminal nucleotide was used in these sense strands, PS stereochemistry had a significant impact on metabolic 5′ exonuclease stability, with the *R*p isomer providing higher stability than the *S*p configuration. This observation highlights the additional impact that other chemical modifications, such as 2′ modifications, can have on metabolic stability.

**Figure 13.**
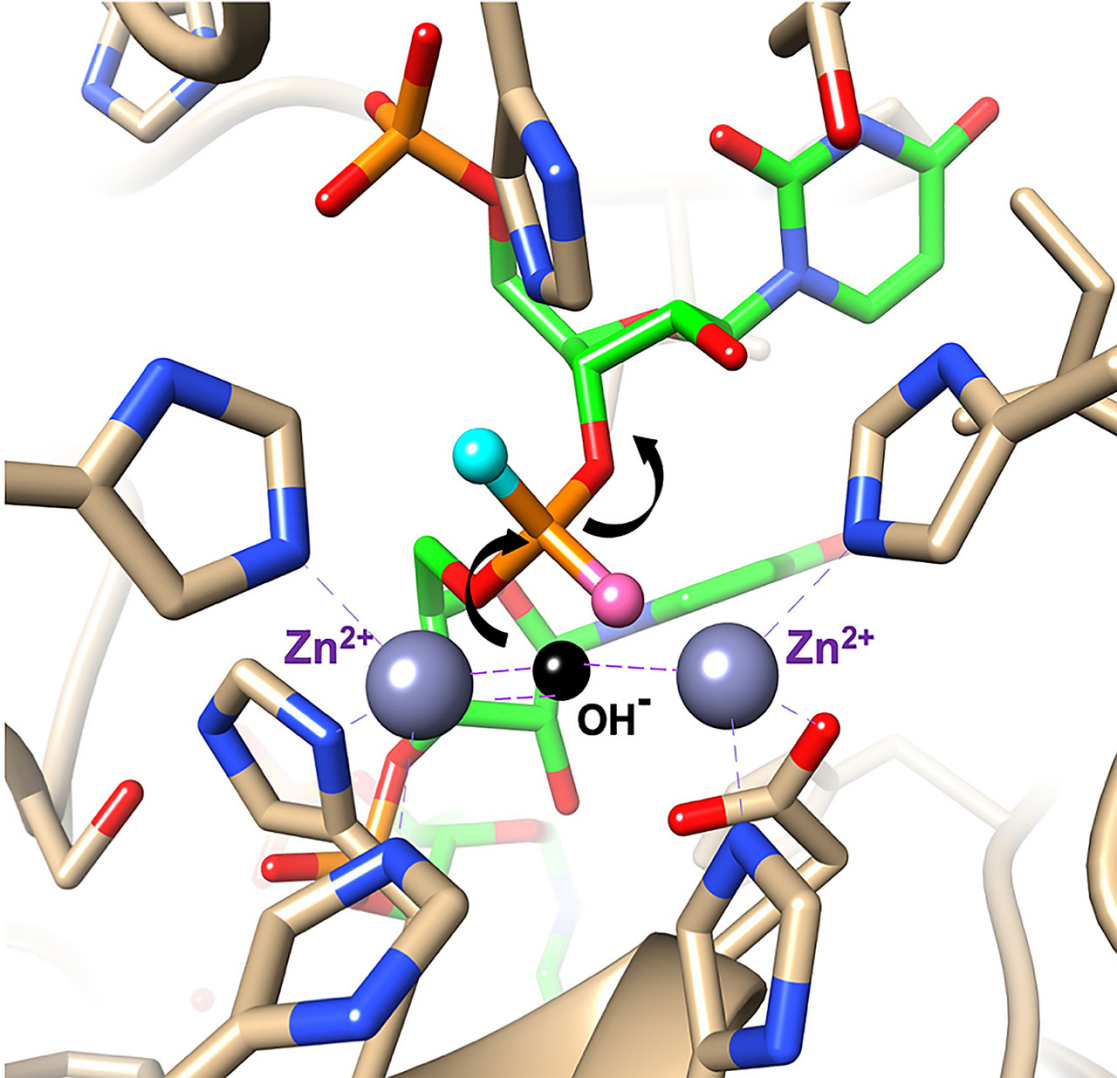
View of the active site in the crystal structure of the 5′→3′ exonuclease RNase J in complex with an oligoribonucleotide (green carbon atoms). The catalytic Zn^2+^ ions are coordinated (dashed lines) to histidines and the 5′-terminal phosphate group can be seen at the upper left. The pro-*R*p phosphate oxygen (rose sphere) points toward the metal ions, whereas the pro-*S*p oxygen (cyan sphere) is turned away. Therefore, an *S*p–PS linkage is expected to be more readily hydrolyzed by the enzyme. The postulated position of the hydroxide ion is indicated by a black sphere and arrows show the direction of the nucleophilic attack and the departing 3′-oxygen.

In addition to these observations, our *in vitro* and *in vivo* data clearly demonstrate a strong advantage for siRNAs containing the *R*p and *S*p stereochemistries on the 5′ and 3′ ends of the antisense strand, respectively. Although our *in vitro* metabolic stability assay was not able to tease apart 5′ exonuclease stability differences in the context of the 2′-OMe modification at the 5′-terminal nucleotide, we demonstrated the better 5′ exonuclease stability of the *R*p isomer in the context of the 2′-F modification. Moreover, the antisense strand 5′ PS stereochemistry with the 2′-OMe modification had a clear impact *in vivo*. It may be that 5′ exonucleases present *in vivo* are inhibited by the *R*p stereochemistry. For example, in the crystal structure of the complex between an oligoribonucleotide and the bacterial 5′→3′ exonuclease RNase J (PDB ID: 4XWW), the pro-*R*p phosphate oxygen is directed toward the two catalytic metal ions (Zn^2+^), whereas the pro-*S*p oxygen is slightly turned away (Figure 13) (71). The average distance between the pro-*R*p phosphate oxygen and the Zn^2+^ ions is 3.4 Å, and the corresponding distance in the case of the pro-*S*p oxygen is 4.3 Å. Thus, the *R*p–PS stereochemistry is likely more disruptive to the catalytic mechanism of this 5′-exonuclease than the *S*p–PS stereochemistry.

Another possible explanation is that the PS stereochemistry at the 5′ end of the siRNA antisense strand influences 5′ phosphorylation by kinases. The installment of a 5′-monophosphate group on the antisense strand of siRNA is crucial for RNAi activity (72). Although we observed no preference for either 5′-PS stereoisomer on the efficiency of 5′ phosphorylation in an *in vitro* phosphorylation assay by Clp1 kinase (Supplementary Table S8 and Figure S22), this isolated single kinase may not be representative of the entire spectrum of intracellular kinase activity.

At the 3′ end of the antisense strand, *S*p isomers were superior in the *in vivo* experiments and were more efficiently loaded into Ago2 than the 3′ *R*p isomers. It is well established that the 3′ end of the siRNA antisense strand interacts with the PAZ domain (62) of Ago2 and that chemical modifications can influence this interaction (73). Indeed, our molecular model showed that the molecular milieu surrounding the 3′ end of the antisense strand when it is bound to PAZ favors the *S*p over the *R*p PS configuration. Although it could be argued that improved Ago2 loading *in vivo* is a consequence of the improved metabolic stability due to the *S*p PS configuration, results of the *in vitro* Ago2 loading assay suggested otherwise, in that we clearly observed an Ago2 loading benefit for the 3′ *S*p isomer in the antisense strands (Figure 12A) and the presence of nuclease inhibitors in this assay negates metabolic stability as a factor.

**Figure 14.**
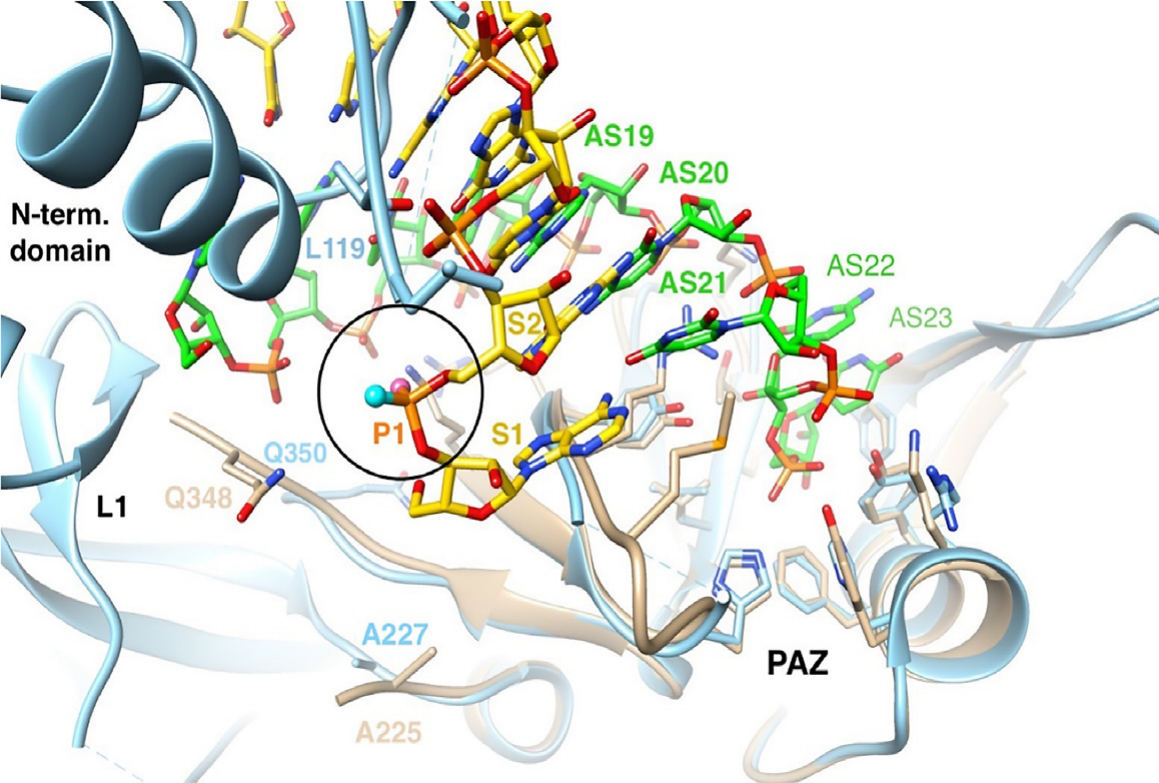
Environment of the siRNA sense strand (S, yellow carbons) 5′ end paired opposite the siRNA antisense strand (AS, green carbons) and bound to the PAZ domain (residues 229 to 348 in human Ago2, light blue cartoon). The illustration shows an overlay of residues 227–346 from the structure of the human Ago1 PAZ domain (tan cartoon) bound to an siRNA-like duplex (PDB ID: 1SI3, (62)) and residues 229–348 from the structure of full-length human Ago2 in complex with miR-20a (PDB ID: 4F3T (61). The PAZ and N-terminal domains and linker L1 are labeled, selected S and AS siRNA and Ago residues are numbered, and the pro-*R*p and pro-*S*p oxygens of the first phosphate (circled) are highlighted as balls colored in pink and cyan, respectively.

Resistance of the *R*p PS stereoisomer to 5′ exonucleases may explain the subtle impact of the *R*p isomer at the 5′ end of the sense strand observed for the *Ttr*-targeted siRNA *in vivo*. Looking more closely at the sense strand, the 3′ end is protected from nucleases by the GalNAc ligand, linker, and the scaffold and only the 5′ end needs further analysis. From an Ago2 interaction point of view, the first bridging phosphate at the 5′ end of the sense strand (S1)–if it were paired to the antisense strand residue AS21–is neither close to a residue from the PAZ domain (amino acids 229–348) that captures AS22 and AS23 nor the N-terminal (amino acids 53–140) and L1 domains (amino acids 141–228). Analysis of the PAZ domain bound to an siRNA with S1 paired to AS21 and S2 paired to AS20 demonstrates that the pro-*R*p and pro-*S*p oxygens of the terminal S phosphate are indistinguishable from each other based on the lack of direct interactions with Ago2 residues (Figure 14). Hence, nuclease resistance becomes the determining factor rather than protein-phosphate interactions. Therefore, as in the case of the 5′- and 3′-terminal AS phosphates, i.e. *R*p should be more protective against attack by 5′ exonucleases like PDII. Complicating this picture is the fact that other nucleases may be involved *in vivo*. This is consistent with the sequence-dependence of the effect of PS chirality observed with the *Ttr*-targeted siRNAs (5′AA) and the *C5*-targeted siRNAs (5′GA).

In summary, we have shown that defined PS stereochemistry at both ends of the siRNA antisense strand can significantly impact the pharmacological properties compared to their stereo-random parental designs. We also demonstrated that the efficacies of the siRNAs with optimal stereoisomer configuration (S/R-S and R/R-S) surpass that of the design with three stereo-random PS linkages and match the efficacy of siRNAs with six stereo-random PS linkages. This suggests that, in rodents, the number of PS linkages can be reduced from six to three without compromising metabolic stability and activity of these stereo-defined siRNAs. This would allow reduction of the number of PS-containing monomer residues if needed (74,75). The results discussed here have been presented in an earlier disclosure (https://patents.google.com/patent/WO2019126651A1/en). Another recent report by Sakamuri *et al.* (36,51) confirmed our findings in another target, *antithrombin*. We believe that, for the first time, the present account provides a comprehensive molecular basis for this chiral selectivity based on both metabolic stability analysis and Ago2-mediated RNAi mechanistic analysis utilizing known structures of the oligonucleotide modifications and proteins involved. Further investigation is required to determine whether the chiral PS approach with six PS linkages with well-defined chirality will enhance *in vivo* potency and metabolic stability and hence duration of gene silencing compared to the current clinical design with six stereo-random PS linkages.

## Supplementary Material

gkab544_Supplemental_File

## Notes

Present address: Kallanthottathil G. Rajeev, Verve Therapeutics, 500 Technology Square, Suite 901, Cambridge, MA 02139, USA.
