# Supplementary material for: Chirality matters: stereo-defined phosphorothioate linkages at the termini of small interfering RNAs improve pharmacology in vivo
Source: Nucleic Acids Res. 2021 Jul 15;50(3):1221–40. doi: 10.1093/nar/gkab544 (PMC8860597; doi:10.1093/nar/gkab544)
Supplement: gkab544_Supplemental_File [file gkab544_supplemental_file.docx]

**Supporting Information**

**Chirality Matters: Stereo-defined phosphorothioate linkages at the termini of small interfering RNAs improve pharmacology *in vivo***

Hartmut Jahns, Nate Taneja, Jennifer L.S. Willoughby, Masaaki Akabane-Nakata, Christopher Brown, Tuyen Nguyen, Anna Bisbe, Shigeo Matsuda, Matt Hettinger, Rajar M. Manoharan, Kallanthottathil G. Rajeev,^1^ Martin A. Maier, Ivan Zlatev, Klaus Charisse, Martin Egli,^2^ Muthiah Manoharan*

^1^Hartmut Jahns and Nate Taneja made equal contributions to this work.

Alnylam Pharmaceuticals, 675 W. Kendall St, Cambridge, Massachusetts 02142, United States

^2^Department of Biochemistry, School of Medicine, Vanderbilt University, Nashville, Tennessee 37232, United States

^1^Present address: Verve Therapeutics, 500 Technology Square, Suite 901, Cambridge, Massachusetts 02139, United States

* To whom correspondence should be addressed. Email: [mmanoharan@alnylam.com](mailto:mmanoharan@alnylam.com)

**Table of Contents**

I.Separation of phosphorothioate (PS) isomers in [oligonucleotide and assignment of stereochemistry of the PS linkageS3](#_Oligonucleotide_synthesis_and)

[Purification of sense strands si2-S-F and si2-S into two diastereomersS3](#_Purification_of_sense)

[Purification of antisense strand si2-AS into four diastereomersS6](#_Purification_of_antisense)

[Methods of assignment of stereochemical configurations for purified isomersS10](#_Methods_of_assignment)

[Diastereomer identification of sense strands si2-S-F and si2-SS12](#Identificationofsensestrands)

[Diastereomer identification of antisense strand si2-ASS15](#Identificationofantisensestrand)

II. [Stereo-defined dinucleotide synthesis and dinucleotide separation approachS20](#Chirallypuredinucleotidesynthesis)

[Synthetic schemes, procedures, and characterization of stereo-defined dinucleotidesS20](#_Scheme_S1._Synthesis)

[^1^H, ^13^C, ^19^F, and ^31^P NMR spectra for stereo-defined dinucleotidesS41](#NMR)

III. [Exonuclease study for fully deprotected, stereo-defined dinucleotidesS172](#Exonucleasestudy)

IV. [Methods of assignment of absolute configurations of synthesized stereo-defined oligonucleotidesS173](#_Methods_of_assignment_1)

[Identification of mrTTR oligonucleotides synthesized using stereo-defined dinucleotidesS175](#IdentificationmrTTR)

[Identification of C5 oligonucleotides synthesized using stereo-defined dinucleotidesS176](#IdentificationC5)

V. [Oligonucleotide characterizationS179](#_Oligonucleotide_characterization)

VI. Analyses of whether stereo-defined oligonucleotides serve as [Clp1 kinase substratesS181](#_Clp1_kinase_experimen)

VII. [References](#_References)S183

## ***Separation of phosphorothioate (PS) isomers in oligonucleotide and assignment of stereochemistry of the PS linkage***

## ***Purification of sense strands si2-S-F and si2-S into two diastereomers***

Two GalNAc-conjugated sense strands, si2-S-F and si2-S were synthesized, each with one PS on the 5ʹ end (Table in Figure S1). Purification was performed DMT-on, which allowed efficient separation of 5ʹ end phosphorothioate isomers.

| **Strand ID** | **Sequence (5ʹ-3ʹ)** | **Mass (m/z)** | |
| --- | --- | --- | --- |
|  |  | **calc.** | **obs.** |
| si2-S-F | G●aCaAaAuAACuCaCuAuAaU(L) | 8611.2 | 8609.6 |
| si2-S | g●aCaAaAuAACuCaCuAuAaU(L) | 8623.3 | 8621.9 |


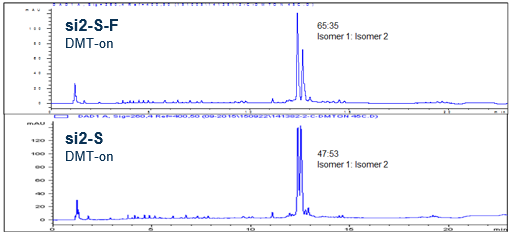


Figure S1. Upper table: Sequences and composition of strands. Uppercase and lowercase letters represent 2′-F-RNA and 2′-OMe, respectively, to adenosine (A), cytidine (C), guanosine (G), and uridine (U). (L) represents the GalNAc ligand (1). Phosphorothioate mixtures are indicated by ●. Lower: IEX chromatograms of DMT-on crude si2-S-F and si2-S.

Samples were diluted to approximately 0.03 mg/mL with water and 30 µL was injected onto a Dionex DNAPac PA200 ion-exchange (IEX) analytical column (4 mm x 250 mm; ThermoFisher, cat #063000). Buffer A was 20 mM sodium phosphate (pH 11,) 15% acetonitrile, and Buffer B was 20 mM sodium phosphate (pH 11,) 15% acetonitrile, 1M sodium bromide. A gradient of 31% to 57% Buffer B over 16 min, and a flow rate of 1 mL/min was used to analyze these samples. The column temperature was maintained at 45 °C as separation was slightly better at this temperature than at 30 ºC. The isomer separation is shown in Figure S1.

A different anion exchange resin was used to purify these compounds into their respective isomers (Source 30Q, GE Healthcare, cat #17-1275-03). This resin was manually packed into a 2-cm diameter glass column (Waters Corporation, AP-2 Glass Column, 20 mm x 300 mm, cat #WAT027503) according to packing instructions for Source 30Q resin and the AP-2 column. There is a hydrophobic component to the resin matrix that resulted in good separation of 5′ end isomers with DMT-on. Buffer A was 20 mM sodium phosphate (pH11), 15% acetonitrile, and Buffer B was 20 mM sodium phosphate (pH 11), 15% acetonitrile, 1 M sodium bromide. The gradient used was 15% to 55% Buffer B in 150 min at a flow rate of 10 mL/min. This gradient is equivalent to approximately 21 column volumes. Temperature of the column was 65 °C. Three to five runs were performed for each compound, with approximately 800 OD/run. This procedure resulted in adequate amounts of each isomer at >85% purity (Figure S2).


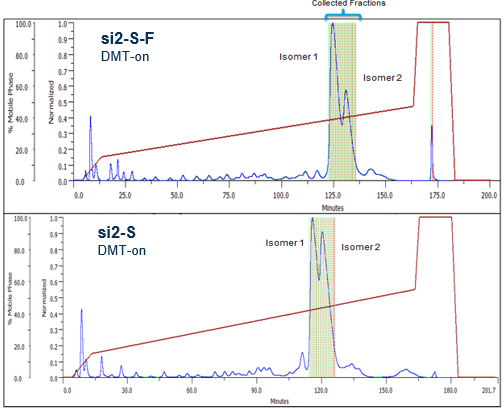


Figure S2. Chromatograms showing separation of PS isomers of si2-S-F and si2-S. Fractions were analyzed by IEX, pH 11, and fractions of >85% purity for each isomer were combined.

The resulting DMT-on pools for the four isomers were dried, resuspended in water, and desalted over size-exclusion columns (GE Healthcare, cat #17-5087-01) with a flow rate of 10 mL/min, dried again, and detritylated by adding 5 mL of 20% acetic acid at room temperature for 10 minutes. Following this procedure each pool was neutralized by adding 18 mL of a saturated sodium bicarbonate solution. These solutions were dried, and oligonucleotides were again desalted by size exclusion. PS to PO conversion was not observed during the detritylation procedure, and repurification was not needed. The IEX, pH 11, chromatograms are shown in Figure S3 and Figure S4. A difference in column temperature accounts for the significant difference in retention time between si2-S-F (Figure S3, 45 °C) and si2-S (Figure S4, 30 °C). Quality control data for all compounds are reported in sections below.


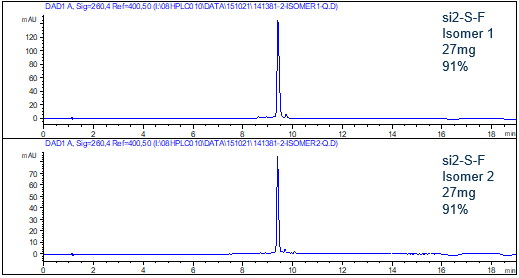


Figure S3. IEX, pH 11, chromatograms of isomers for sense strand si2-S-F (31-57% in 16 minutes, 1 mL/min, using a Dionex DNAPac PA200 4mm x 250mm column).


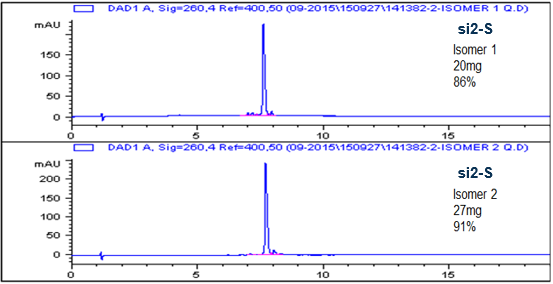


Figure S4. IEX, pH 11, chromatograms of isomers of si2-S (31-57% in 16 minutes, 1 mL/min, using a Dionex DNAPac PA200 4mm x 250mm column).

***Purification of antisense strand si2-AS into four diastereomers***

For the initial studies, an oligonucleotide with two phosphorothioate linkages was evaluated, one on the 5ʹ end and one on the 3ʹ end. Oligonucleotide si2-AS (5ʹ- a●UuAuAgUgAguuAuUuUgUca●a-3ʹ) has four diastereomers. Samples were diluted to approximately 0.03 mg/mL with water, and 30 µL of sample was injected onto a Dionex DNAPac PA200 IEX analytical column, 4 mm x 250 mm (ThermoFisher, cat #063000). Buffer A was 20 mM sodium phosphate, 15% acetonitrile, pH 11, and Buffer B was identical with the addition of 1 M sodium bromide. A gradient of 40% to 68% over 16 min with a flow rate of 1 mL/min was used to analyze DMT-on samples. A gradient of 31% to 57% over 16 minutes was used to analyze DMT-off samples (Figure S5). The column temperature was maintained at 30 °C. Baseline separation of all four diastereomers of DMT-on si2-AS was obtained. After DMT removal, only the 3ʹ end isomers were resolved.

| **Strand ID** | **Sequence (5ʹ-3ʹ)** | **Mass (m/z)** | |
| --- | --- | --- | --- |
|  |  | **calc.** | **obs.** |
| si2-AS | a●UuAuAgUgAguuAuUuUgUca●a | 7542.7 | 7541.3 |


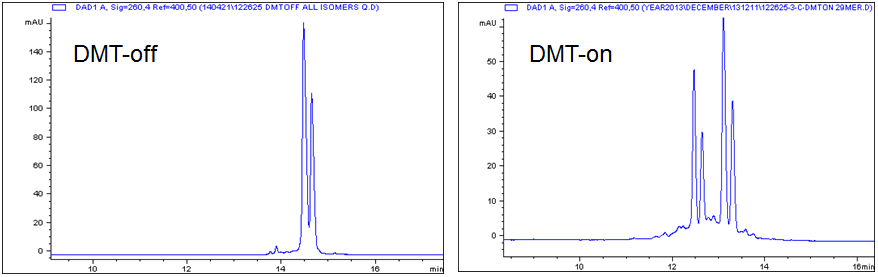


Figure S5. Upper table: Sequence and composition of strand. Uppercase and lowercase letters represent 2′-F-RNA and 2′-OMe, respectively. Phosphorothioate mixtures are indicated by the ●. Lower: IEX chromatograms of DMT-off (left) and DMT-on (right) si2-AS.

This analytical method was scaled up and adjusted to achieve isomer separation at preparatory scale. An anion-exchange Dionex DNAPak PA200 (22 mm x 250 mm) prep column (ThermoFisher, cat #SP6734) was used and Buffers A and B were identical to the analytical buffers. The gradient was 42% to 57% Buffer B in 45 minutes at a flow rate of 12 mL/min, corresponding to 5.7 column volumes, and column temperature was maintained at 40 °C. In order to achieve best possible separation, ~3.2 mg of crude material was loaded per run. Even so, this method alone could not generate isomer purities close to the target of ≥85% for three of the four isomers. Therefore, fractions were combined to create enriched isomer pools and repurified. The purest regions for each of the four isomers were pooled across runs; a total of 50 runs were performed in this manner (Figure S6).

Isomer impurities flanking the enriched isomer product peak in each pool were separable post-DMT removal as confirmed by mixing these isomers in small amounts after DMT removal and analyzing by IEX, pH 11, chromatography. The four isomer pools were dried down, desalted over size exclusion columns (GE Healthcare, cat #17-5087-01) with a flow rate of 10 mL/min, dried down again, and detritylated by adding 5 mL of 20% acetic acid at room temperature for 10 min. Each pool was then neutralized by adding 18 mL of a saturated sodium bicarbonate solution. Samples were dried, dissolved in water, and desalted again by size exclusion. Each isomer pool was repurified to eliminate remaining isomer impurities as well as oligonucleotides in which PS to PO conversion had occurred during the detritylation procedure.


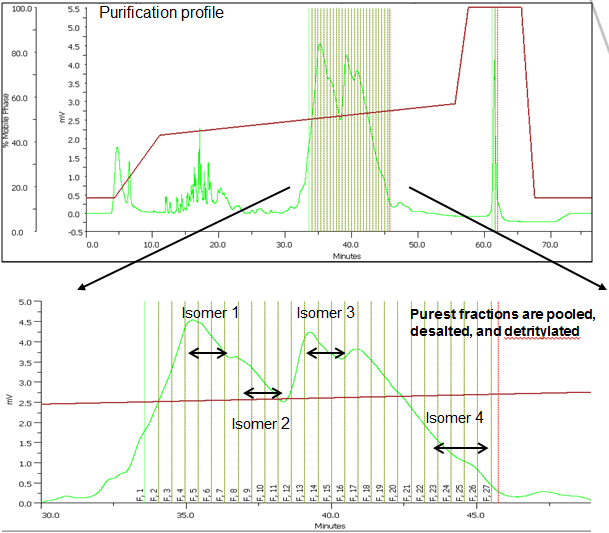


Figure S6. Anion-exchange purification of isomers from the crude isomer mixture. The pooled regions for each isomer are shown. The method was reproducible, and these regions could be pooled without analysis of the fractions from every run. Two to four 5.5-mL fractions were pooled for each isomer per run with retention times between 35-36.5 min for isomer 1, 37-38 min for isomer 2, 39-40.5 min for isomer 3, and 43.5-45.5 min for isomer 4.


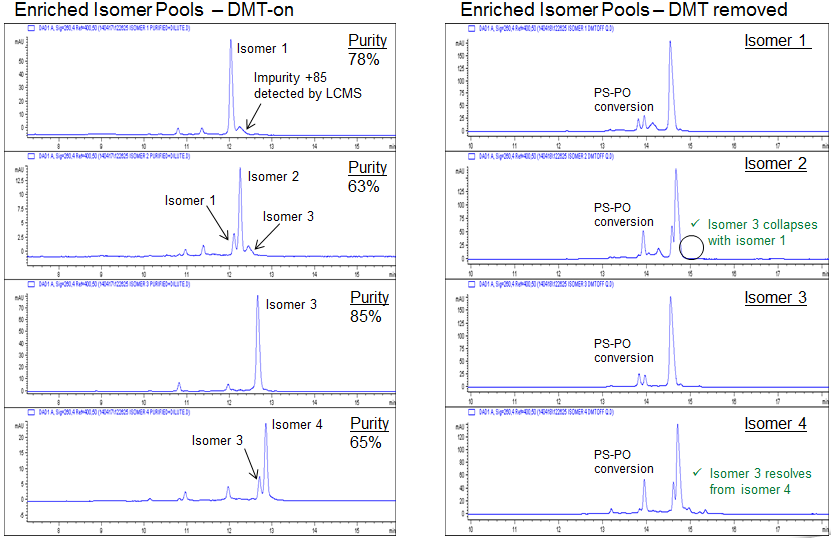


Figure S7. Fraction pools analyzed before and after DMT removal (right and left columns, respectively) by IEX chromatography. Samples were also analyzed by LC-MS to identify any non-isomer impurities by mass.

To determine purity, IEX analysis was performed using the Dionex DNAPac PA200 column (4 mm x 250 mm) at a flow rate of 1 mL/min. The gradient for DMT-on material was 40% to 68% Buffer B in 16 min. For DMT-off material, the gradient was 31% to 57% Buffer B in 16 min. Peak area integration at 260 nm was used to determine purity. Reverse-phase LC-MS analysis was used to detect mass impurities. The column used was Waters XBridge (C8 2.5 µm, 2.1 mm x 50 mm; PN 186003101). Buffer A was 200 mM hexafluoroisopropanol and 16 mM triethylamine in water. Buffer B was 100% methanol. All reagents were LC-MS grade. The gradient was 0% to 40% methanol in 9.6 min, flow rate 0.7 mL/min.

The IEX chromatograms are shown in Figure S7. Prior to DMT removal, minor amounts of DMT loss (-302 Da) and PS-PO conversion (-16 amu) were observed. In the case of isomer 1 an unknown impurity +85 Da was detected by LC-MS, which was assumed to be the later eluting peak seen in the IEX chromatogram, because isomer 2 was not seen in the fractions pooled. In addition, it was confirmed that isomers 1 and 2 were resolved post-DMT removal by the IEX, pH 11 analytical method. Therefore, isomer 2 was not a significant impurity in the fraction pool for isomer 1. Pools of isomers 2 and 4 contained significant impurity isomers, whereas isomer 3 was fairly pure with only PS to PO conversion observed after detritylation. PS-PO conversion was seen in all cases after the acetic acid treatment used for detritylation.

Once desalted after removal of DMT, each enriched isomer pool was repurified using the same Dionex DNAPac PA200 column that was used for the original purification. The gradient used was 35% to 51% Buffer B in 45 min but all other parameters (buffers, temperature, flow rate) were the same. Isomers 2 and 4 remained below the target purity of 85% as shown by IEX, pH 11 analysis shown in Figure S8, but material was of inadequate amount for further repurification attempts. It was determined that purities were adequate for detection of significant differences, if present, in biological assays, and these compounds were used in *in vivo* and *in vitro* studies.


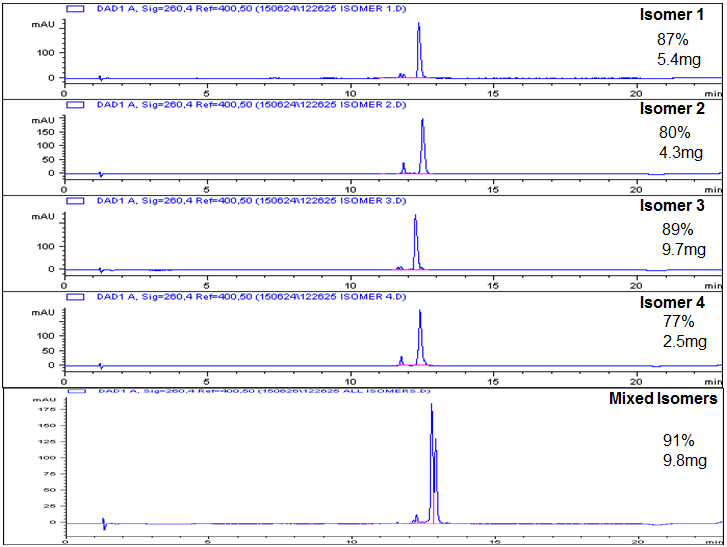


Figure S8. IEX analysis of isomers of si2-AS. Isomers 2 and 4 were the most difficult to separate due to their lower abundance. Purities were accepted as 80% and 77% for isomers 2 and 4, respectively.

## A compound containing the isomer mixture at standard synthesis ratios was also needed. This compound was synthesized DMT-off and purified by IEX chromatography using 20 mM sodium phosphate, 15% acetonitrile, pH 8.5 for buffer A; Buffer B also contained 1 M sodium bromide. The purification was performed on the Waters 2-AP column packed with TSKgel Super Q-5PW (20) anion exchange resin from Tosoh Corporation (cat #0018546). A gradient of 17% to 42% Buffer B over 150 min was employed. The flow rate was at 10 mL/min. Fractions were analyzed via IEX, pH 11, and fractions of >85% purity were pooled. This pool was dried, resuspended in water, and desalted by size-exclusion chromatography as described above.

##

## ***Methods of assignment of stereochemical configurations for purified isomers***

To predict stereochemistry, several methods were employed. One tool was knowledge of the synthesis ratios of *R*_p_ vs. *S*_p_, which depend on 2ʹ-modified amidites being coupled on either side (3ʹ and 5ʹ) of the PS linkage. During synthesis these ratios are not significantly affected by scale or position of the dinucleotide within a sequence as long as protecting groups on the phosphate (cyanoethyl) and activator type (0.6 M ETT) are constant. Synthesis ratios were determined for all possible 2ʹ-F and 2ʹ-OMe (cyanoethyl protected) dinucleotides using 0.6 M ETT as activator. Therefore, as long as isomers within a given sequence were separable by some analytical method, *R*_p_ or *S*_p_ assignments could be predicted by performing peak integration. Of course, this method could not be employed when then predicted ratio is close to 50/50.


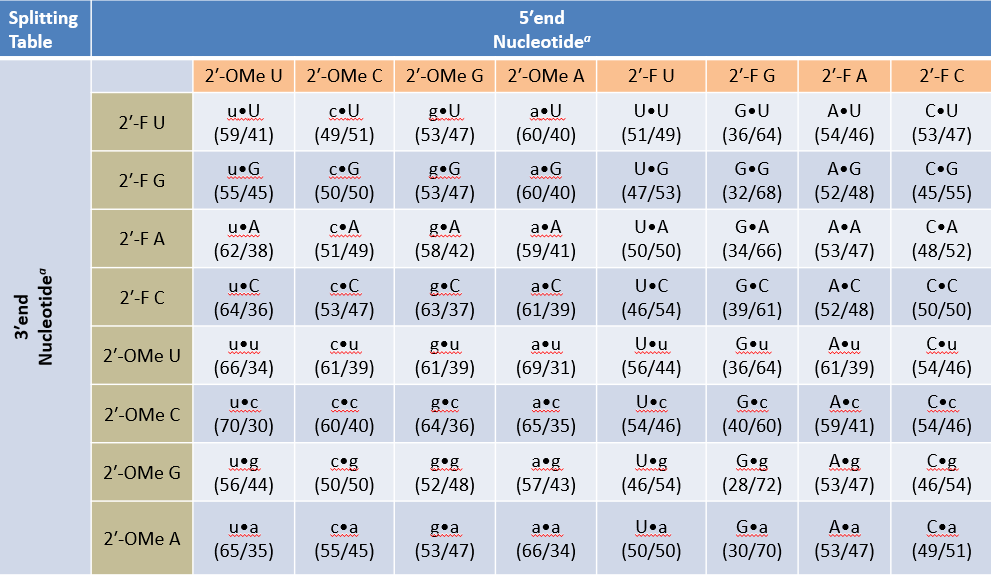


Table S1. Diastereomer ratios for 2ʹ-F and 2ʹ-OMe dinucleotides. Upper table: Sequence and composition of dinucleotide. ^a^Uppercase and lowercase letters represent 2′-F-RNA and 2′-OMe. Phosphorothioate mixtures are indicated by the ●. Lower table: Ratios of downfield diastereomer (R_p_) to upfield diasteromer (S_p_).

Dinucleotides (Table S1) were synthesized via solid-phase synthesis at 40 µM scale on universal support on an ABI synthesizer using 0.6M ETT as activator and PADS as sulfurizing reagent. Ammonia was removed post-deprotection by evaporation (centrifuge under vacuum), and the resulting material was frozen and lyophilized. Samples were prepared for ^31^P-NMR analysis by dissolving 5 mg lyophilized product in 500 µL of deuterium oxide. ^31^P-NMR was used to determine the diasteromer ratio in these dinucleotides for two reasons. First, the isomers always resolved in the 55-60 ppm range. In IEX or reverse-phase analysis peak splitting was not observed for all the dinucleotides. Second, the *R*_p_ isomer is downfield shifted (farther from 0) compared to the *S*_p_ isomer for fully deprotected nucleotides (2-5). Some protecting groups have been found to reverse this rule (5).

^31^P-NMR was also used as an analytical method to determine whether only the *R*_p_ or *S*_p_ isomer was present in the final purified compounds versus a mixture of *R*_p_ and *S*_p_. Although this was not always possible due to extremely low signal, confirmations that were made in this manner are shown below.

A third method utilized to identify isomers was IEX analysis. The *R*_p_ isomer is predicted to elute earlier than *S*_p_ in reverse-phase analysis (2,6-9) with the following exception: If the phosphorothioate linkage is on the 5ʹ end, and the DMT protecting group is left on, the *S*_p_ isomer elutes before the *R*_p_ isomer (6,8,9). We found the same rules applied in IEX analysis. IEX analysis was utilized in all cases studied here to aid in identification of the diasteromers. The DMT-on “flip” of the elution pattern in reverse-phase and IEX chromatography of PS isomers on the 5ʹ end was an important observation that was utilized to confirm isomer configurations of 5ʹ-end PS.

The fourth method to aid in the identification of isomers, particularly on the 3ʹ end, was through analysis of degradation by exonucleases (2,5). The *S*_p_ isomer is more stable against snake venom phosphodiesterase (SVPD), a 3ʹ exonuclease, than the *R*_p_ isomer. Additionally, analyses using phosphodiesterase II (PDII), a 5ʹ exonuclease (isolated from bovine spleen), suggest that the *R*_p_ isomer is more stable than the *S*_p_ isomer. However, if the PS bond is in combination with a 2ʹ-OMe modification, as is usually the case for these compounds, we observed little difference in degradation rates of *R*_p_ and *S*_p_ isomers by either SVPD or PDII.

***Diastereomer identification of sense strands si2-S-F and si2-S***

Both of si2-S-F and si2-S oligonucleotides contain a single PS linkage at the 5ʹ end. si2-S-F has a 2ʹ-F-G at the 5ʹ end, and si2-S has a 2ʹ-OMe-G at the 5ʹ end. Otherwise, the sequences and chemistries are identical. For si2-S-F, the predicted synthesis ratio of *R*_p_:*S*_p_ is 30:70 (Table S1), and the ratio obtained by peak integration on the IEX chromatogram of the crude compound was 34:66. For si2-S the predicted *R*_p_:*S*_p_ ratio is 53:47, and the ratio found experimentally was 53:47 (Figure S9).

| **Strand ID** | **Sequence (5ʹ-3ʹ)** | **Mass (m/z)** | |
| --- | --- | --- | --- |
|  |  | **calc.** | **obs.** |
| si2-S-F  si2-S | G●aCaAaAuAACuCaCuAuAaU(L)  g●aCaAaAuAACuCaCuAuAaU(L) | 8611.2  8623.3 | 8609.6  8621.9 |


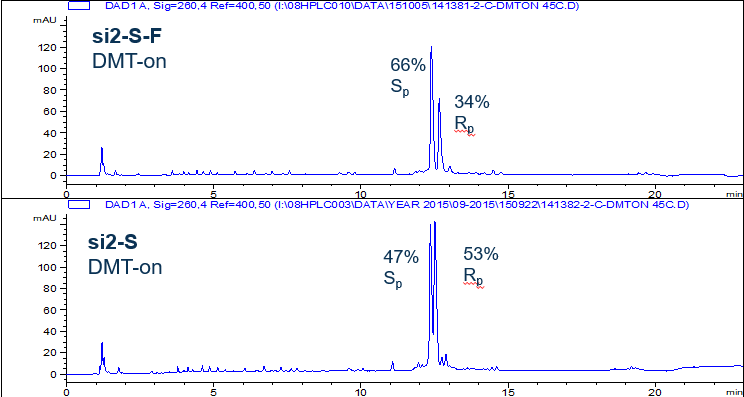


Figure S9. IEX chromatography of DMT-on si2-S-F and si2-S. Upper table: Sequences and compositions of strands. Uppercase and lowercase letters represent 2′-F-RNA and 2′-OMe. Phosphorothioate mixtures are indicated by the ●. (L) represents the GalNAc ligand. Lower chromatograms: IEX chromatograms of DMT-on si2-S-F and si2-S.

The faster migrating of the two peaks in each of the chromatograms shown in Figure S9 is predicted to have an *S*_p_ linkage, and the second peak is predicted to be *R*_p_ for two reasons. First, the predicted ratios (Table S1) match (or are very close to) the ratios determined for dinucleotides. Second, the isomers are on the 5ʹ end of the sequence, and oligonucleotides were analyzed DMT-on. Therefore, the *S*_p_ isomer should elute first. Once DMT is removed in the final product, the *S*_p_ isomer should elute second. This “flip” was confirmed by analysis of DMT-off products (data not shown). Once the isomers for each sequence were purified and DMT removed, oligonucleotides were analyzed by ^31^P-NMR analysis as 10 mg/mL solutions in deuterium oxide. The resulting ^31^P-NMR spectra are shown in Figures S10, S11, S12, and S13.


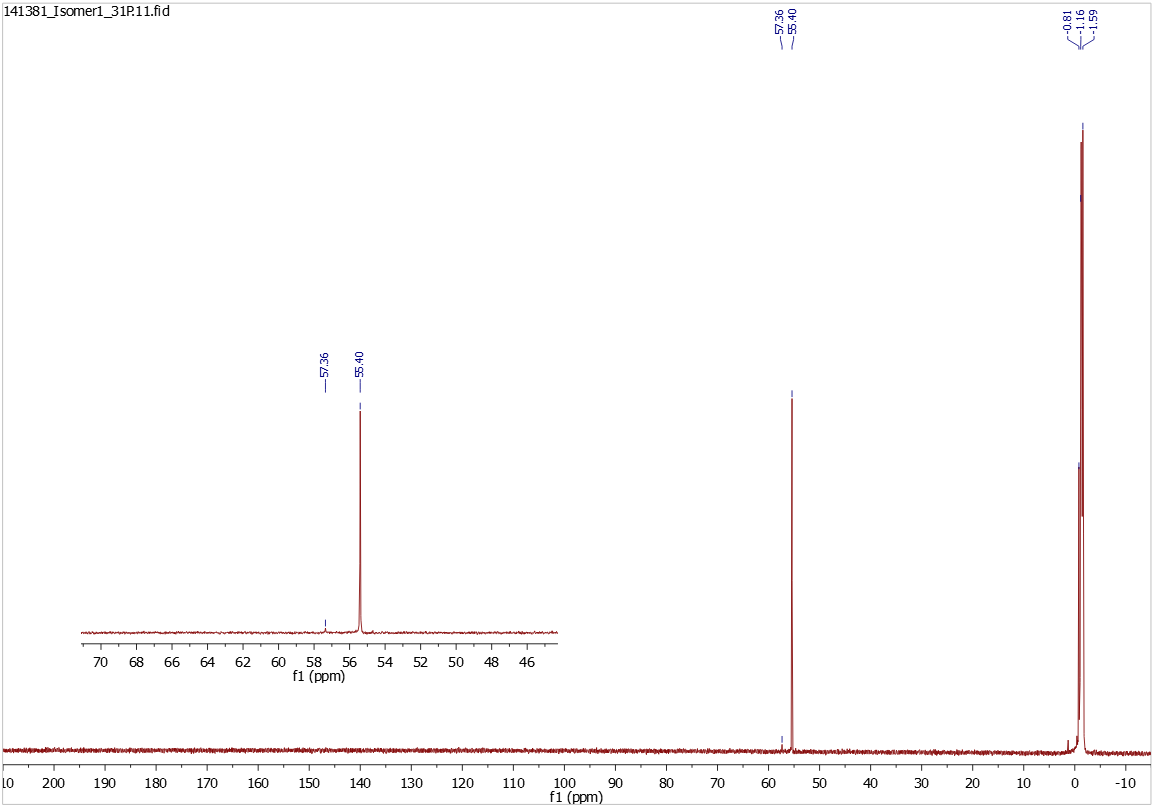


Figure S10. ^31^P-NMR spectra for isomers of si2-S-F. Isomer 1 (S_p_) shows the main resonance peak at 55.40 ppm, upfield shifted relative to the impurity R_p_ isomer at 57.36 ppm.


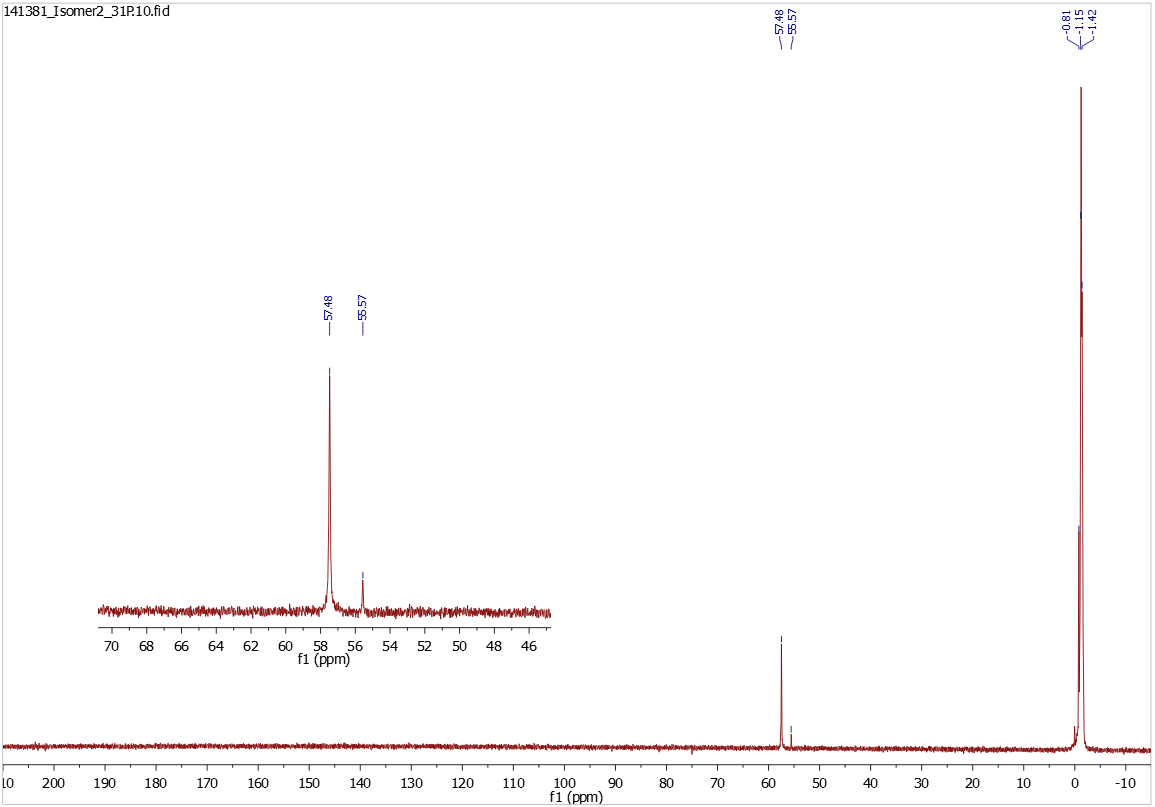


Figure S11. ^31^P-NMR spectra for isomers of si2-S-F. Isomer 2 (R_p_) shows the main resonance peak at 57.48 ppm, downfield shifted relative to the impurity S_p_ isomer at 55.57 ppm.


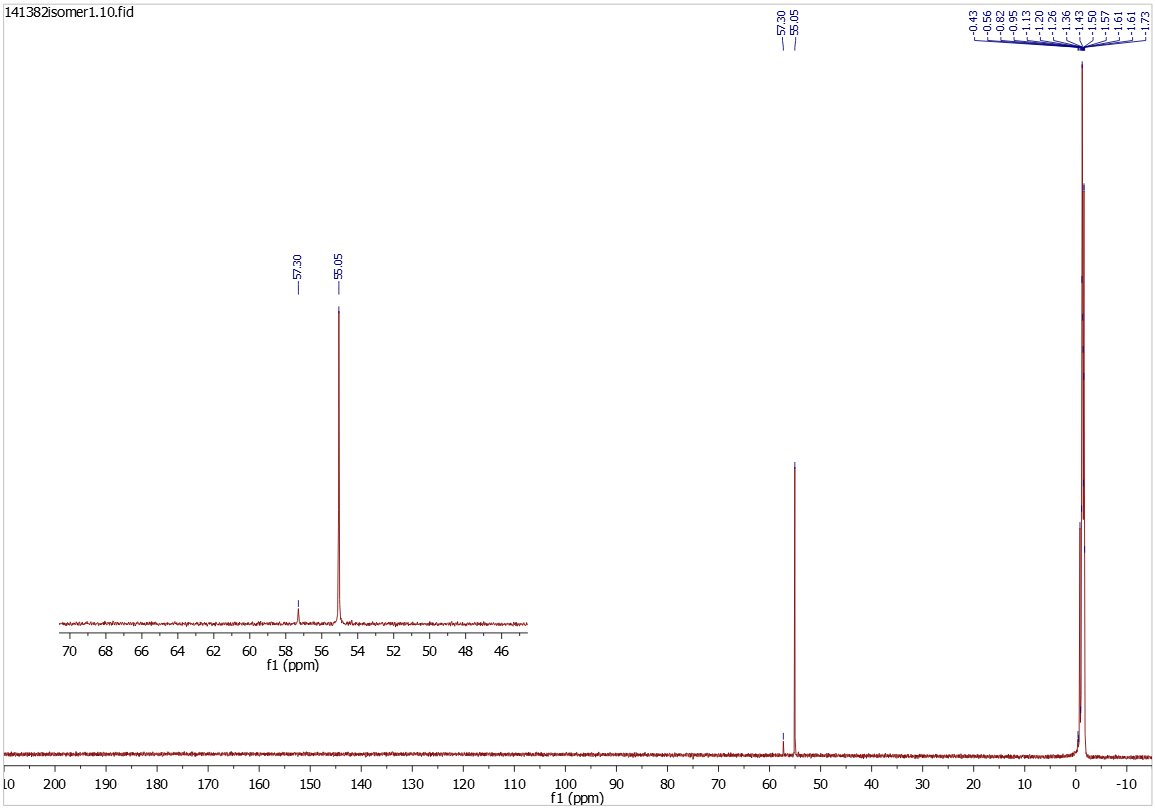


Figure S12. ^31^P-NMR spectra for isomers of si2-S. Isomer 1 (Sp) has a main resonance peak at 55.05 ppm, upfield shifted relative to the impurity (the Rp isomer) at 57.30 ppm.


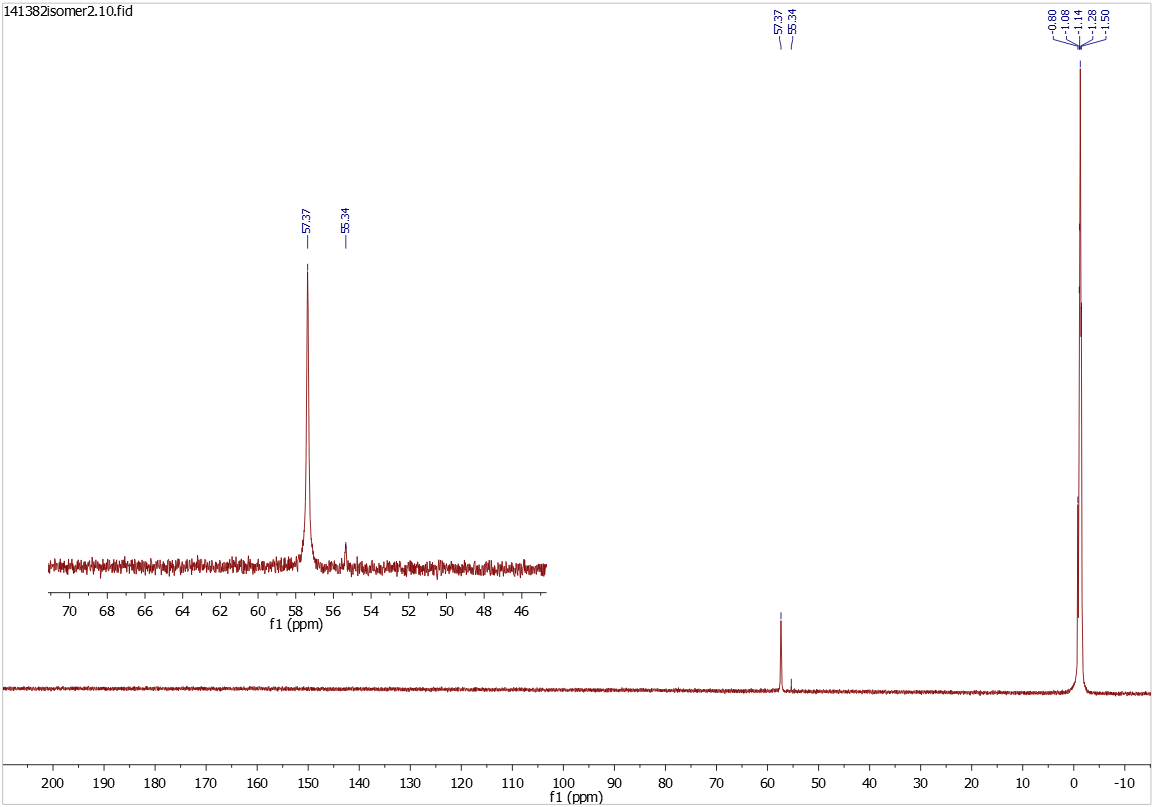


Figure S13. ^31^P-NMR spectra for isomers of si2-S-F. Isomer 2 (*R*_p_) has a main resonance peak at 57.37 ppm, downfield shifted relative to the impurity (the *S*_p_ isomer) at 55.34 ppm.

***Diastereomer identification of antisense strand si2-AS***

si2-AS contains two PS linkages, one on the 3ʹ-end and one on the 5ʹ-end of the sequence, resulting in four stereoisomers. After removal of the DMT protecting group, the 3ʹ-end isomers were separated by anion exchange analysis. The isomer assignment was confirmed by synthesis and analysis of si2-AS-1PS, which has a single PS linkage at the 5ʹ end (Table S2). si2-AS-1PS has the same sequence and chemistry as si2-AS without the PS linkage on the 3ʹ end. Only one peak is seen in IEX analysis of si2-AS-1PS (data not shown), proving that the splitting seen in IEX analysis of si2-AS results from the 3ʹ end isomers. The predicted diastereomer ratio from ^31^P-NMR analysis for the 3ʹ end a●a dimer of si2-AS is 66:34 (*R*_p_:*S*_p_) based on data shown in Table S1. After synthesis of the compound, with the 5ʹ-DMT removed, the ratio was 62:38 by IEX analysis (Figure S14).

Table S2. Sequences and chemical compositions of si2-AS and si2-AS-1PS.

| **Strand ID** | **Sequence (5**ʹ**-3**ʹ**)*^a^*** | **Number of Peaks on IEX-HPLC** | **Mass (m/z)** | |
| --- | --- | --- | --- | --- |
|  |  |  | **calc.** | **obs.** |
| si2-AS | a•UuAuAgUgAguuAuUuUgUca•a | 2 | 7542.7 | 7541.3 |
| si2-AS-1PS | a•UuAuAgUgAguuAuUuUgUcaa | 1 | 7526.7 | 7525.3 |

*^a^*Uppercase and lowercase letters represent 2′-F-RNA and 2′-OMe, respectively. Phosphorothioate mixtures are indicated by the ●.


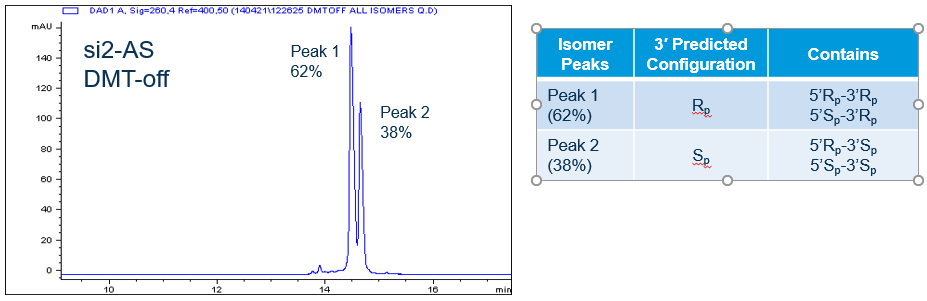


Figure S14. DMT-off IEX analysis of si2-AS. Each peak contains both 5ʹ-end isomers; isomers corresponding to the PS linkage at the 3ʹ end are resolved.

When analyzed DMT-on, the 5ʹ-end isomers as well as 3ʹ-end isomers are resolved by IEX chromatography (Figure S15). *R*_p_:*S*_p_ ratios of 59:41 and 57:43 determined for si2-AS are similar to the predicted ratio of 60:40 (Table S1). Additionally, when the four main peaks were integrated by area with the total equaling 100%, isomers 1 and 3 (the predicted 3ʹ-*R*_p_ isomers) made up 62% of the total. Isomers 2 and 4 (the predicted 3ʹ-S_p_ isomers) made up 38%. This is the *R*_p_:*S*_p_ ratio (62:38) observed in the DMT-off si2-AS (Figure S14).


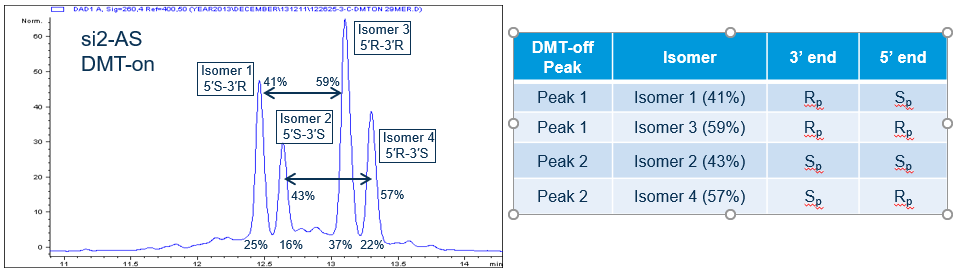


Figure S15. DMT-on IEX analysis showing peak resolution of four diastereomers of si2-AS.

The four isomers of si2-AS and the isomer mixture were analyzed by ^31^P-NMR to confirm isomer identity assignments. Compounds were prepared by dissolving lyophilized oligonucleotide in 550 µL of deuterium oxide at concentrations of 10 mg/mL. Since only 2.4 mg was obtained for isomer 4 (predicted 5ʹ*R*-3ʹ*S*), this isomer was analyzed at 4.4 mg/mL, and signals were weak and difficult to interpret. ^31^P-NMR was not obtained for isomer 1. ^31^P-NMR analyses for isomers 2, 3 and 4 as well as the isomer mixture are shown in Figures S16, S17, S18, and S19.


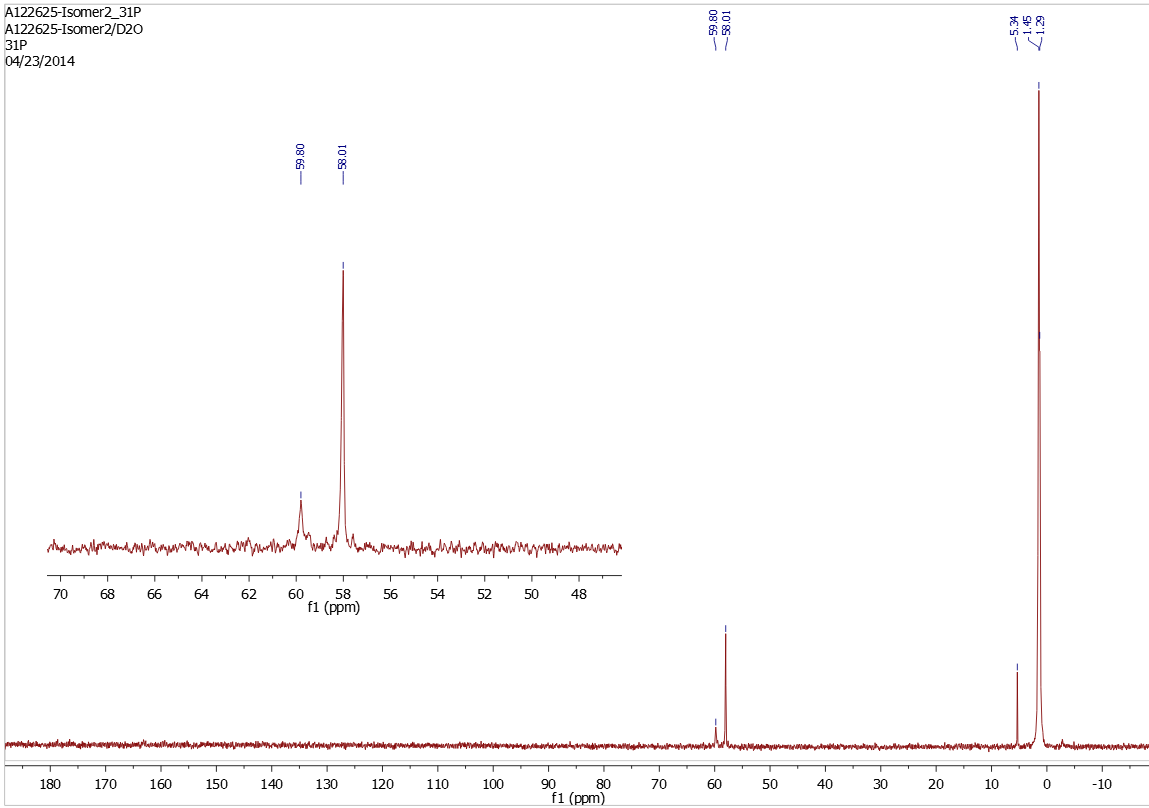


Figure S16. ^31^P-NMR spectra for si2-AS isomer 2 (5ʹS-3ʹS). Major peak at 58.01 ppm is upfield shifted relative to the peak at 59.80 ppm which is likely due to the presence of isomer 1 (predicted 5ʹS-3ʹR) and isomer 3 (5ʹR-3ʹR).


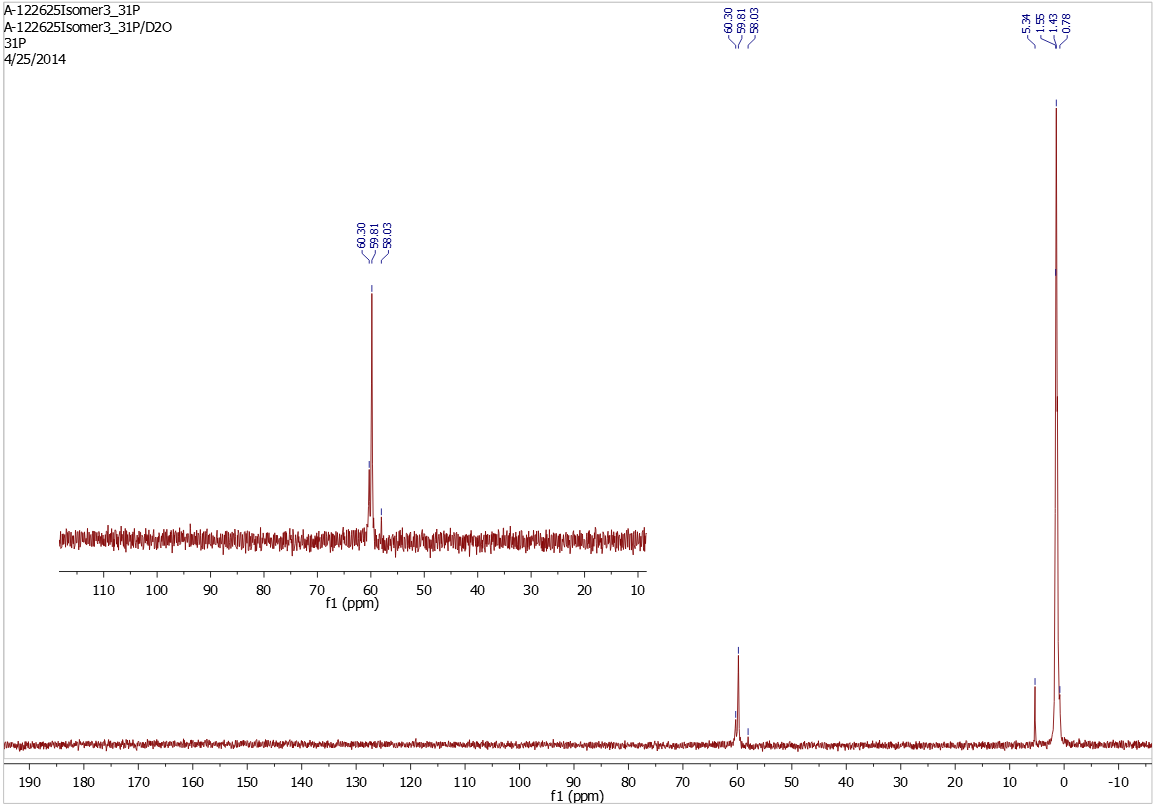


Figure S17. ^31^P-NMR spectra for si2-AS isomer 3 (5ʹR-3ʹR). The peaks due to R_p_ isomers are observed at 60.30 and 59.81 ppm. S_p_ isomer was not detected at 58.03 ppm.


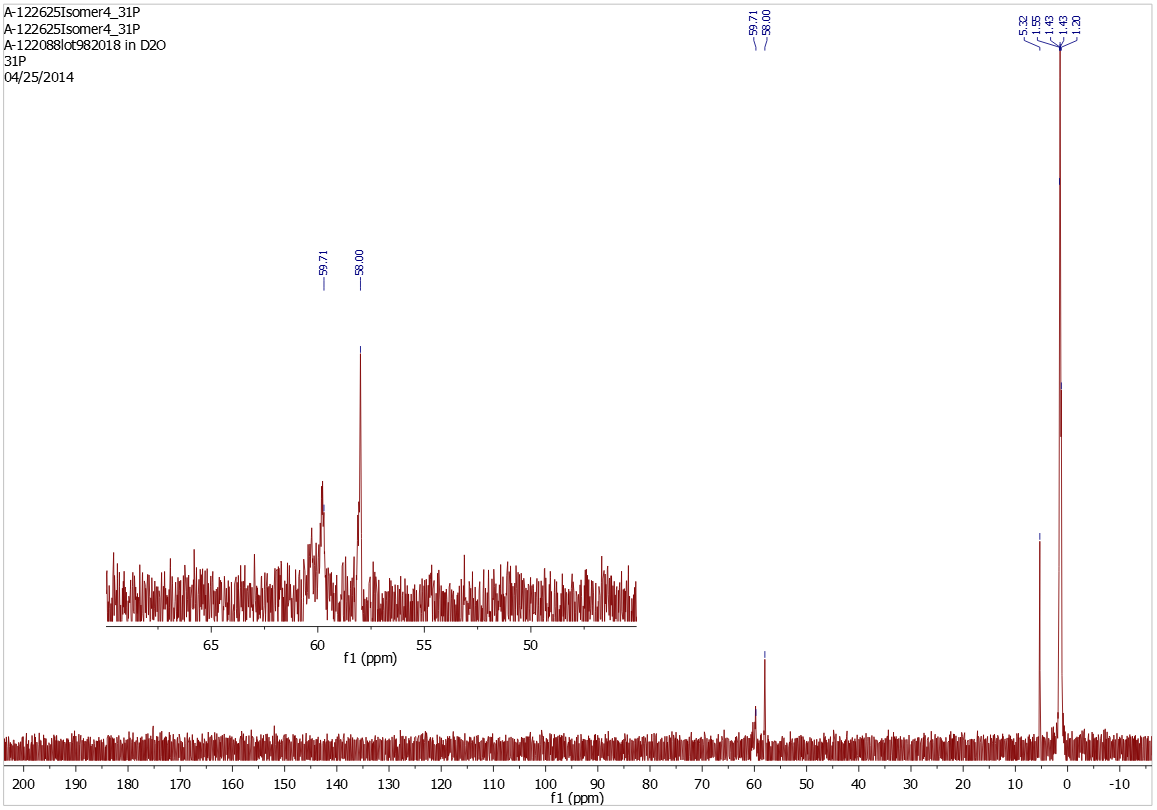


Figure S18. ^31^P-NMR spectra for si2-AS isomer 4 (5ʹR-3ʹS). Signal is very weak relative to background due to low sample concentration. However, peaks characteristic of both R_p_ (59.71 ppm) and S_p_ (58.00 ppm) are visible.


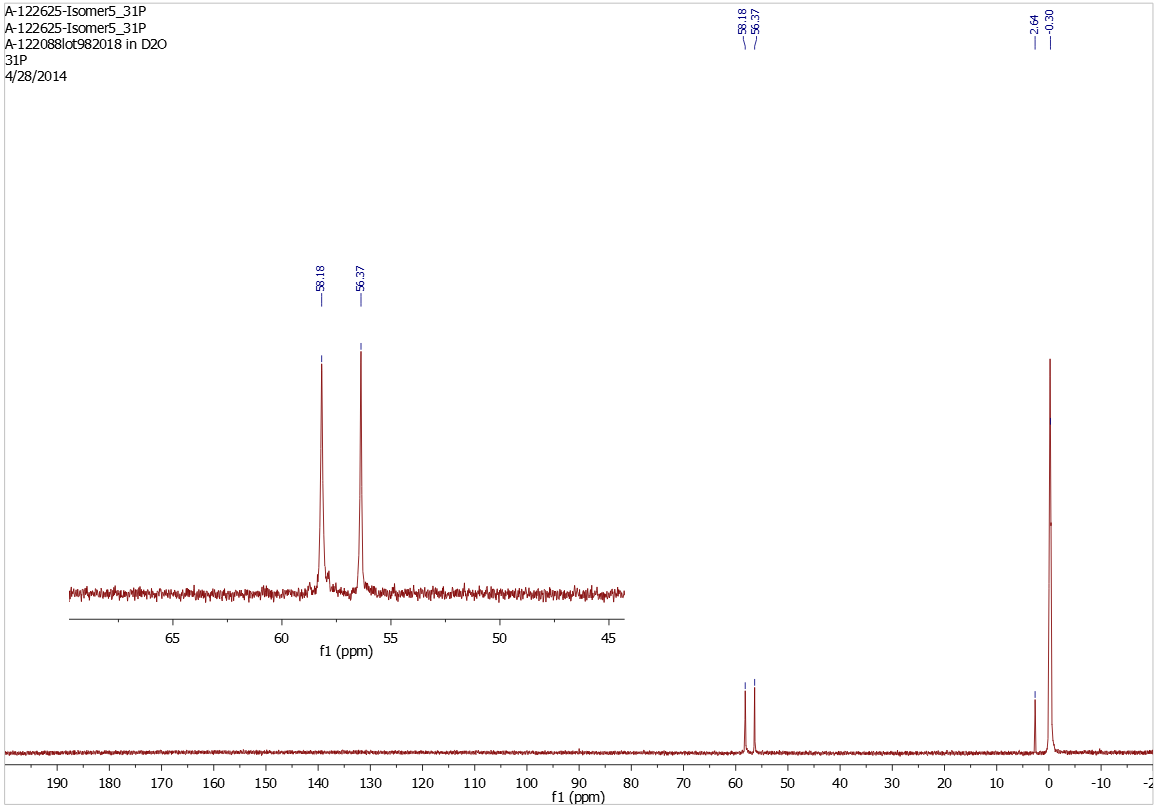


Figure S19. ^31^P-NMR spectra for si2-AS containing all four isomers. Peaks at 58.18 ppm and 56.37 ppm are due to *R*_p_ and *S*_p_ isomers, respectively. Integrating the area under the peaks, the *R*_p_:*S*_p_ ratio is approximately 1.4:1.0, consistent with the predicted synthesis ratios.

To help establish the 3ʹ-end configuration of si2-AS isomers, samples were incubated with SVPD. The analysis of the si2-AS isomers and mixture are shown in Figure S20. The half-lives of each isomer and the mixture are listed in Table S3. We consistently observed that samples containing a PS in combination with 2ʹ-OMe show little, if any, difference in stability between the *R*_p_ and *S*_p_ isomers when incubated with SVPD. In the case of si2-AS, the 3ʹ-end is 2ʹ-OMe-A in combination with the PS linkage.


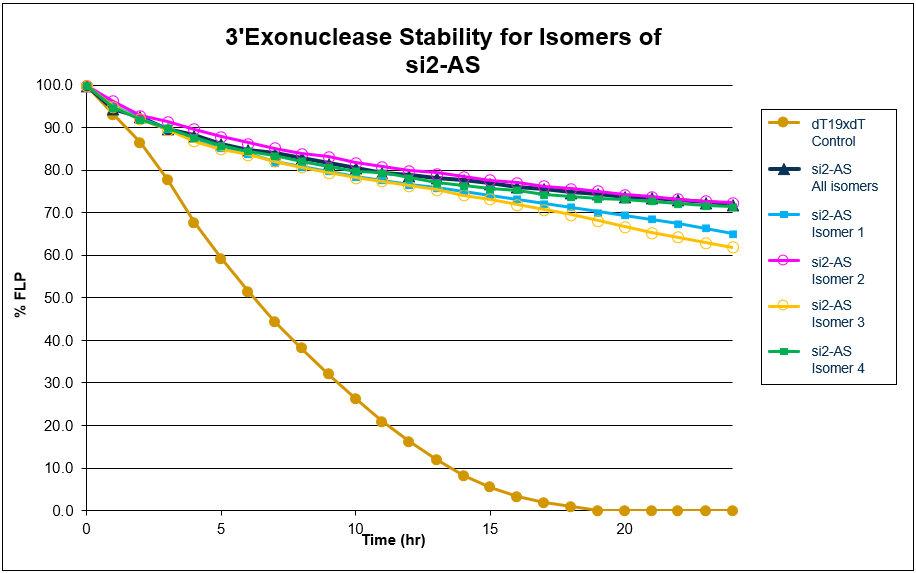


Figure S20. Full-length si2-AS isomers as a function of time in the presence of SVPD.

Table S3. Half-life of each isomer and the mixture of si2-AS isomers in the presence of SVPD.


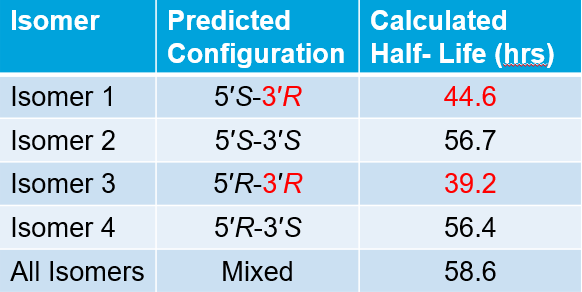


1. ***Stereo-defined dinucleotide synthesis and dinucleotide separation approach***

***Synthetic schemes, procedures, and characterization of stereo-defined dinucleotides***

## Scheme S1. Synthesis of 2ʹ-OMe uridine-2ʹ-OMe uridine phosphorothioate dinucleotide phosphoramidite building block*^a^*

^a^Reagents and conditions: (i) (a) ETT, CH_2_Cl_2_, room temperature, overnight; (b) PADS, 2,6-lutidine, room temperature, 3 h, 53% (**3a**), 41% (**3b**); (ii) Et_3_N·3HF, THF, room temperature to 50 °C, 48 h, 70% (**4a**), Et_3_N·3HF, THF, room temperature, 40 h, 66% (**4b**); (iii) 2-cyanoethyl N,N,Nʹ,Nʹ-tetraisopropylphosphorodiamidite, ETT, CH_2_Cl_2_, 0 °C to room temperature, overnight, 63% (**5a**), DIPEA, CH_2_Cl_2_, 0 °C to room temperature, 6 h, 58% (**5b**).

## Scheme S2. Synthesis of fully deprotected 2ʹ-OMe uridine-2ʹ-OMe uridine dinucleotide*^a^*

^a^Reagents and conditions: (i) 3% w/v trichloroacetic acid, CH_2_Cl_2_, room temperature, 1 h, quant. (**6a**), 90% (**6b**); (ii) 33wt% MeNH_2_, room temperature, 5 min, 70% (**7a**), 86% (**7b**).

Synthesis of compound **6a**. Compound **4a** (500 mg, 0.526 mmol) was dissolved in trichloroacetic acid (3% w/v in CH_2_Cl_2_; 8 mL). The reaction mixture was stirred at room temperature for 1 h. The reaction mixture was concentrated under vacuum and purified by column chromatography on silica gel (0–10% MeOH in CH_2_Cl_2_) to yield compound **6a** as a white foam (340 mg, quant.). ^1^H NMR (400 MHz, DMSO-*d*_6_) δ 11.44 (d, *J* = 2.1 Hz, 1H), 11.39 (d, *J* = 2.1 Hz, 1H), 7.88 (d, *J* = 8.2 Hz, 1H), 7.62 (d, *J* = 8.2 Hz, 1H), 5.93 (d, *J* = 7.0 Hz, 1H), 5.85 (d, *J* = 4.9 Hz, 1H), 5.72 (dd, *J* = 8.2, 2.1 Hz, 1H), 5.66 (d, *J* = 8.2, 2.1 Hz, 1H), 5.41 – 5.39 (m, 2H), 5.07 (ddd, *J* = 10.5, 4.8, 1.8 Hz, 1H), 4.35 – 4.04 (m, 8H), 3.83 (t, *J* = 5.1 Hz, 1H), 3.61 – 3.61 (m, 2H), 3.35 – 3.34 (m, 6H), 2.96 (t, *J* = 5.7 Hz, 2H). ^13^C NMR (126 MHz, DMSO-*d*_6_) δ 162.93, 162.84, 150.60, 150.39, 140.19, 140.05, 118.19, 102.63, 102.18, 86.77, 85.14, 83.71, 83.68, 81.94, 81.87, 81.56, 80.44, 80.40, 76.26, 76.22, 68.40, 67.70, 67.66, 63.19, 63.16, 60.59, 58.01, 57.64, 18.85, 18.78. ^31^P NMR (162 MHz, DMSO-*d*_6_) δ 67.90. HRMS calc. for C_23_H_31_N_5_O_13_PS [M + H]^+^ 648.1377, found 648.1375.

Synthesis of compound **6b**. Compound **4b** (914 mg, 0.963 mmol) was dissolved in trichloroacetic acid (3% w/v in CH_2_Cl_2_; 10 mL). The reaction mixture was stirred at room temperature for 1 h. The reaction mixture was concentrated under vacuum and purified by column chromatography on silica gel (0–10% MeOH in CH_2_Cl_2_) to yield compound **6b** as a white foam (561 mg, 90%). ^1^H NMR (400 MHz, DMSO-*d*_6_) δ 11.43 (d, *J* = 2.1 Hz, 1H), 11.39 (d, *J* = 2.1 Hz, 1H), 7.88 (d, *J* = 8.2 Hz, 1H), 7.62 (d, *J* = 8.1 Hz, 1H), 5.91 (d, *J* = 6.9 Hz, 1H), 5.84 (d, *J* = 4.7 Hz, 1H), 5.72 (dd, *J* = 8.2, 2.1 Hz, 1H), 5.65 (d, *J* = 8.1, 2.1 Hz, 1H), 5.42 – 5.39 (m, 2H), 5.06 (ddd, *J* 8.5, 4.8, 2.1 Hz, 1H), 4.32 – 4.10 (m, 7H), 4.04 – 4.01 (m, 1H), 3.83 (t, *J* = 5.0 Hz, 1H), 3.62 – 3.60 (m, 2H), 3.36 (s, 6H), 2.97 (t, *J* = 5.8 Hz, 2H). ^13^C NMR (126 MHz, DMSO-*d*_6_) δ 162.91, 162.84, 150.57, 150.35, 140.23, 140.07, 118.13, 102.57, 102.13, 86.92, 85.28, 83.68, 83.64, 81.79, 81.72, 81.59, 80.43, 80.39, 76.09, 76.05, 68.32, 67.56, 67.51, 63.31, 63.27, 60.51, 58.01, 57.68, 18.80, 18.74. ^31^P NMR (162 MHz, DMSO-*d*_6_) δ 67.99. HRMS calc. for C_23_H_30_N_5_NaO_13_PS [M + Na]^+^ 670.1196, found 670.1216.

Synthesis of compound **7a**. Compound **6a** (340 mg, 0.525 mmol) was dissolved in methylamine (33 wt% in absolute ethanol; 12 mL) and stirred at room temperature for 5 min. The reaction mixture was concentrated under vacuum and the residue was dissolved in water (2 mL). The resulting solution was treated with Dowex^®^50WX2 (Na^+^ form) resin, filtered, and concentrated under vacuum. The crude residue was purified by column chromatography on silica gel (30–40% MeOH in ethyl acetate) to yield compound **7a** as a white solid (228 mg as a sodium salt, 70%). ^1^H NMR (500 MHz, D_2_O) δ 8.12 (d, *J* = 8.2 Hz, 6H), 8.02 (d, *J* = 8.1 Hz, 1H), 6.04 (s, 1H), 5.97 (s, 1H), 5.90 (d, *J* = 8.1 Hz, 1H), 5.85 (d, *J* = 8.0 Hz, 1H), 4.91 – 4.83 (m, 1H), 4.47 (t, *J* = 4.9 Hz, 1H), 4.35 – 4.24 (m, 3H), 4.24 – 4.12 (m, 2H), 4.10 – 3.95 (m, 2H), 3.95 – 3.84 (m, 1H), 3.60 (s, 3H), 3.56 (s, 3H). ^13^C NMR (126 MHz, D_2_O) δ 165.84, 165.76, 151.22, 151.18, 141.32, 141.18, 102.30, 101.87, 87.44, 87.12, 82.93, 82.89, 82.85, 82.51, 82.43, 81.35, 81.32, 71.46, 71.42, 67.83, 63.72, 63.66, 59.45, 58.09, 57.76. ^31^P NMR (202 MHz, D_2_O) δ 56.74. HRMS calc. for C_20_H_27_N_4_NaO_13_PS [M + Na]^+^ 617.0931, found 617.0908.

Synthesis of compound **7b**. Compound **6b** (312 mg, 0.482 mmol) was dissolved in methylamine (33 wt% in absolute ethanol; 12 mL) and stirred at room temperature for 5 min. The reaction mixture was concentrated under vacuum and the residue was dissolved in water (2 mL). The resulting solution was treated with Dowex^®^50WX2 (Na^+^ form) resin, filtered, and concentrated under vacuum. The crude residue was purified by column chromatography on silica gel (30–40% MeOH in ethyl acetate) to yield compound **7b** as a white solid (255 mg as a sodium salt, 86%). ^1^H NMR (500 MHz, D_2_O) δ 8.04 (d, *J* = 8.1 Hz, 1H), 7.97 (d, *J* = 8.1 Hz, 1H), 6.04 (d, *J* = 3.3 Hz, 1H), 5.99 (d, *J* = 3.5 Hz, 1H), 5.92 – 5.88 (m, 2H), 4.88 – 4.83 (m, 1H), 4.47 (t, *J* = 5.4 Hz, 1H), 4.38 – 4.33 (m, 1H), 4.31 – 4.19 (m, 4H), 4.06 (t, *J* = 4.0 Hz, 1H), 4.00 – 3.85 (m, 2H), 3.57 (s, 3H), 3.55 (s, 3H). ^13^C NMR (126 MHz, D_2_O) δ 166.11, 166.02, 151.42, 141.34, 102.32, 102.14, 87.27, 87.15, 83.23, 83.19, 82.73, 82.70, 82.63, 81.31, 81.28, 71.42, 71.38, 67.96, 64.46, 64.41, 59.90, 58.09, 57.90. ^31^P NMR (202 MHz, D_2_O) δ 55.55. HRMS calc. for C_20_H_27_N_4_NaO_13_PS [M + Na]^+^ 617.0931, found 617.0917.

## Scheme S3. Synthesis of 2ʹ-OMe uridine-2ʹ-F uridine phosphorothioate dinucleotide phosphoramidite building block*^a^*

^a^Reagents and conditions: (i) (a) ETT, CH_2_Cl_2_, room temperature, overnight; (b) PADS, 2,6-lutidine, room temperature, 4 h, 46% (**9a**), 41% (**9b**); (ii) Et_3_N·3HF, THF, room temperature, 15–40 h, 76% (**10a**), 91% (**10b**); (iii) 2-cyanoethyl N,N,Nʹ,Nʹ-tetraisopropylphosphorodiamidite, ETT, CH_2_Cl_2_, 0 °C to room temperature, 3 h or overnight, 74% (**11a**), 76% (**11b**).

## Scheme S4. Synthesis of fully deprotected 2ʹ-OMe uridine-2ʹ-F uridine dinucleotide*^a^*

^a^Reagents and conditions: (i) 3% w/v trichloroacetic acid, CH_2_Cl_2_, room temperature, 1 h, quant. (**12a**), 92% (**12b**); (ii) 33 wt% MeNH_2_, room temperature, 5 min, 70% (**13a**), 86% (**13b**).

Synthesis of compound **12a**. Compound **10a** (500 mg, 0.533 mmol) was dissolved in trichloroacetic acid (3% w/v in CH_2_Cl_2_; 8 mL). The reaction mixture was stirred at room temperature for 1 h. The reaction mixture was concentrated under vacuum and purified by column chromatography on silica gel (5–10% MeOH in CH_2_Cl_2_) to yield compound **12a** as a white foam (338 mg, quant). ^1^H NMR (400 MHz, DMSO-*d*_6_) δ 11.45 (dd, *J* = 5.5, 2.0 Hz, 2H), 7.88 (d, *J* = 8.2 Hz, 1H), 7.61 (d, *J* = 8.1 Hz, 1H), 5.94 – 5.81 (m, 3H), 5.72 (dd, *J* = 8.1, 2.1 Hz, 1H), 5.63 (dd, *J* = 8.1, 2.1 Hz, 1H), 5.42 (t, *J* = 4.3 Hz, 1H), 5.22 – 5.03 (m, 2H), 4.47 – 4.37 (m, 1H), 4.28 – 4.02 (m, 7H), 3.61 (s, 2H), 3.34 (s, 3H), 2.95 (t, *J* = 5.8 Hz, 2H). ^13^C NMR (126 MHz, DMSO-*d*_6_) δ 163.07, 162.84, 150.59, 150.13, 140.93, 140.07, 118.17, 102.61, 101.93, 93.48, 92.00, 88.90, 88.62, 85.20, 83.67, 80.41, 80.37, 80.21, 80.14, 76.15, 76.12, 68.07, 67.93, 67.25, 67.21, 63.17, 63.14, 60.56, 57.96, 54.88, 18.83, 18.76. ^31^P NMR (202 MHz, DMSO-*d*_6_) δ 66.74. ^19^F NMR (376 MHz, DMSO-*d*_6_) δ -206.85, -206.91, -206.96, -207.00, -207.05, -207.11. HRMS calc. for C_22_H_28_FN_5_O_12_PS [M + H]^+^ 636.1177, found 636.1168.

Synthesis of compound **12b**. Compound **10b** (500 mg, 0.533 mmol) was dissolved in trichloroacetic acid (3% w/v in CH_2_Cl_2_; 10 mL) were added. The reaction mixture was stirred at room temperature for 1 h. The reaction mixture was concentrated under vacuum and purified by column chromatography on silica gel (0–10% MeOH in CH_2_Cl_2_) to yield compound **12b** as a white foam (311 mg, 92%). ^1^H NMR (400 MHz, DMSO-*d*_6_) δ 11.56 – 11.29 (m, 2H), 7.88 (d, *J* = 8.2 Hz, 1H), 7.61 (d, *J* = 8.1 Hz, 1H), 5.95 – 5.80 (m, 3H), 5.72 (dd, *J* = 8.1, 2.1 Hz, 1H), 5.62 (dd, *J* = 8.1, 2.1 Hz, 1H), 5.41 (t, *J* = 4.8 Hz, 1H), 5.23 – 5.02 (m, 2H), 4.42 – 4.32 (m, 1H), 4.30 – 4.01 (m, 7H), 3.59 (s, 2H), 3.36 (s, 3H), 2.96 (t, *J* = 5.8 Hz, 2H). ^13^C NMR (126 MHz, DMSO-*d*_6_) δ 163.55, 163.33, 151.05, 150.58, 141.50, 140.54, 118.62, 103.07, 102.37, 94.01, 92.53, 89.56, 89.27, 85.68, 84.19, 84.16, 80.92, 80.88, 80.63, 80.56, 76.58, 76.54, 68.50, 68.37, 67.60, 67.56, 63.77, 63.74, 60.99, 58.47, 55.36, 19.27, 19.20. ^31^P NMR (202 MHz, DMSO-*d*_6_) δ 66.85. ^19^F NMR (376 MHz, DMSO-*d*_6_) δ -206.40, -206.46, -206.52, -206.55, -206.60, -206.66. HRMS calc. for C_22_H_27_FN_5_NaO_12_PS [M + Na]^+^ 658.0996, found 658.0997.

Synthesis of compound **13a**. Compound **12a** (300 mg, 0.472 mmol) was dissolved in methylamine (33 wt% in absolute ethanol; 12 mL) and stirred at room temperature for 5 min. The reaction mixture was concentrated under vacuum, and the residue was dissolved in water (2 mL). The resulting solution was treated with Dowex^®^50WX2 (Na^+^ form) resin, filtered, and concentrated under vacuum. The crude residue was purified by column chromatography on silica gel (30–40% MeOH in ethyl acetate) to yield compound **13a** as a white solid (254 mg as a Na salt, 92%). ^1^H NMR (500 MHz, D_2_O) δ 8.04 – 8.00 (m, 2H), 6.12 (d, *J* = 17.7 Hz, 1H), 5.97 (m, 1H), 5.89 – 5.85(m, 2H), 5.27 – 5.09 (m, 1H), 4.87 – 4.83 (m, 1H), 4.53 – 4.46 (m, 1H), 4.44 – 4.28 (m, 3H), 4.27 – 4.14 (m, 2H), 4.06 – 3.86 (m, 2H), 3.60 (s, 3H). ^13^C NMR (126 MHz, D_2_O) δ 165.95, 151.27, 151.10, 141.38, 141.19, 102.19, 101.90, 94.13, 92.65, 88.40, 88.12, 87.41, 82.88, 82.83, 81.34, 80.84, 80.77, 71.48, 71.44, 67.51, 67.38, 63.00, 62.94, 59.48, 57.76. ^31^P NMR (202 MHz, D_2_O) δ 56.78. ^19^F NMR (376 MHz, D_2_O) δ -203.43, -203.48, -203.49, -203.54, -203.57, -203.62, -203.63, -203.68. HRMS calc. for C_19_H_24_FN_4_NaO_12_PS [M + Na]^+^ 605.0731, found 605.0718.

Synthesis of compound **13b**. Compound **12b** (292 mg, 0.482 mmol) was dissolved in methylamine (33 wt% in absolute ethanol; 12 mL) and stirred at room temperature for 5 min. The reaction mixture was concentrated under vacuum and the residue was dissolved in water (2 mL). The resulting solution was treated with Dowex^®^50WX2 (Na^+^ form) resin, filtered, and concentrated under vacuum. The crude residue was purified by column chromatography on silica gel (30–40% MeOH in ethyl acetate) to yield compound **13b** as a white solid (250 mg as a Na salt, 84.1%). ^1^H NMR (500 MHz, D_2_O) δ 8.02 – 7.88 (m, 2H), 6.09 (d, *J* = 18.4 Hz, 1H), 5.99 (d, *J* = 3.8 Hz, 1H), 5.89 (d, *J* = 8.1 Hz, 2H), 5.21 (dd, *J* = 52.3, 4.1 Hz, 1H), 4.88 – 4.84 (m, 1H), 4.55 – 4.42 (m, 1H), 4.40 – 4.29 (m, 3H), 4.29 – 4.16 (m, 2H), 4.02 – 3.81 (m, 2H), 3.57 (s, 3H). ^13^C NMR (126 MHz, D_2_O) δ 165.97, 151.34, 151.09, 141.75, 141.36, 102.16, 102.11, 94.01, 92.53, 88.82, 88.54, 87.21, 83.22, 83.18, 81.32, 81.28, 80.93, 80.85, 71.54, 71.49, 67.66, 67.53, 63.70, 63.66, 59.94, 57.89. ^31^P NMR (202 MHz, D_2_O) δ 55.68. ^19^F NMR (376 MHz, D_2_O) δ -202.94, -202.98, -202.99, -203.04, -203.07, -203.12, -203.13, -203.18. HRMS calc. for C_19_H_24_FN_4_NaO_12_PS [M + Na]^+^ 605.0731, found 605.0742.

## Scheme S5. Synthesis of 2ʹ-F uridine-2ʹ-OMe uridine phosphorothioate dinucleotide phosphoramidite building block*^a^*

^a^Reagents and conditions: (i) (a) ETT, CH_2_Cl_2_, room temperature, 2.5 h; (b) PADS, 2,6-lutidine, room temperature, overnight, 47% (**15a**), 47% (**15b**); (ii) Et_3_N·3HF, THF, room temperature, 14 h, 78% (**16a**), 76% (**16b**); (iii) 2-cyanoethyl N,N-diisopropylchlorophosphoramidite, DIPEA, CH_2_Cl_2_ or ethyl acetate, 0 °C to room temperature, 1 h, 87% (**17a**), 84% (**17b**).

## Scheme S6. Synthesis of fully deprotected 2ʹ-F uridine-2ʹ-OMe uridine dinucleotide*^a^*

^a^Reagents and conditions: (i) 3% w/v trichloroacetic acid, CH_2_Cl_2_, room temperature, 1 h, 90% (**18a**), 96% (**18b**); (ii) 33wt% MeNH_2_, room temperature, 5 min, 87% (**19a**), 86% (**19b**).

Synthesis of compound **18a**. Compound **16a** (500 mg, 0.533 mmol) was dissolved in CH_2_Cl_2_ (1.5 mL), and trichloroacetic acid (3% w/v in CH_2_Cl_2_; 8 mL) was added. The reaction mixture was stirred at room temperature for 1 h. The reaction mixture was concentrated and purified by column chromatography on silica gel (5–10% MeOH in CH_2_Cl_2_) to yield compound **18a** as a white foam (306 mg, 90%). ^1^H NMR (400 MHz, DMSO-*d*_6_) δ 11.48 (s, 1H), 11.41 (s, 1H), 7.84 (d, *J* = 8.1 Hz, 1H), 7.58 (d, *J* = 8.1 Hz, 1H), 5.96 (dd, *J* = 18.8, 3.0 Hz, 1H), 5.85 (d, *J* = 5.0 Hz, 1H), 5.67 (ddd, *J* = 7.8, 5.5, 2.1 Hz, 2H), 5.53 – 5.29 (m, 3H), 5.17 – 5.03 (m, 1H), 4.34 – 4.08 (m, 6H), 4.06 – 3.99 (m, 1H), 3.80 (t, *J* = 5.1 Hz, 1H), 3.76 – 3.66 (m, 1H), 3.60 (dt, *J* = 12.1, 4.0 Hz, 1H), 3.34 (s, 3H), 2.96 (t, *J* = 5.8 Hz, 2H). ^13^C NMR (126 MHz, DMSO-*d*_6_) δ 163.07, 162.93, 150.38, 150.26, 141.21, 140.08, 118.12, 102.19, 102.12, 91.45, 91.44, 89.94, 89.93, 88.05, 87.78, 86.62, 81.94, 81.91, 81.88, 81.84, 81.58, 73.80, 73.77, 73.69, 73.66, 68.29, 67.74, 67.70, 63.42, 63.39, 59.60, 57.63, 54.89, 18.85, 18.78. ^31^P NMR (202 MHz, DMSO-*d*_6_) δ 66.79. ^19^F NMR (376 MHz, DMSO-*d*_6_) δ -207.63, -207.67, -207.68, -207.72, -207.77, -207.81, -207.82, -207.86. HRMS calc. for C_22_H_27_FN_5_NaO_12_PS [M + Na]^+^ 658.0996, found 658.0989.

Synthesis of compound **18b**. Compound **16b** (500 mg, 0.533 mmol) was dissolved in CH_2_Cl_2_ (1.5 mL), and trichloroacetic acid (3% w/v in CH_2_Cl_2_; 8 mL) was added. The reaction mixture was stirred at room temperature for 1 h. The reaction mixture was concentrated and purified by column chromatography on silica gel (5–10% MeOH in CH_2_Cl_2_) to yield compound **18b** as a white foam (327 mg, 96%). ^1^H NMR (400 MHz, DMSO-*d*_6_) δ 11.48 (s, 1H), 11.41 (s, 1H), 7.85 (d, *J* = 8.1 Hz, 1H), 7.61 (d, *J* = 8.1 Hz, 1H), 5.95 (dd, *J* = 18.9, 2.9 Hz, 1H), 5.84 (d, *J* = 4.8 Hz, 1H), 5.66 (ddd, *J* = 14.1, 8.1, 2.1 Hz, 2H), 5.52 – 5.29 (m, 3H), 5.16 – 5.01 (m, 1H), 4.34 – 4.17 (m, 4H), 4.17 – 4.08 (m, 2H), 4.08 – 3.99 (m, 1H), 3.82 (t, *J* = 5.0 Hz, 1H), 3.76 – 3.67 (m, 1H), 3.58 (dt, *J* = 12.3, 4.3 Hz, 1H), 3.36 (s, 3H), 2.95 (t, *J* = 5.8 Hz, 2H). ^13^C NMR (126 MHz, DMSO-*d*_6_) δ 163.07, 162.93, 150.35, 150.25, 141.22, 140.23, 118.06, 102.11, 102.09, 91.48, 91.46, 89.96, 89.94, 88.13, 87.86, 86.85, 81.88, 81.82, 81.79, 81.72, 81.58, 73.58, 73.54, 73.46, 73.43, 68.27, 67.76, 67.72, 63.47, 63.44, 59.52, 57.67, 54.88, 18.81, 18.75. ^31^P NMR (202 MHz, DMSO-*d*_6_) δ 66.83. ^19^F NMR (376 MHz, DMSO-*d*_6_) δ -207.48, -207.52, -207.53, -207.57, -207.62, -207.66, -207.67, -207.71. HRMS calc. for C_22_H_27_FN_5_NaO_12_PS [M + Na]^+^ 658.0996, found 658.0984.

Synthesis of compound **19a**. Compound **18a** (286 mg, 0.450 mmol) was dissolved in methylamine (33 wt% solution in absolute ethanol; 12 mL) and was stirred at room temperature for 5 min. The reaction mixture was concentrated and purified by column chromatography on silica gel (30–40% MeOH in ethyl acetate) to yield compound **19a** as a white solid (229 mg, 87%). ^1^H NMR (500 MHz, D_2_O) δ 8.08 (d, *J* = 8.1 Hz, 1H), 7.93 (d, *J* = 8.1 Hz, 1H), 6.04 (s, 1H), 5.98 (d, *J* = 18.3 Hz, 1H), 5.92 (d, *J* = 8.1 Hz, 1H), 5.85 (d, *J* = 8.1 Hz, 1H), 5.47 – 5.21 (m, 1H), 5.00 – 4.86 (m, 1H), 4.47 (t, *J* = 5.3 Hz, 1H), 4.36 – 4.24 (m, 4H), 4.23 – 4.14 (m, 1H), 4.12 – 3.99 (m, 2H), 3.92 – 3.89 (m, 1H), 3.55 (s, 4H). ^13^C NMR (126 MHz, D_2_O) δ 165.96, 165.81, 151.24, 150.95, 141.86, 141.31, 102.37, 101.81, 92.52, 92.50, 91.02, 89.60, 89.32, 87.01, 82.83, 82.66, 82.59, 81.63, 81.56, 70.94, 70.90, 70.82, 70.78, 67.85, 63.76, 63.71, 58.93, 58.09. ^31^P NMR (202 MHz, D_2_O) δ 56.82. ^19^F NMR (376 MHz, D_2_O) δ -200.17, -200.21, -200.22, -200.27, -200.30, -200.35, -200.36, -200.41. HRMS calc. for C_19_H_24_FN_4_NaO_12_PS [M + Na]^+^ 605.0731, found 605.0727.

Synthesis of compound **19b.** Compound **18b** (307 mg, 0.483 mmol) was dissolved in methylamine (33 wt% solution in absolute ethanol; 12 mL) and was stirred at room temperature for 5 min. The reaction mixture was concentrated and purified by column chromatography on silica gel (30–40% MeOH in ethyl acetate) to yield compound **19b** as a white solid (242 mg, 86%). ^1^H NMR (400 MHz, D_2_O) δ 11.29 (s, 1H), 11.26 (s, 1H), 8.76 (s, 1H), 8.73 (s, 1H), 8.69 (s, 1H), 8.65 (s, 1H), 8.03 (d, *J* = 7.3 Hz, 4H), 7.69 – 7.59 (m, 2H), 7.59 – 7.48 (m, 4H), 6.47 (dd, *J* = 16.7, 3.6 Hz, 1H), 6.23 – 6.15 (m, 1H), 5.93 (dt, *J* = 51.4, 4.0 Hz, 1H), 5.65 – 5.57 (m, 1H), 5.54 – 5.42 (m, 1H), 5.34 (t, *J* = 5.5 Hz, 1H), 4.55 – 4.18 (m, 8H), 3.78 – 3.58 (m, 2H), 3.36 (s, 3H), 2.97 (t, *J* = 5.8 Hz, 2H). ^13^C NMR (126 MHz, D_2_O) δ 166.11, 165.94, 151.37, 151.04, 142.23, 141.30, 102.37, 101.94, 92.44, 92.42, 90.94, 90.92, 89.76, 89.47, 86.99, 82.80, 82.72, 82.70, 81.73, 81.67, 70.40, 70.35, 70.27, 70.23, 67.90, 64.59, 64.54, 59.21, 58.09. ^31^P NMR (202 MHz, D_2_O) δ 55.97. ^19^F NMR (376 MHz, D_2_O) δ -200.30, -200.35, -200.40, -200.43, -200.49, -200.54. HRMS calc. for C_19_H_24_FN_4_NaO_12_PS [M + Na]^+^ 605.0731, found 605.0737.

## Scheme S7. Synthesis of 2ʹ-OMe adenosine-2ʹ-F uridine phosphorothioate dinucleotide phosphoramidite building block*^a^*

*^a^*Reagents and conditions: (i) (a) ETT, CH_2_Cl_2_, room temperature, 2 h; (b) PADS, 2,6-lutidine, room temperature, overnight, 55% (**21a**), 37% (**21b**); (ii) Et_3_N·3HF, THF, room temperature, 14 h, 49% (**22a**), 39% (**22b**); (iii) 2-cyanoethyl *N,N*-diisopropylchlorophosphoramidite, DIPEA, EtOAc, room temperature, 1.5 h, 95% (**23a**), 91% (**23b**).

## Scheme S8. Synthesis of fully deprotected 2ʹ-OMe adenosine-2ʹ-F uridine dinucleotide*^a^*

^a^Reagents and conditions: (i) Et_3_N·3HF, THF, room temperature, 14 h (ii) 33 wt% MeNH_2_, room temperature, 2 h, 79% (**25a**), 88% (**25b**).

Synthesis of compound **25a.** Compound **24a** (800 mg, 1.05 mmol) was dissolved in methylamine (33 wt% solution in absolute ethanol; 12 mL) and stirred at room temperature for 2 h. The reaction mixture was concentrated and purified by column chromatography on silica gel (30–40% MeOH in ethyl acetate) to yield compound **25a** as a white solid (502 mg, 79%). ^1^H NMR (500 MHz, D_2_O) δ 8.22 (s, 1H), 8.02 (s, 1H), 7.68 (d, *J* = 8.1 Hz, 1H), 6.03 (d, *J* = 3.5 Hz, 1H), 5.76 (d, *J* = 17.3 Hz, 1H), 5.50 (d, *J* = 8.1 Hz, 1H), 5.02 – 4.85 (m, 2H), 4.42 (t, *J* = 3.9 Hz, 1H), 4.36 – 4.32 (m, 1H), 4.31 – 4.15 (m, 3H), 4.10 – 4.04 (m, 1H), 3.92 – 3.77 (m, 2H), 3.47 (s, 3H). ^13^C NMR (126 MHz, D_2_O) δ 165.88, 155.54, 152.65, 150.98, 148.18, 141.02, 140.08, 119.18, 101.95, 94.31, 92.83, 88.59, 88.31, 86.85, 84.03, 83.99, 81.62, 81.59, 80.98, 80.90, 72.62, 72.58, 67.61, 67.48, 63.17, 63.12, 60.42, 58.16. ^31^P NMR (202 MHz, D_2_O) δ 57.83. ^19^F NMR (376 MHz, D_2_O) δ -203.49, -203.54, -203.55, -203.60, -203.63, -203.68, -203.69, -203.74. HRMS calc. for C_20_H_26_FN_7_O_10_PS [M + H]^+^ 606.1184, found 606.1171.

Synthesis of compound **25b.** Compound **24b** (400 mg, 0.525 mmol) was dissolved methylamine (33 wt% solution in absolute ethanol; 12 mL) and stirred at room temperature for 2 h. The reaction mixture was concentrated and purified by column chromatography on silica gel (30–40% MeOH in ethyl acetate) to yield compound **25b** as a white solid (282 mg, 88%). ^1^H NMR (500 MHz, D_2_O) δ 8.19 (s, 1H), 8.04 (s, 1H), 7.67 (d, *J* = 8.1 Hz, 1H), 5.99 (d, *J* = 5.3 Hz, 1H), 5.83 (d, *J* = 18.3 Hz, 1H), 5.57 (d, *J* = 8.1 Hz, 1H), 5.02 (dd, *J* = 52.4, 4.2 Hz, 1H), 4.93 – 4.87 (m, 1H), 4.45 (t, *J* = 5.0 Hz, 1H), 4.41 – 4.37 (m, 1H), 4.36 – 4.27 (m, 1H), 4.25 – 4.08 (m, 3H), 3.84 – 3.72 (m, 2H), 3.40 (s, 3H). ^13^C NMR (126 MHz, D_2_O) δ 165.90, 155.61, 152.59, 151.07, 148.35, 141.64, 140.44, 119.25, 102.05, 94.19, 92.72, 89.06, 88.78, 86.84, 84.66, 84.63, 81.55, 81.51, 81.08, 81.01, 72.83, 72.78, 67.83, 67.70, 63.91, 63.87, 60.95, 58.21. ^31^P NMR (202 MHz, D_2_O) δ 56.63. ^19^F NMR (376 MHz, D_2_O) δ -202.83, -202.88, -202.94, -202.97, -203.02, -203.08. HRMS calc. for C_20_H_26_FN_7_O_10_PS [M + H]^+^ 606.1184, found 606.1183.

## Scheme S9. Synthesis of 2ʹ-OMe adenosine-2ʹ-OMe adenosine phosphorothioate dinucleotide phosphoramidite building block*^a^*

^a^Reagents and conditions: (i) (a) ETT, CH_2_Cl_2_, room temperature, 1.5 h; (b) PADS, 2,6-lutidine, room temperature, overnight, 60% (**27a**), 35% (**27b**); (ii) Et_3_N·3HF, THF, room temperature, 14 h, 75% (**28a**), 52% (**28b**); (iii) 2-cyanoethyl N,N-diisopropylchlorophosphoramidite, DIPEA, CH_2_Cl_2_, room temperature, 1.5 h, 63% (**29a**), 55% (**29b**).

## Scheme S10. Synthesis of fully deprotected 2ʹ-OMe adenosine-2ʹ-OMe adenosinedinucleotide*^a^*

^a^Reagents and conditions: (i) Et_3_N·3HF, THF, room temperature, 14 h ii) 33wt% MeNH_2_, room temperature, 4 h, 99% (**31a**), 37% (**31b**).

Compound **30a**. ^1^H NMR (400 MHz, DMSO-*d*_6_) δ 11.25 (s, 1H), 11.23 (s, 1H), 8.78 (s, 1H), 8.76 (s, 1H), 8.73 (s, 1H), 8.68 (s, 1H), 8.04 (d, *J* = 7.5 Hz, 4H), 7.71 – 7.59 (m, 2H), 7.59 – 7.47 (m, 4H), 6.25 – 6.18 (m, 2H), 5.60 (d, *J* = 4.9 Hz, 1H), 5.42 (t, *J* = 5.6 Hz, 1H), 5.29 (dd, *J* = 10.9, 4.8 Hz, 1H), 4.88 – 4.80 (m, 1H), 4.57 – 4.45 (m, 3H), 4.42 – 4.31 (m, 2H), 4.30 – 4.20 (m, 3H), 3.71 – 3.61 (m, 2H), 3.38 (s, 3H), 3.33 (s, 3H), 2.98 (t, *J* = 5.9 Hz, 2H). ^13^C NMR (126 MHz, DMSO-*d*_6_) δ 165.62, 152.11, 151.99, 151.83, 150.63, 150.51, 142.94, 133.25, 133.22, 132.47, 132.43, 128.48, 128.44, 128.42, 125.80, 125.75, 118.17, 85.88, 85.06, 84.71, 82.84, 82.77, 81.75, 80.52, 80.49, 76.42, 76.39, 68.73, 67.91, 67.86, 63.18, 63.15, 60.86, 58.03, 57.73, 54.88, 18.84, 18.77. ^31^P NMR (202 MHz, DMSO-*d*_6_) δ 67.94. HRMS calc. for C_39_H_40_N_11_NaO_11_PS [M + Na]^+^ 924.2266, found 924.2268.

Compound **30b**. ^1^H NMR (400 MHz, DMSO-*d*_6_) δ 11.25 (s, 1H), 11.22 (s, 1H), 8.78 (s, 1H), 8.76 (s, 1H), 8.75 (s, 1H), 8.68 (s, 1H), 8.04 (dd, *J* = 5.8, 3.9 Hz, 4H), 7.69 – 7.59 (m, 2H), 7.59 – 7.49 (m, 4H), 6.22 (d, *J* = 4.0 Hz, 1H), 6.18 (d, *J* = 7.2 Hz, 1H), 5.61 (d, *J* = 4.9 Hz, 1H), 5.42 (t, *J* = 5.7 Hz, 1H), 5.29 (ddd, *J* = 10.9, 4.8, 1.9 Hz, 1H), 4.91 – 4.76 (m, 1H), 4.59 – 4.48 (m, 2H), 4.47 – 4.34 (m, 2H), 4.34 – 4.29 (m, 1H), 4.29 – 4.21 (m, 3H), 3.77 – 3.56 (m, 2H), 3.41 (s, 3H), 3.37 (s, 3H), 2.96 (t, *J* = 5.9 Hz, 2H). ^13^C NMR (126 MHz, DMSO-*d*_6_) δ 165.61, 152.11, 151.96, 151.83, 150.63, 150.51, 142.99, 142.93, 133.26, 133.22, 132.47, 132.42, 128.47, 128.44, 128.41, 125.80, 125.76, 118.13, 85.95, 85.09, 84.68, 82.76, 82.69, 81.70, 80.49, 76.32, 68.70, 67.85, 67.82, 63.28, 63.25, 60.81, 58.06, 57.78, 18.80, 18.73. ^31^P NMR (202 MHz, DMSO-*d*_6_) δ 68.05. HRMS calc. for C_39_H_40_N_11_NaO_11_PS [M + Na]^+^ 924.2266, found 924.2268.

Synthesis of compound **31a.** Compound **30a** (1.90 g, 2.14 mmol) was dissolved methylamine (33 wt% solution in absolute ethanol; 12 mL) and stirred at room temperature for 4 h. The reaction mixture was concentrated and purified by column chromatography on silica gel (30–40% MeOH in ethyl acetate) to yield compound **31a** as a white solid (1.20 g, 99%). ^1^H NMR (500 MHz, D_2_O) δ 8.41 (s, 1H), 8.22 (s, 1H), 8.11 (s, 1H), 7.92 (s, 1H), 6.04 (d, *J* = 4.4 Hz, 1H), 5.99 (d, *J* = 3.9 Hz, 1H), 5.05 – 4.99 (m, 1H), 4.63 (t, *J* = 5.0 Hz, 1H), 4.42 – 4.35 (m, 3H), 4.31 – 4.19 (m, 3H), 3.98 – 3.85 (m, 2H), 3.52 (s, 3H), 3.49 (s, 3H). ^13^C NMR (126 MHz, D_2_O) δ 157.75, 157.50, 155.31, 154.76, 150.78, 150.34, 142.44, 141.89, 121.29, 120.85, 89.31, 88.42, 86.39, 86.20, 86.01, 84.02, 75.23, 71.37, 67.00, 63.01, 60.98, 60.47. ^31^P NMR (202 MHz, D_2_O) δ 57.51. HRMS calc. for C_22_H_30_N_10_O_9_PS [M + H]^+^ 641.1656, found 641.1668.

Synthesis of compound **31b**. Compound **30b** (840 mg, 0.944 mmol) was dissolved methylamine (33 wt% solution in absolute ethanol; 12 mL) and stirred at room temperature for 4 h. The reaction mixture was concentrated and purified by column chromatography on silica gel (30–40% MeOH in ethyl acetate) to yield compound **31b** as a white solid (210 mg, 37%). ^1^H NMR (500 MHz, D_2_O) δ 8.38 (s, 1H), 8.20 (s, 1H), 8.10 (s, 1H), 7.97 (s, 1H), 6.08 (d, *J* = 4.6 Hz, 1H), 5.91 (d, *J* = 5.4 Hz, 1H), 5.04 – 4.98 (m, 1H), 4.67 (t, *J* = 4.7 Hz, 1H), 4.43 (t, *J* = 5.0 Hz, 1H), 4.41 – 4.37 (m, 2H), 4.37 – 4.33 (m, 1H), 4.26 – 4.20 (m, 2H), 3.83 – 3.74 (m, 2H), 3.50 (s, 3H), 3.46 (s, 3H). ^13^C NMR (126 MHz, D_2_O) δ 157.74, 155.22, 154.74, 151.11, 150.55, 142.85, 141.96, 121.48, 120.97, 89.24, 88.19, 86.99, 86.26, 86.18, 85.74, 83.92, 75.42, 71.50, 67.87, 63.51, 60.91, 60.54. ^31^P NMR (202 MHz, D_2_O) δ 56.27. HRMS calc. for C_22_H_30_N_10_O_9_PS [M + H]^+^ 641.1656, found 641.1655.

## Scheme S11. Synthesis of 2ʹ-F adenosine-2ʹ-OMe adenosine phosphorothioate dinucleotide phosphoramidite building block*^a^*

^a^Reagents and conditions: (i) (a) ETT, CH_2_Cl_2_, room temperature, 1.5 h; (b) PADS, 2,6-lutidine, room temperature, overnight, 44% (**33a**), 41% (**33b**); (ii) Et_3_N·3HF, THF, room temperature, 14 h, 90% (**34a**), 73% (**34b**); (iii) 2-cyanoethyl N,N-diisopropylchlorophosphoramidite, DIPEA, EtOAc, room temperature, 1.5 h, 86% (**35a**), 90% (**35b**).

## Scheme S12. Synthesis of fully deprotected 2ʹ-F adenosine-2ʹ-OMe adenosine dinucleotide*^a^*

^a^Reagents and conditions: (i) 3% w/v trichloroacetic acid, CH_2_Cl_2_, room temperature, 1 h, 95% (**36a**), 88% (**36b**); (ii) 33 wt% MeNH_2_, room temperature, 2 h, 85% (**37a**), 85% (**37b**).

Synthesis of compound **36a.** Compound **34a** (500 mg, 0.419 mmol) was dissolved in CH_2_Cl_2_ (1.5 mL) and trichloroacetic acid (3% w/v in CH_2_Cl_2_; 8 mL) was added. The reaction mixture was stirred at room temperature for 1 h. The reaction mixture was concentrated and purified by column chromatography on silica gel (0–5% MeOH in CH_2_Cl_2_) to yield compound **36a** as a white foam (355 mg, 95%). ^1^H NMR (400 MHz, DMSO-*d*_6_) δ 11.29 (s, 1H), 11.26 (s, 1H), 8.76 (s, 1H), 8.73 (s, 1H), 8.69 (s, 1H), 8.65 (s, 1H), 8.03 (d, *J* = 7.3 Hz, 4H), 7.69 – 7.59 (m, 2H), 7.59 – 7.48 (m, 4H), 6.47 (dd, *J* = 16.7, 3.6 Hz, 1H), 6.23 – 6.15 (m, 1H), 5.93 (dt, *J* = 51.4, 4.0 Hz, 1H), 5.65 – 5.57 (m, 1H), 5.54 – 5.42 (m, 1H), 5.34 (t, *J* = 5.5 Hz, 1H), 4.55 – 4.18 (m, 8H), 3.78 – 3.58 (m, 2H), 3.36 (s, 3H), 2.97 (t, *J* = 5.8 Hz, 2H). ^13^C NMR (126 MHz, DMSO-*d*_6_) δ 165.62, 165.59, 151.97, 151.82, 151.80, 151.72, 150.58, 150.49, 142.98, 142.87, 133.25, 133.20, 132.49, 132.45, 128.48, 128.45, 128.44, 125.76, 125.74, 118.10, 91.51, 91.49, 89.96, 85.82, 85.72, 85.46, 82.80, 82.73, 82.67, 81.73, 74.36, 74.33, 74.25, 74.22, 68.67, 68.03, 67.99, 63.42, 63.39, 59.89, 57.73, 54.88, 18.84, 18.77. ^31^P NMR (202 MHz, DMSO-*d*_6_) δ 66.78. ^19^F NMR (376 MHz, DMSO-*d*_6_) δ -210.64, -210.67, -210.68, -210.72, -210.78, -210.81, -210.82, -210.85. HRMS calc. for _38_H_37_FN_11_NaO_10_PS [M + Na]^+^ 912.2065, found 912.2079.

Synthesis of compound **36b**. Compound **34b** (500 mg, 0.419 mmol) was dissolved in CH_2_Cl_2_ (1.5 mL), and trichloroacetic acid (3% w/v in CH_2_Cl_2_; 8 mL) was added. The reaction mixture was stirred at room temperature for 1 h. The reaction mixture was concentrated and purified by column chromatography on silica gel (0–5% MeOH in CH_2_Cl_2_) to yield compound **36b** as a white foam (327 mg, 88%). ^1^H NMR (400 MHz, DMSO-*d*_6_) δ 11.27 (d, *J* = 13.1 Hz, 2H), 8.77 (d, *J* = 5.5 Hz, 1H), 8.68 (d, *J* = 8.1 Hz, 2H), 8.03 (d, *J* = 8.0 Hz, 4H), 7.68 – 7.49 (m, 6H), 6.47 (dd, *J* = 16.9, 3.5 Hz, 1H), 6.21 (d, *J* = 4.4 Hz, 1H), 5.93 (dt, *J* = 51.5, 3.9 Hz, 1H), 5.62 (d, *J* = 5.3 Hz, 1H), 5.54 – 5.43 (m, 1H), 5.32 (t, *J* = 5.5 Hz, 1H), 4.55 – 4.48 (m, 2H), 4.48 – 4.33 (m, 2H), 4.31 – 4.18 (m, 4H), 3.78 – 3.56 (m, 2H), 3.39 (s, 3H), 2.93 (t, *J* = 5.8 Hz, 2H). ^13^C NMR (126 MHz, DMSO-*d*_6_) δ 165.61, 165.59, 151.96, 151.83, 151.82, 151.71, 150.59, 150.51, 143.00, 142.97, 133.25, 133.20, 132.49, 132.45, 128.48, 128.45, 125.79, 125.76, 118.03, 91.53, 91.51, 90.00, 89.98, 85.92, 85.78, 85.51, 82.76, 82.70, 82.65, 82.60, 81.71, 74.16, 74.13, 68.68, 68.13, 68.09, 63.46, 63.42, 59.81, 57.77, 54.89, 18.79, 18.72. ^31^P NMR (202 MHz, DMSO-*d*_6_) δ 66.88. ^19^F NMR (376 MHz, DMSO-*d*_6_) δ -210.45, -210.48, -210.49, -210.53, -210.58, -210.62, -210.63, -210.66. HRMS calc. for C_38_H_37_FN_11_NaO_10_PS [M + Na]^+^ 912.2065, found 912.2053.

Synthesis of compound **37a**. Compound **36a** (340 mg, 0.382 mmol) was dissolved methylamine (33 wt% solution in absolute ethanol; 12 mL) and stirred at room temperature for 2 h. The reaction mixture was concentrated and purified by column chromatography on silica gel (30–40% MeOH in ethyl acetate) to yield compound **37a** as a white solid (205 mg, 85%). ^1^H NMR (500 MHz, D_2_O) δ 8.39 (s, 1H), 8.23 (s, 1H), 8.14 (s, 1H), 7.83 (s, 1H), 6.15 (d, *J* = 16.1 Hz, 1H), 6.00 (d, *J* = 3.6 Hz, 1H), 5.41 (dd, 1H), 5.14 – 5.01 (m, 1H), 4.61 (t, *J* = 5.1 Hz, 1H), 4.46 – 4.40 (m, 1H), 4.40 – 4.32 (m, 2H), 4.24 – 4.19 (m, 1H), 4.14 (d, *J* = 13.1 Hz, 1H), 4.11 – 4.05 (m, 1H), 3.96 (dd, *J* = 13.2, 3.6 Hz, 1H), 3.48 (s, 3H). ^13^C NMR (126 MHz, D_2_O) δ 154.90, 154.50, 152.67, 151.81, 147.56, 146.83, 138.86, 138.69, 118.31, 117.86, 92.36, 90.86, 87.70, 87.44, 85.89, 83.97, 82.90, 82.82, 81.63, 81.56, 71.31, 71.27, 71.19, 71.15, 68.23, 63.34, 63.29, 59.01, 58.33. ^31^P NMR (202 MHz, D_2_O) δ 57.21. ^19^F NMR (376 MHz, D_2_O) δ -200.83, -200.87, -200.89, -200.93, -200.96, -201.01, -201.03, -201.07. HRMS calc. for C_21_H_27_FN_10_O_8_PS [M + H]^+^ 629.1456, found 629.1449.

Synthesis of compound **37b.** Compound **36b** (312 mg, 0.351 mmol) was dissolved methylamine (33 wt% solution in absolute ethanol; 12 mL) and stirred at room temperature for 2 h. The reaction mixture was concentrated and purified by column chromatography on silica gel (30–40% MeOH in ethyl acetate) to yield compound **37b** as a white solid (186 mg, 85%). ^1^H NMR (500 MHz, D_2_O) δ 8.28 (s, 2H), 8.22 (s, 1H), 8.13 (s, 13H), 7.86 (s, 1H), 6.14 (d, *J* = 15.9 Hz, 1H), 6.06 – 5.98 (m, 1H), 5.46 (d, *J* = 52.3 Hz, 1H), 5.00 (dtd, *J* = 18.9, 9.1, 4.1 Hz, 1H), 4.63 (t, *J* = 4.7 Hz, 1H), 4.41 – 4.34 (m, 2H), 4.28 (s, 2H), 4.18 (s, 1H), 4.07 – 4.01 (m, 1H), 3.94 – 3.88 (m, 1H), 3.48 (s, 4H). ^13^C NMR (126 MHz, D_2_O) δ 154.97, 154.67, 152.67, 151.92, 147.82, 147.11, 139.16, 138.56, 118.38, 117.96, 92.16, 90.64, 87.31, 87.04, 85.65, 83.56, 83.12, 83.04, 82.12, 82.06, 70.37, 70.31, 70.25, 70.19, 68.23, 64.81, 64.80, 64.77, 59.24, 58.25. ^31^P NMR (202 MHz, D_2_O) δ 55.40. ^19^F NMR (376 MHz, D_2_O) δ -202.19, -202.23, -202.24, -202.28, -202.32, -202.37, -202.38, -202.42. HRMS calc. for C_21_H_27_FN_10_O_8_PS [M + H]^+^ 629.1456, found 629.1452.

## Scheme S13. Synthesis of 2ʹ-OMe guanosine-2ʹ-OMe adenosine phosphorothioate dinucleotide phosphoramidite building block*^a^*

^a^Reagents and conditions: (i) (a) ETT, CH_2_Cl_2_, room temperature, 2 h; (b) PADS, 2,6-lutidine, room temperature, overnight, 86%; (ii) Et_3_N·3HF, THF, room temperature, 14 h, 90% (mixture of **40a** and **40b**); (iii) 2-cyanoethyl N,N-diisopropylchlorophosphoramidite, DIPEA, EtOAc, room temperature, 1.5 h, 94% (**41a**), 98% (**41b**).

## Scheme S14. Synthesis of fully deprotected 2ʹ-OMe guanosine-2ʹ-OMe adenosinedinucleotide*^a^*

^a^Reagents and conditions: (i) 3% w/v trichloroacetic acid, CH_2_Cl_2_, room temperature, 1.5 h, 94% (**42a**), 92% (**42b**); (ii) 33 wt% MeNH_2_, room temperature, 3 h, 83% (**43a**), 91% (**43b**).

Synthesis of compound **42a**. Compound **40a** (531 mg, 0.448 mmol) was dissolved in CH_2_Cl_2_ (1.5 mL), and trichloroacetic acid (3% w/v in CH_2_Cl_2_; 17 mL) were added. The reaction mixture was stirred at room temperature for 1.5 h. The reaction mixture was concentrated and purified by column chromatography on silica gel (0–5% MeOH in CH_2_Cl_2_) to yield compound **42a** as a white foam (373 mg, 94%). ^1^H NMR (400 MHz, DMSO-*d*_6_) δ 12.07 (s, 1H), 11.67 (s, 1H), 11.22 (s, 1H), 8.77 (s, 1H), 8.67 (s, 1H), 8.30 (s, 1H), 8.03 (d, *J* = 7.4 Hz, 2H), 7.64 (t, *J* = 7.4 Hz, 1H), 7.54 (t, *J* = 7.6 Hz, 2H), 6.20 (d, *J* = 4.1 Hz, 1H), 5.91 – 5.85 (m, 1H), 5.61 (d, *J* = 5.5 Hz, 1H), 5.37 (t, *J* = 5.3 Hz, 1H), 5.19 (dd, *J* = 10.5, 4.6 Hz, 1H), 4.61 (dt, *J* = 7.7, 3.4 Hz, 1H), 4.54 – 4.46 (m, 2H), 4.37 (ddt, *J* = 18.7, 11.1, 5.9 Hz, 2H), 4.27 – 4.15 (m, 4H), 3.59 (d, *J* = 4.5 Hz, 2H), 3.40 (s, 3H), 3.32 (s, 3H), 2.93 (t, *J* = 5.8 Hz, 2H), 2.77 – 2.67 (m, 1H), 1.10 (d, *J* = 6.8 Hz, 6H). ^13^C NMR (126 MHz, DMSO-*d*_6_) δ 180.06, 165.61, 164.38, 154.71, 151.92, 151.84, 150.52, 149.01, 148.45, 143.00, 137.10, 133.22, 132.46, 128.46, 128.43, 125.80, 119.97, 118.17, 86.00, 84.65, 84.64, 83.66, 82.71, 82.64, 81.70, 80.83, 80.79, 76.70, 76.66, 71.06, 68.70, 67.95, 67.91, 63.27, 63.24, 60.72, 58.01, 57.81, 34.76, 18.81, 18.77, 18.73. ^31^P NMR (202 MHz, DMSO-*d*_6_) δ 68.30. HRMS calc. for C_36_H_43_N_11_O_12_PS [M + H]^+^ 884.2551, found 884.2556.

Synthesis of compound **42b.** Compound **40b** (517 mg, 0.436 mmol) was dissolved in CH_2_Cl_2_ (1.5 mL), and trichloroacetic acid (3% w/v in CH_2_Cl_2_; 17 mL) were added. The reaction mixture was stirred at room temperature for 1.5 h. The reaction mixture was concentrated and purified by column chromatography on silica gel (0–5% MeOH in CH_2_Cl_2_) to yield compound **42b** as a white foam (365 mg, 92%). ^1^H NMR (400 MHz, DMSO-*d*_6_) δ 12.06 (s, 1H), 11.63 (s, 1H), 11.20 (s, 1H), 8.75 (s, 1H), 8.66 (s, 1H), 8.29 (s, 1H), 8.02 (d, *J* = 7.3 Hz, 2H), 7.64 (t, *J* = 7.4 Hz, 1H), 7.54 (t, *J* = 7.6 Hz, 2H), 6.20 (d, *J* = 4.1 Hz, 1H), 5.88 (d, *J* = 7.9 Hz, 1H), 5.62 – 5.55 (m, 1H), 5.34 (t, *J* = 5.2 Hz, 1H), 5.17 (dd, *J* = 10.5, 4.6 Hz, 1H), 4.63 – 4.55 (m, 1H), 4.53 – 4.41 (m, 3H), 4.37 – 4.29 (m, 1H), 4.27 – 4.16 (m, 4H), 3.62 – 3.50 (m, 2H), 3.39 (s, 3H), 3.27 (s, 3H), 2.96 (t, *J* = 5.8 Hz, 2H), 2.76 – 2.66 (m, 1H), 1.09 (d, *J* = 6.7 Hz, 6H). ^13^C NMR (126 MHz, DMSO-*d*_6_) δ 180.05, 165.59, 154.70, 151.89, 151.81, 150.51, 148.99, 148.43, 143.00, 137.08, 133.21, 132.47, 128.47, 128.43, 125.78, 119.96, 118.23, 86.01, 84.63, 83.65, 82.69, 82.62, 81.73, 80.88, 80.84, 76.72, 76.68, 68.78, 68.20, 68.16, 63.15, 63.12, 60.72, 57.94, 57.80, 34.76, 18.84, 18.80, 18.76. ^31^P NMR (202 MHz, DMSO-*d*_6_) δ 68.19. HRMS calc. for C_36_H_43_N_11_O_12_PS [M + H]^+^ 884.2551, found 884.2552.

Synthesis of compound **43a.** Compound **42a** (358 mg, 0.405 mmol) was dissolved methylamine (33 wt% solution in absolute ethanol; 17 mL) and stirred at room temperature for 3 h. The reaction mixture was concentrated and purified by column chromatography on silica gel (30–40% MeOH in ethyl acetate) to yield compound **43a** as a white solid (222 mg, 83%). ^1^H NMR (400 MHz, D_2_O) δ 8.41 (s, 1H), 8.17 (s, 1H), 7.92 (s, 1H), 6.14 (d, *J* = 4.9 Hz, 1H), 5.75 (d, *J* = 5.1 Hz, 1H), 5.04 – 4.95 (m, 1H), 4.68 (t, *J* = 4.8 Hz, 1H), 4.45 – 4.34 (m, 3H), 4.34 – 4.27 (m, 1H), 4.27 – 4.19 (m, 2H), 3.79 (d, *J* = 3.1 Hz, 2H), 3.49 (d, *J* = 8.3 Hz, 6H), 3.36 (s, 1H), 2.61 (s, 2H). ^13^C NMR (126 MHz, D_2_O) δ 158.74, 155.56, 153.59, 153.01, 151.16, 148.88, 139.66, 137.71, 118.69, 116.69, 86.17, 85.81, 84.15, 84.12, 83.94, 83.86, 83.38, 81.37, 81.33, 72.76, 72.71, 69.11, 65.61, 65.56, 61.07, 58.55, 58.10. ^31^P NMR (202 MHz, D_2_O) δ 56.07. HRMS calc. for C_22_H_30_N_10_O_10_PS [M + H]^+^ calculated 657.1605, found 657.1580.

Synthesis of compound **43b.** Compound **42b** (350 mg, 0.396 mmol) was dissolved methylamine (33 wt% solution in absolute ethanol; 12 mL) and stirred at room temperature for 2 h. The reaction mixture was concentrated and purified by column chromatography on silica gel (30–40% MeOH in ethyl acetate) to yield compound **43b** as a white solid (240 mg, 91%). ^1^H NMR (400 MHz, D_2_O) δ 8.46 (s, 1H), 8.17 (s, 1H), 7.90 (s, 1H), 6.13 (d, *J* = 4.4 Hz, 1H), 5.80 (d, *J* = 3.2 Hz, 1H), 5.04 (dt, *J* = 11.4, 5.6 Hz, 1H), 4.65 (t, *J* = 4.9 Hz, 1H), 4.43 – 4.37 (m, 1H), 4.35 – 4.25 (m, 4H), 4.25 – 4.16 (m, 1H), 3.98 – 3.80 (m, 2H), 3.51 (d, *J* = 9.7 Hz, 6H), 2.61 (s, 2H). ^13^C NMR (126 MHz, D_2_O) δ 158.51, 155.51, 153.42, 153.00, 150.82, 148.59, 139.64, 137.36, 118.60, 116.59, 86.68, 86.08, 83.83, 83.71, 83.63, 83.57, 83.53, 81.43, 81.41, 72.79, 72.75, 69.08, 64.67, 64.61, 60.68, 58.63, 58.00. ^31^P NMR (202 MHz, D_2_O) δ 57.53. HRMS calc. for C_22_H_30_N_10_O_10_PS [M + H]^+^ calculated 657.1605, found 657.1583.

***^1^H, ^13^C, ^19^F, and ^31^P NMR spectra for stereo-defined dinucleotides***

^1^H NMR spectrum of compound **3a** in DMSO-*d*_6_

^13^C NMR spectrum of compound **3a** in DMSO-*d*_6_

^31^P NMR spectrum of compound **3a** in DMSO-*d*_6_

^1^H NMR spectrum of compound **3b** in DMSO-*d*_6_

^13^C NMR spectrum of compound **3b** in DMSO-*d*_6_

^31^P NMR spectrum of compound **3b** in DMSO-*d*_6_

^1^H NMR spectrum of compound **4a** in DMSO-*d*_6_

^13^C NMR spectrum of compound **4a** in DMSO-*d*_6_

^31^P NMR spectrum of compound **4a** in DMSO-*d*_6_

^1^H NMR spectrum of compound **4b** in DMSO-*d*_6_

^13^C NMR spectrum of compound **4b** in DMSO-*d*_6_

^31^P NMR spectrum of compound **4b** in DMSO-*d*_6_

^1^H NMR spectrum of compound **5a** in CD_3_CN

^13^C NMR spectrum of compound **5a** in CD_3_CN

^31^P NMR spectrum of compound **5a** in CD_3_CN

^1^H NMR spectrum of compound **5b** in CD_3_CN

^13^C NMR spectrum of compound **5b** in CD_3_CN

^31^P NMR spectrum of compound **5b** in CD_3_CN

^1^H NMR spectrum of compound **6a** in DMSO-*d*_6_

^13^C NMR spectrum of compound **6a** in DMSO-*d*_6_

^31^P NMR spectrum of compound **6a** in DMSO-*d*_6_

^1^H NMR spectrum of compound **6b** in DMSO-*d*_6_

^13^C NMR spectrum of compound **6b** in DMSO-*d*_6_

^31^P NMR spectrum of compound **6b** in DMSO-*d*_6_

^1^H NMR spectrum of compound **7a** in D_2_O

^13^C NMR spectrum of compound **7a** in D_2_O

^31^P NMR spectrum of compound **7a** in D_2_O

^1^H NMR spectrum of compound **7b** in D_2_O

^13^C NMR spectrum of compound **7b** in D_2_O

^31^P NMR spectrum of compound **7b** in D_2_O

^1^H NMR spectrum of compound **9a** in DMSO-*d*_6_

^13^C NMR spectrum of compound **9a** in DMSO-*d*_6_

^19^F NMR spectrum of compound **9a** in DMSO-*d*_6_

^31^P NMR spectrum of compound **9a** in DMSO-*d*_6_

^1^H NMR spectrum of compound **9b** in DMSO-*d*_6_

^13^C NMR spectrum of compound **9b** in DMSO-*d*_6_

^19^F NMR spectrum of compound **9b** in DMSO-*d*_6_

^31^P NMR spectrum of compound **9b** in DMSO-*d*_6_

^1^H NMR spectrum of compound **10a** in DMSO-*d*_6_

^13^C NMR spectrum of compound **10a** in DMSO-*d*_6_

^19^F NMR spectrum of compound **10a** in DMSO-*d*_6_

^31^P NMR spectrum of compound **10a** in DMSO-*d*_6_

^1^H NMR spectrum of compound **10b** in DMSO-*d*_6_

^13^C NMR spectrum of compound **10b** in DMSO-*d*_6_

^19^F NMR spectrum of compound **10b** in DMSO-*d*_6_

^31^P NMR spectrum of compound **10b** in DMSO-*d*_6_

^1^H NMR spectrum of compound **11a** in CD_3_CN

^13^C NMR spectrum of compound **11a** in CD_3_CN

^19^F NMR spectrum of compound **11a** in CD_3_CN

^31^P NMR spectrum of compound **11a** in CD_3_CN

^1^H NMR spectrum of compound **11b** in CD_3_CN

^13^C NMR spectrum of compound **11b** in CD_3_CN

^19^F NMR spectrum of compound **11b** in CD_3_CN

^31^P NMR spectrum of compound **11b** in CD_3_CN

^1^H NMR spectrum of compound **12a** in DMSO-*d*_6_

^13^C NMR spectrum of compound **12a** in DMSO-*d*_6_

^19^F NMR spectrum of compound **12a** in DMSO-*d*_6_

^31^P NMR spectrum of compound **12a** in DMSO-*d*_6_

^1^H NMR spectrum of compound **12b** in DMSO-*d*_6_

^13^C NMR spectrum of compound **12b** in DMSO-*d*_6_

^19^F NMR spectrum of compound **12b** in DMSO-*d*_6_

^31^P NMR spectrum of compound **12b** in DMSO-*d*_6_

^1^H NMR spectrum of compound **13a** in D_2_O

^13^C NMR spectrum of compound **13a** in D_2_O

^19^F NMR spectrum of compound **13a** in D_2_O

^31^P NMR spectrum of compound **13a** in D_2_O

^1^H NMR spectrum of compound **13b** in DMSO-*d*_6_

^13^C NMR spectrum of compound **13b** in DMSO-*d*_6_

^19^F NMR spectrum of compound **13b** in DMSO-*d*_6_

^31^P NMR spectrum of compound **13b** in DMSO-*d*_6_

^1^H NMR spectrum of compound **15a** in DMSO-*d*_6_

^13^C NMR spectrum of compound **15a** in DMSO-*d*_6_

^19^F NMR spectrum of compound **15a** in DMSO-*d*_6_

^31^P NMR spectrum of compound **15a** in DMSO-*d*_6_

^1^H NMR spectrum of compound **15b** in DMSO-*d*_6_

^13^C NMR spectrum of compound **15b** in DMSO-*d*_6_

^19^F NMR spectrum of compound **15b** in DMSO-*d*_6_

^31^P NMR spectrum of compound **15b** in DMSO-*d*_6_

^1^H NMR spectrum of compound **16a** in DMSO-*d*_6_

^13^C NMR spectrum of compound **16a** in DMSO-*d*_6_

^31^P NMR spectrum of compound **16a** in DMSO-*d*_6_

^1^H NMR spectrum of compound **16b** in DMSO-*d*_6_

^13^C NMR spectrum of compound **16b** in DMSO-*d*_6_

^31^P NMR spectrum of compound **16b** in DMSO-*d*_6_

^19^F NMR spectrum of compound **17a** in CD_3_CN

^31^P NMR spectrum of compound **17a** in CD_3_CN

^19^F NMR spectrum of compound **17b** in CD_3_CN

^31^P NMR spectrum of compound **17b** in CD_3_CN

^1^H NMR spectrum of compound **18a** in DMSO-*d*_6_

^13^C NMR spectrum of compound **18a** in DMSO-*d*_6_

^19^F NMR spectrum of compound **18a** in DMSO-*d*_6_

^31^P NMR spectrum of compound **18a** in DMSO-*d*_6_

^1^H NMR spectrum of compound **18b** in DMSO-*d*_6_

^13^C NMR spectrum of compound **18b** in DMSO-*d*_6_

^19^F NMR spectrum of compound **18b** in DMSO-*d*_6_

^31^P NMR spectrum of compound **18b** in DMSO-*d*_6_

^1^H NMR spectrum of compound **19a** in DMSO-*d*_6_

^13^C NMR spectrum of compound **19a** in DMSO-*d*_6_

^19^F NMR spectrum of compound **19a** in DMSO-*d*_6_

^31^P NMR spectrum of compound **19a** in DMSO-*d*_6_

^1^H NMR spectrum of compound **19b** in DMSO-*d*_6_

^13^C NMR spectrum of compound **19b** in DMSO-*d*_6_

^19^F NMR spectrum of compound **19b** in DMSO-*d*_6_

^31^P NMR spectrum of compound **19b** in DMSO-*d*_6_

^1^H NMR spectrum of compound **21a** in DMSO-*d*_6_

^13^C NMR spectrum of compound **21a** in DMSO-*d*_6_

^19^F NMR spectrum of compound **21a** in DMSO-*d*_6_

^31^P NMR spectrum of compound **21a** in DMSO-*d*_6_

^1^H NMR spectrum of compound **21b** in DMSO-*d*_6_

^13^C NMR spectrum of compound **21b** in DMSO-*d*_6_

^19^F NMR spectrum of compound **21b** in DMSO-*d*_6_

^31^P NMR spectrum of compound **21b** in DMSO-*d*_6_

^1^H NMR spectrum of compound **22a** in DMSO-*d*_6_

^13^C NMR spectrum of compound **22a** in DMSO-*d*_6_

^19^F NMR spectrum of compound **22a** in DMSO-*d*_6_

^31^P NMR spectrum of compound **22a** in DMSO-*d*_6_

^1^H NMR spectrum of compound **22b** in DMSO-*d*_6_

^13^C NMR spectrum of compound **22b** in DMSO-*d*_6_

^19^F NMR spectrum of compound **22b** in DMSO-*d*_6_

^31^P NMR spectrum of compound **22b** in DMSO-*d*_6_

^1^H NMR spectrum of compound **23a** in CD_3_CN

^13^C NMR spectrum of compound **23a** in CD_3_CN

^19^F NMR spectrum of compound **23a** in CD_3_CN

^31^P NMR spectrum of compound **23a** in CD_3_CN

^1^H NMR spectrum of compound **23b** in CD_3_CN

^13^C NMR spectrum of compound **23b** in CD_3_CN

^19^F NMR spectrum of compound **23b** in CD_3_CN

^31^P NMR spectrum of compound **23b** in CD_3_CN

^1^H NMR spectrum of compound **25a** in D_2_O

^13^C NMR spectrum of compound **25a** in D_2_O

^19^F NMR spectrum of compound **25a** in D_2_O

^31^P NMR spectrum of compound **25a** in D_2_O

^1^H NMR spectrum of compound **25b** in D_2_O

^13^C NMR spectrum of compound **25b** in D_2_O

^19^F NMR spectrum of compound **25b** in D_2_O

^31^P NMR spectrum of compound **25b** in D_2_O

^1^H NMR spectrum of compound **27a** in DMSO-*d*_6_

^31^P NMR spectrum of compound **27a** in DMSO-*d*_6_

^1^H NMR spectrum of compound **27b** in DMSO-*d*_6_

^31^P NMR spectrum of compound **27b** in DMSO-*d*_6_

^1^H NMR spectrum of compound **28a** in DMSO-*d*_6_

^13^C NMR spectrum of compound **28a** in DMSO-*d*_6_

^31^P NMR spectrum of compound **28a** in DMSO-*d*_6_

^1^H NMR spectrum of compound **28b** in DMSO-*d*_6_

^13^C NMR spectrum of compound **28b** in DMSO-*d*_6_

^31^P NMR spectrum of compound **28b** in DMSO-*d*_6_

^1^H NMR spectrum of compound **29a** in CD_3_CN

^13^C NMR spectrum of compound **29a** in CD_3_CN

^31^P NMR spectrum of compound **29a** in CD_3_CN

^1^H NMR spectrum of compound **29b** in CD_3_CN

^13^C NMR spectrum of compound **29b** in CD_3_CN

^31^P NMR spectrum of compound **29b** in CD_3_CN

^1^H NMR spectrum of compound **30a** in DMSO-*d*_6_

^13^C NMR spectrum of compound **30a** in DMSO-*d*_6_

^31^P NMR spectrum of compound **30a** in DMSO-*d*_6_

^1^H NMR spectrum of compound **30b** in DMSO-*d*_6_

^13^C NMR spectrum of compound **30b** in DMSO-*d*_6_

^31^P NMR spectrum of compound **30b** in DMSO-*d*_6_

^1^H NMR spectrum of compound **31a** in D_2_O

^13^C NMR spectrum of compound **31a** in D_2_O

^31^P NMR spectrum of compound **31a** in D_2_O

^1^H NMR spectrum of compound **31b** in D_2_O

^13^C NMR spectrum of compound **31b** in D_2_O

^31^P NMR spectrum of compound **31b** in D_2_O

^1^H NMR spectrum of compound **33a** in CD_3_CN

^13^C NMR spectrum of compound **33a** in CD_3_CN

^19^F NMR spectrum of compound **33a** in CD_3_CN

^31^P NMR spectrum of compound **33a** in CD_3_CN

^1^H NMR spectrum of compound **33b** in CD_3_CN

^13^C NMR spectrum of compound **33b** in CD_3_CN

^19^F NMR spectrum of compound **33b** in CD_3_CN

^31^P NMR spectrum of compound **33b** in DMSO-*d*_6_

^1^H NMR spectrum of compound **34a** in DMSO-*d*_6_

^13^C NMR spectrum of compound **34a** in DMSO-*d*_6_

^19^F NMR spectrum of compound **34a** in DMSO-*d*_6_

^31^P NMR spectrum of compound **34a** in DMSO-*d*_6_

^1^H NMR spectrum of compound **34b** in DMSO-*d*_6_

^13^C NMR spectrum of compound **34b** in DMSO-*d*_6_

^19^F NMR spectrum of compound **34b** in DMSO-*d*_6_

^31^P NMR spectrum of compound **34b** in DMSO-*d*_6_

^1^H NMR spectrum of compound **35a** in CD_3_CN

^13^C NMR spectrum of compound **35a** in CD_3_CN

^19^F NMR spectrum of compound **35a** in CD_3_CN

^31^P NMR spectrum of compound **35a** in CD_3_CN

^1^H NMR spectrum of compound **35b** in CD_3_CN

^13^C NMR spectrum of compound **35b** in CD_3_CN

^19^F NMR spectrum of compound **35b** in CD_3_CN

^31^P NMR spectrum of compound **35b** in CD_3_CN

^1^H NMR spectrum of compound **36a** in DMSO-*d*_6_

^13^C NMR spectrum of compound **36a** in DMSO-*d*_6_

^19^F NMR spectrum of compound **36a** in DMSO-*d*_6_

^31^P NMR spectrum of compound **36a** in DMSO-*d*_6_

^1^H NMR spectrum of compound **36b** in DMSO-*d*_6_

^13^C NMR spectrum of compound **36b** in DMSO-*d*_6_

^19^F NMR spectrum of compound **36b** in DMSO-*d*_6_

^31^P NMR spectrum of compound **36b** in DMSO-*d*_6_

^1^H NMR spectrum of compound **37a** in D_2_O

^13^C NMR spectrum of compound **37a** in D_2_O

^19^F NMR spectrum of compound **37a** in D_2_O

^31^P NMR spectrum of compound **37a** in D_2_O

^1^H NMR spectrum of compound **37b** in D_2_O

^13^C NMR spectrum of compound **37b** in D_2_O

^19^F NMR spectrum of compound **37b** in D_2_O

^31^P NMR spectrum of compound **37b** in D_2_O

^1^H NMR spectrum of compound **39** (diastereomeric mixture) in DMSO-*d*_6_

^13^C NMR spectrum of compound **39** (diastereomeric mixture) in DMSO-*d*_6_

^31^P NMR spectrum of compound **39** (diastereomeric mixture) in DMSO-*d*_6_

^1^H NMR spectrum of compound **40a** in DMSO-*d*_6_

^13^C NMR spectrum of compound **40a** in DMSO-*d*_6_

^31^P NMR spectrum of compound **40a** in DMSO-*d*_6_

^1^H NMR spectrum of compound **40b** in DMSO-*d*_6_

^13^C NMR spectrum of compound **40b** in DMSO-*d*_6_

^31^P NMR spectrum of compound **40b** in DMSO-*d*_6_

^1^H NMR spectrum of compound **41a** in CD_3_CN

^13^C NMR spectrum of compound **41a** in CD_3_CN

^31^P NMR spectrum of compound **41a** in CD_3_CN

^1^H NMR spectrum of compound **41b** in CD_3_CN

^13^C NMR spectrum of compound **41b** in CD_3_CN

^31^P NMR spectrum of compound **41b** in CD_3_CN

^1^H NMR spectrum of compound **42a** in DMSO-*d*_6_

^13^C NMR spectrum of compound **42a** in DMSO-*d*_6_

^31^P NMR spectrum of compound **42a** in DMSO-*d*_6_

^1^H NMR spectrum of compound **42b** in DMSO-*d*_6_

^13^C NMR spectrum of compound **42b** in DMSO-*d*_6_

^31^P NMR spectrum of compound **42b** in DMSO-*d*_6_

^1^H NMR spectrum of compound **43a** in D_2_O

^13^C NMR spectrum of compound **43a** in D_2_O

^31^P NMR spectrum of compound **43a** in D_2_O

^1^H NMR spectrum of compound **43b** in D_2_O

^13^C NMR spectrum of compound **43b** in D_2_O

^31^P NMR spectrum of compound **43b** in D_2_O

1. ***Exonuclease study for fully deprotected, stereo-defined dinucleotides***

**Snake venom phosphodiesterase assay**

A 1 mg/mL solution in H_2_O (nuclease free) of each chiral pure phosphorothioate dinucleotide was prepared, incubated SVPD at 37 °C, and analyzed by reverse-phase HPLC at 48 h. For control sample, enzyme was replaced by H_2_O. Reverse-phase HPLC conditions: xBridge^TM^ C18, 3.5 µm, 4.6 x 150 mm column, flow 1.2 mL/min Buffer B was acetonitrile, Buffer A was ddH_2_O, gradient 3–30% Buffer B in 6 min or 3–15% Buffer B in 6 min. With the exception of the g•a dinucleotide, the diastereomer that migrated further on TLC (top spot) was degraded under the assay conditions, whereas the slower-migrating diastereomer (lower spot) was more stable. Based on previous reports, the *R*_p_ isomer is less stable than the *S*_p_ isomer in the presence of SVPD (2,5).

**Phosphodiesterease II assay**

## A 1 mg/mL solution in H_2_O (nuclease free) of each chiral pure phosphorothioate dinucleotide was prepared, incubated with X U/mL PDII for 48 h or 5 days at 37 °C and analyzed by reverse-phase HPLC. As a control, enyzme was replaced with H_2_O. The reverse-phase HPLC conditions were as follows: xBridgeTM C18, 3.5 µm, 4.6 x 150 mm column, Buffer B was acetonitrile, Buffer A was ddH_2_O, flow 1.2 mL/min, gradient 3–30% Buffer B in 6 min or 3–15% Buffer B in 6 min. With the exception of the g•a dinucleotide, the diastereomer that migrated more rapidly on TLC was either more stable or showed no stability difference compared to the slower migrating diastereomer. The Rp isomer was more stable in the presence of PDII than the *S*p isomer, particularly when the 5′ terminal nucleotide is 2′F modified.

1. ***Methods of assignment of absolute configurations for synthesized stereo-defined oligonucleotide***

For stereo-defined oligonucleotides that were synthesized using the stereo-defined phosphoramidite building blocks, ^31^P-NMR could be used to determine whether only the *R*_p_ or *S*_p_ isomer was present in the final purified compounds or whether a mixture of *R*_p_ and *S*_p_ isomers were present. IEX analysis could also be utilized to confirm retention time differences between *R*_p_ and *S*_p_ isomers. To do this, compounds containing different configurations were mixed in a set ratio (for instance, 75:25) to determine which isomer eluted first. In some cases, the two mixed isomers co-eluted and no determination could be made. This was performed DMT-on and DMT-off for the 5ʹ end isomers, to visualize the “flip” described in the “***Methods of assignment of stereochemical configurations for purified isomers***” section. Incubation of the antisense strands with the 3ʹ exonuclease, SVPD, which preferentially degrades *R*_p_ isomers, was also utilized to provide further evidence of 3ʹ end *R*_p_ or *S*_p_ assignment (2,5). The 5ʹ exonuclease, PDII was only diagnostic when the PS was in combination with a 2ʹ-F modified base.

In all cases in Table S4, the top spot was found to correspond to the *R*_p_ isomer and the lower spot to the *S*_p_ isomer except in the case of the g●a dimer separation. The g●a dimer was separated by reverse phase, while all others were separated by normal phase.

Table S4. Oligonucleotides synthesized using the stereo-defined dinucleotides^a^

| Strand ID | Oligonucleotide Sequence (5′-3′) | Target | S/AS | Chiral “Spot” Used |
| --- | --- | --- | --- | --- |
| si1-S-FR | A**R**aCaGuGuUCUuGcUcUaUaA(L) | *Ttr* | S | 5′ end: top spot |
| si1-S-FS | A**S**aCaGuGuUCUuGcUcUaUaA(L) | *Ttr* | S | 5′ end: lower spot |
| si3-S | a**R**aCaGuGuUCUuGcUcUaUaA(L) | *Ttr* | S | 5′ end: top spot |
| si11-S | a**S**aCaGuGuUCUuGcUcUaUaA(L) | *Ttr* | S | 5′ end: lower spot |
| si3-AS | u**R**UaUaGaGcAagaAcAcUgUuu**R**u | *Ttr* | AS | 5′ end: top spot  3′ end: top spot |
| si5-AS | u**R**UaUaGaGcAagaAcAcUgUuu**S**u | *Ttr* | AS | 5′ end: top spot  3′ end: lower spot |
| si7-AS | u**S**UaUaGaGcAagaAcAcUgUuu**R**u | *Ttr* | AS | 5′ end: lower spot  3′ end: top spot |
| si9-AS | u**S**UaUaGaGcAagaAcAcUgUuu**S**u | *Ttr* | AS | 5′ end: lower spot  3′ end: lower spot |
| si12-S | g**S**aCaAaAuAACuCaCuAuAaU(L) | *C5* | S | 5′ end: top spot |
| si4-S | g**R**aCaAaAuAACuCaCuAuAaU(L) | *C5* | S | 5′ end: lower spot |
| si4-AS | a**R**UuAuAgUgAguuAuUuUgUca**R**a | *C5* | AS | 5′ end: top spot  3′ end: top spot |
| si6-AS | a**R**UuAuAgUgAguuAuUuUgUca**S**a | *C5* | AS | 5′ end: top spot  3′ end: lower spot |
| si8-AS | a**S**UuAuAgUgAguuAuUuUgUca**R**a | *C5* | AS | 5′ end: lower spot  3′ end: top spot |
| si10-AS | a**S**UuAuAgUgAguuAuUuUgUca**S**a | *C5* | AS | 5′ end: lower spot  3′ end: lower spot |

*^a^*Uppercase and lowercase letters represent 2′-F-RNA and 2′-OMe, respectively. (L) represents GalNAc ligand (1). Phosphorothioate mixtures are indicated by the ● and *R*_p_ and *S*_p_ linkages are indicated by **R** and **S**, respectively.

***Identification of mrTTR oligonucleotides synthesized using stereo-defined dinucleotides***

Table S5 summarizes the configurations of the *Ttr*-targeted siRNAs used in this study. The sense strands contained 5′ end isomers that separated with DMT-on as well as with DMT-off. The “flip” in elution time during IEX analysis was observed for isomers of both the sense strands with both 2′-F and 2′-OMe 5′-terminal residues when the crude compounds were mixed at various ratios. The antisense strands contained 5′-end isomers that separated with DMT-on only. The oligonucleotide with the PS in the *S*_p_ configuration was predicted to elute earlier than that with *R*_p_ with DMT-on. However, the “flip” in elution time was not observed during IEX analysis as DMT-off strands co-eluted.

Table S5. Predicted 5′-end and 3′-end configurations of Ttr-targeted siRNAs

| Strand ID | Predicted 5′-end Configuration | Methods Used | Predicted 3′-end Configuration | Methods Used |
| --- | --- | --- | --- | --- |
| si1-S-FR | *R*_p_ (top spot) | DMT-off IEX  DMT-on IEX | —*^a^* | —*^a^* |
| si1-S-FS | *S*_p_ (lower spot) | DMT-off IEX  DMT-on IEX | —*^a^* | —*^a^* |
| si3-S | *R*_p_ (top spot) | DMT-off IEX  DMT-on IEX | —*^a^* | —*^a^* |
| si11-S | *S*_p_ (lower spot) | DMT-off IEX  DMT-on IEX | —*^a^* | —*^a^* |
| si3-AS | *R*_p_ (top spot) | DMT-on IEX | *R*_p_ (top spot) | SVPD assay |
| si5-AS | *R*_p_ (top spot) | DMT-on IEX | *S*_p_ (lower spot) | SVPD assay |
| si7-AS | *S*_p_ (lower spot) | DMT-on IEX | *R*_p_ (top spot) | SVPD assay |
| si9-AS | *S*_p_ (lower spot) | DMT-on IEX | *S*_p_ (lower spot) | SVPD assay |

*^a^*PS linkage is absent on 3′-end of sense strands of *Ttr*-targeted siRNAs.

***Identification of C5 oligonucleotides synthesized using stereo-defined dinucleotides***

Table S6 summarizes the methods used to predict configurations of the *C5*-targeted isomers that were synthesized using the stereo-defined dinucleotide phosphoramidites. The oligonucleotides designed to target the *C5* mRNA made with the chirally pure dinucleotides were synthesized DMT-off for IEX purification. No analysis of the compounds was performed with DMT-on. The predicted 5′-end *R*_p_ isomer elutes earlier than the *S*_p_ isomer with DMT-off.

Table S6. Predicted 5′-end and 3′-end configurations of C5-targeted siRNAs.

| Strand ID | Predicted 5′-end Configuration | Methods Used | Predicted 3′-end Configuration | Methods Used |
| --- | --- | --- | --- | --- |
| si12-S*^a^* | *S*_p_ (top spot)*^a^* | ^31^P-NMR  DMT-off IEX | —*^b^* | —*^b^* |
| si4-S*^a^* | *R*_p_ (lower spot)*^a^* | ^31^P-NMR  DMT-off IEX | —*^b^* | —*^b^* |
| si4-AS | *R*_p_ (top spot) | ^31^P-NMR | *R*_p_ (top spot) | ^31^P-NMR  DMT-off IEX |
| si6-AS | *R*_p_ (top spot) | ^31^P-NMR | *S*_p_ (lower spot) | ^31^P-NMR  DMT-off IEX |
| si8-AS | *S*_p_ (lower spot) | ^31^P-NMR | *R*_p_ (top spot) | ^31^P-NMR  DMT-off IEX |
| si10-AS | *S*_p_ (lower spot) | ^31^P-NMR | *S*_p_ (lower spot) | ^31^P-NMR  DMT-off IEX |

*^a^*si12-S and si4-S are the sense strands synthesized with the g•a dimer. This is the only instance found where compound with the higher Rf value did not correspond to the *R*_p_ configuration*. ^b^*PS linkage is absent on 3′-end of sense strands of *C5*-targeted siRNAs.

1. ***Oligonucleotide characterization***

Table S7. Mass spectroscopy of *Ttr*- and *C5*-targeted siRNA strands used in this study*^a^*

| Target | Duplex | Strand ID | Config. | Sequence (5′-3′) | Strand | Mass (m/z) | |
| --- | --- | --- | --- | --- | --- | --- | --- |
|  |  |  |  |  |  | calc. | obs. |
| TTR | si1 | si1-S  si1-AS | X  X-X | a•aCaGuGuUCUuGcUcUaUaA(L)  u•UaUaGaGcAagaAcAcUgUuu•u | sense  antisense | 8586.1  7563.8 | 8584.6  7562.6 |
|  | si3 | si3-S  si3-AS | R  R-R | a**R**aCaGuGuUCUuGcUcUaUaA(L)  u**R**UaUaGaGcAagaAcAcUgUuu**R**u | sense  antisense | 8586.1  7563.8 | 8584.9  7562.9 |
|  | si5 | si5-S  si5-AS | R  R-S | a**R**aCaGuGuUCUuGcUcUaUaA(L)  u**R**UaUaGaGcAagaAcAcUgUuu**S**u | sense  antisense | 8586.1  7563.8 | 8584.9  7562.6 |
|  | si7 | si7-S  si7-AS | R  S-R | a**R**aCaGuGuUCUuGcUcUaUaA(L)  u**S**UaUaGaGcAagaAcAcUgUuu**R**u | sense  antisense | 8586.1  7563.8 | 8584.9  7562.9 |
|  | si9 | si9-S  si9-AS | R  S-S | a**R**aCaGuGuUCUuGcUcUaUaA(L)  u**S**UaUaGaGcAagaAcAcUgUuu**S**u | sense  antisense | 8586.1  7563.8 | 8584.9  7562.6 |
|  | si11 | si11-S  si11-AS | S  R-R | a**S**aCaGuGuUCUuGcUcUaUaA(L)  u**R**UaUaGaGcAagaAcAcUgUuu**R**u | sense  antisense | 8586.1  7563.8 | 8584.8  7562.9 |
|  | si13 | si13-S  si13-AS | S  R-S | a**S**aCaGuGuUCUuGcUcUaUaA(L)  u**R**UaUaGaGcAagaAcAcUgUuu**S**u | sense  antisense | 8586.1  7563.8 | 8584.8  7562.6 |
|  | si15 | si15-S  si15-AS | S  S-R | a**S**aCaGuGuUCUuGcUcUaUaA(L)  u**S**UaUaGaGcAagaAcAcUgUuu**R**u | sense  antisense | 8586.1  7563.8 | 8584.8  7562.9 |
|  | si17 | si17-S  si17-AS | S  S-S | a**S**aCaGuGuUCUuGcUcUaUaA(L)  u**S**UaUaGaGcAagaAcAcUgUuu**S**u | sense  antisense | 8586.1  7563.8 | 8584.8  7562.6 |
|  | si19 | si19-S  si19-AS | XX  XX-XX | a•a•CaGuGuUCUuGcUcUaUaA(L)  u•U•aUaGaGcAagaAcAcUgUu•u•u | sense  antisense | 8602.2  7595.9 | 8601.2  7594.2 |
|  | N/A | si1-S-F | X | A•aCaGuGuUCUuGcUcUaUaA(L) | sense | 8574.1 | 8572.9 |
|  | N/A | si1-S-FR | R | A**R**aCaGuGuUCUuGcUcUaUaA(L) | sense | 8574.1 | 8572.9 |
|  | N/A | si1-S-FS | S | A**S**aCaGuGuUCUuGcUcUaUaA(L) | sense | 8574.1 | 8572.9 |
| C5 | si2 | si2-S  si2-AS | X  X-X | g•aCaAaAuAACuCaCuAuAaU(L)  a•UuAuAgUgAguuAuUuUgUca•a | sense  antisense | 8623.3  7542.7 | 8621.9  7541.3 |
|  | si4 | si4-S  si4-AS | R  R-R | g**R**aCaAaAuAACuCaCuAuAaU(L)  a**R**UuAuAgUgAguuAuUuUgUca**R**a | sense  antisense | 8623.3  7542.7 | 8621.0  7540.8 |
|  | si6 | si6-S  si6-AS | R  R-S | g**R**aCaAaAuAACuCaCuAuAaU(L)  a**R**UuAuAgUgAguuAuUuUgUca**S**a | sense  antisense | 8623.3  7542.7 | 8621.0  7541.2 |
|  | si8 | si8-S  si8-AS | R  S-R | g**R**aCaAaAuAACuCaCuAuAaU(L)  a**S**UuAuAgUgAguuAuUuUgUca**R**a | sense  antisense | 8623.3  7542.7 | 8621.0  7540.5 |
|  | si10 | si10-S  si10-AS | R  S-S | g**R**aCaAaAuAACuCaCuAuAaU(L)  a**S**UuAuAgUgAguuAuUuUgUca**S**a | sense  antisense | 8623.3  7542.7 | 8621.0  7540.5 |
|  | si12 | si12-S  si12-AS | S  R-R | g**S**aCaAaAuAACuCaCuAuAaU(L)  a**R**UuAuAgUgAguuAuUuUgUca**R**a | sense  antisense | 8623.3  7542.7 | 8621.0  7540.8 |
|  | si14 | si14-S  si14-AS | S  R-S | g**S**aCaAaAuAACuCaCuAuAaU(L)  a**R**UuAuAgUgAguuAuUuUgUca**S**a | sense  antisense | 8623.3  7542.7 | 8621.0  7541.2 |
|  | si16 | si16-S  si16-AS | S  S-R | g**S**aCaAaAuAACuCaCuAuAaU(L)  a**S**UuAuAgUgAguuAuUuUgUca**R**a | sense  antisense | 8623.3  7542.7 | 8621.0  7540.5 |
|  | si18 | si18-S  si18-AS | S  S-S | g**S**aCaAaAuAACuCaCuAuAaU(L)  a**S**UuAuAgUgAguuAuUuUgUca**S**a | sense  antisense | 8623.3  7542.7 | 8621.0  7540.5 |
|  | si20 | si20-S  si20-AS | XX  XX-XX | G•a•CaAaAuAACuCaCuAuAaU(L)  a•U•uAuAgUgAguuAuUuUgUc•a•a | sense  antisense | 8627.3  7574.9 | 8626.3  7573.7 |
|  | N/A | si2-S-F | X | G•aCaAaAuAACuCaCuAuAaU(L) | sense | 8611.2 | 8609.6 |
|  | N/A | si2-S-FR | R | G**R**aCaAaAuAACuCaCuAuAaU(L) | sense | 8611.2 | 8609.2 |
|  | N/A | si2-S-FS | S | G**S**aCaAaAuAACuCaCuAuAaU(L) | sense | 8611.2 | 8609.3 |

*^a^*Uppercase and lowercase letters represent 2′-F-RNA and 2′-OMe, respectively. (L) represents the GalNAc ligand (1). Phosphorothioate mixtures are indicated by the ●, and *R*_p_ and *S*_p_ linkages are indicated by **R** and **S**, respectively.

**
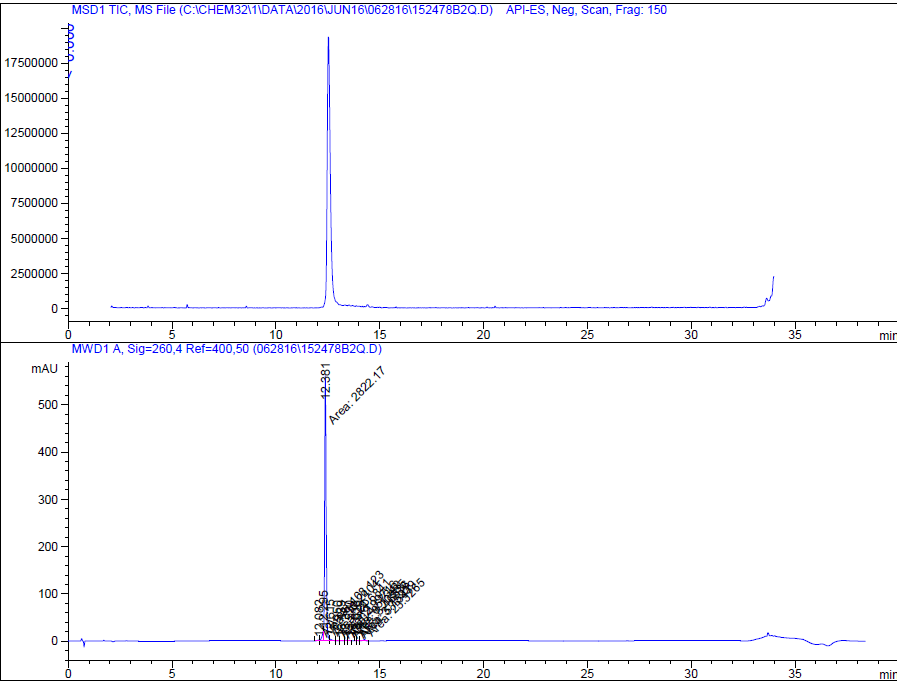
**

**
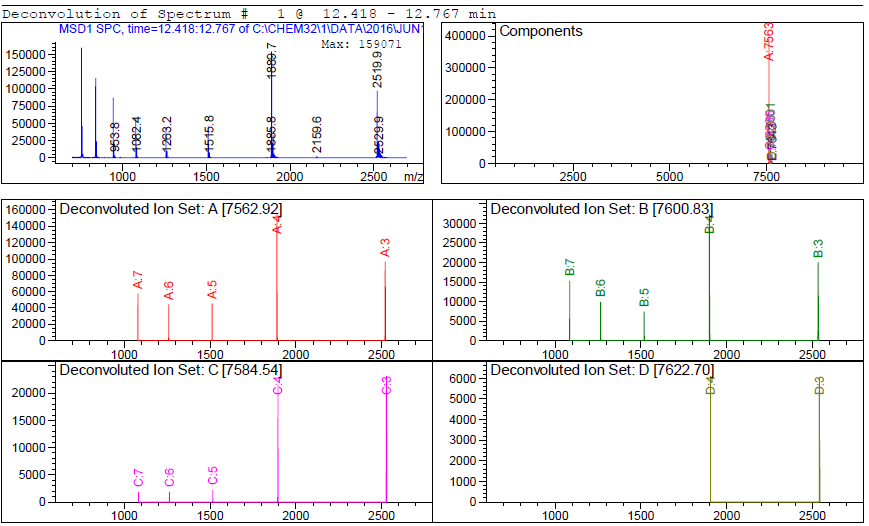
**

**
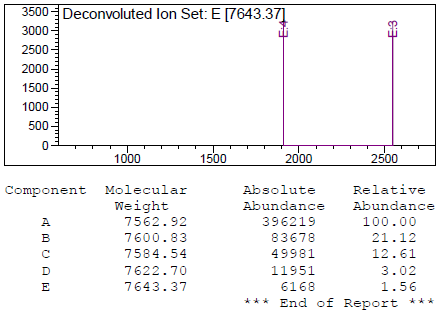
**

Figure S21. Example LC-MS spectrum and mass components of oligonucleotide si3-AS and si11-AS.

1. ***Analysis of whether stereo-defined oligonucleotides serve as Clp1 kinase substrates***

To test differences in the ability of Clp1 kinase to phosphorylate oligonucleotides containing an *R*_p_ or *S*_p_ phosphorothiate linkage on the 5′ end, the oligonucleotides shown in Table S8 were synthesized. Compounds si1-AS-RNA2 and si1-AS-RNA3 were separated from DMT-on synthesis of si1-AS-RNA4 by the same method used in “***Purification of sense strands si2-S-F and si2-S into two diastereomers***” and identified using ^31^P-NMR analysis and DMT-on/off anion exchange analysis as described in “***Identification of mrTTR isomers synthesized using stereo-defined dinucleotides***”. In order to preserve the 5′ DMT group, deprotection using tetrabutylammonium fluoride (TBAF) was used. Solid support was incubated at 65 °C in ethanol:ammonia (1:4) for 5 h, filtered, washed with ethanol, and dried. The resulting powder was dissolved in 1M TBAF and shaken for 30 h at room temperature.

The four fully ribo-*TTR* targeted antisense strands were annealed to fully ribo sense strand si1-S-RNA. Phosphorylation by Clp1 kinase was evaluated by mixing 100 pmol (10 μL 10 μM stock) of appropriate duplex and incubating at 30 °C for 1 h in the presence of a range of concentrations of Clp1. The reaction was stopped by heating at 95 °C for 5 min. Clp1 was removed using 30-kD MWCO spin filters. The supernatant was diluted to 100 μL with water, and the sample was added to a spin filter column, spun at 14,000 *x g* for 15 min, and analyzed by reverse phase LC-MS analysis to quantify phosphorylation (Figure S22). The optimal substrate was the oligonucleotide with a phosphodiester linkage at the 5′ end, and the kinase did not discriminate between PS diastereomers.

Table S8. Oligonucleotides synthesized to evaluate phosphorylation

| **Strand ID** | **Sequence (5ʹ-3ʹ)***^a^* | **Mass (m/z)** | |
| --- | --- | --- | --- |
|  |  | **calc.** | **obs.** |
| si1-S-RNA  si1-AS-RNA1  si1-AS-RNA2  si1-AS-RNA3  si1-AS-RNA4 | r(*AACAGUGUUCUUGCUCUAUAA*) r(*UUAUAGAGCAAGAACACUGUUUU*)  r(*U***R***UAUAGAGCAAGAACACUGUUUU*)  r(*U***S***UAUAGAGCAAGAACACUGUUUU*)  r(*U*•*UAUAGAGCAAGAACACUGUUUU*) | 6619.0  7317.4  7333.5  7333.5  7333.5 | 6617.7  7316.1  7332.8  7332.8  7332.1 |

Figure S22. Clp1 kinase does not discriminate between *R*_p_ and *S*_p_ stereoisomers.

# ***References***

1. Nair, J.K., Willoughby, J.L.S., Chan, A., Charisse, K., Alam, M.R., Wang, Q., Hoekstra, M., Kandasamy, P., Kel’in, A.V., Milstein, S. *et al.* (2014) Multivalent N-Acetylgalactosamine-Conjugated siRNA Localizes in Hepatocytes and Elicits Robust RNAi-Mediated Gene Silencing. *J. Am. Chem. Soc.*, **136**, 16958-16961.

2. Eckstein, F. (1983) Phosphorothioate Analogues of Nucleotides—Tools for the Investigation of Biochemical Processes. *Angew. Chem. Int. Ed. Engl.*, **22**, 423-439.

3. Almer, H., Stawinski, J., Stroemberg, R. and Thelin, M. (1992) Synthesis of diribonucleoside phosphorothioates via stereospecific sulfuration of H-phosphonate diesters. *J. Org. Chem.*, **57**, 6163-6169.

4. Ravikumar, V.T. and Cole, D.L. (2003) Diastereomeric Process Control in the Synthesis of 2′-O-(2-Methoxyethyl) Oligoribonucleotide Phosphorothioates as Antisense Drugs. *Nucleosides, Nucleotides & Nucleic Acids*, **22**, 1639-1645.

5. Sobkowski, M., Jankowska, J., Kraszewski, A. and Stawinski, J. (2005) Stereochemistry of Internucleotide Bond Formation by the H-Phosphonate Method. 1. Synthesis and 31P Nmr Analysis of 16 Diribonulceoside (3′-5′)-H-Phosphonates and the Corresponding Phosphorothioates. *Nucleosides, Nucleotides & Nucleic Acids*, **24**, 1469-1484.

6. Jahns, H., Roos, M., Imig, J., Baumann, F., Wang, Y., Gilmour, R. and Hall, J. (2015) Stereochemical bias introduced during RNA synthesis modulates the activity of phosphorothioate siRNAs. *Nat. Commun.*, **6**, 6317.

7. Oka, N., Yamamoto, M., Sato, T. and Wada, T. (2008) Solid-Phase Synthesis of Stereoregular Oligodeoxyribonucleoside Phosphorothioates Using Bicyclic Oxazaphospholidine Derivatives as Monomer Units. *J. Am. Chem. Soc.*, **130**, 16031-16037.

8. Li, M., Lightfoot, H.L., Halloy, F., Malinowska, A.L., Berk, C., Behera, A., Schümperli, D. and Hall, J. (2017) Synthesis and cellular activity of stereochemically-pure 2′-O-(2-methoxyethyl)-phosphorothioate oligonucleotides. *Chem. Commun.*, **53**, 541-544.

9. Wan, W.B., Migawa, M.T., Vasquez, G., Murray, H.M., Nichols, J.G., Gaus, H., Berdeja, A., Lee, S., Hart, C.E., Lima, W.F. *et al.* (2014) Synthesis, biophysical properties and biological activity of second generation antisense oligonucleotides containing chiral phosphorothioate linkages. *Nucleic Acids Res.*, **42**, 13456-13468.
